# Intramolecular arylsulfide-coordinated diboraanthracenes: effect of B–S coordination on ground-state and excited-state behavior†

**DOI:** 10.1039/d5sc01726b

**Published:** 2025-04-14

**Authors:** Hiroki Narita, Alexander Virovets, Hans-Wolfram Lerner, Matthias Wagner, Shigehiro Yamaguchi

**Affiliations:** a Department of Chemistry, Graduate School of Science, Research Center for Materials Science (RCMS), Integrated Research Consortium on Chemical Sciences (IRCCS), Nagoya University Furo-cho, Chikusa Nagoya 464-8602 Japan yamaguchi.shigehiro.r9@f.mail.nagoya-u.ac.jp; b Institut für Anorganische und Analytische Chemie, Goethe-Universität Frankfurt Max-von-Laue-Straße 7 60438 Frankfurt am Main Germany; c Institute of Transformative Bio-Molecules (WPI-ITbM), Nagoya University Furo-cho, Chikusa Nagoya 464-8601 Japan

## Abstract

Controlling boron–heteroatom interactions in triarylborane scaffolds can lead to stimuli-responsive photophysical properties. A key molecular design to this end is the utilization of a labile coordination bond between the boron atom and a Lewis basic heteroatom. Herein, we report the synthesis of a series of 9,10-dihydro-9,10-diboraanthracenes (DBAs) bearing *ortho*-arylthiomethyl-substituted phenyl groups on the boron atom as a new family of stimuli-responsive boron-containing π-conjugated molecules. The two *ortho*-arylthiomethyl groups coordinate to the boron atoms by forming five-membered rings in the DBA scaffolds to produce the *cis* isomers predominantly, where the strength of the boron–sulfur bonds can be tuned by structural and electronic modifications of the aryl groups. In the ground state, the B–S bond is cleaved upon heating in solution. In the excited state, the B–S bond undergoes dissociation, resulting in emission from tricoordinate species. The aryl groups on the sulfur atom also play a role in forming an intramolecular charge-transfer state, whereby the emissions are bathochromically shifted with large apparent Stokes shifts. Moreover, the B–S bonds are sensitive to solvent polarity and temperature, resulting in multiple emission properties depending on the surrounding environment.

## Abstract 2

Boron-embedded π-conjugated molecules with intramolecular sulfur coordination exhibit B–S bond dissociation not only thermally in the ground state but also upon light irradiation, resulting in intriguing photoluminescence properties.

## Graphical abstract

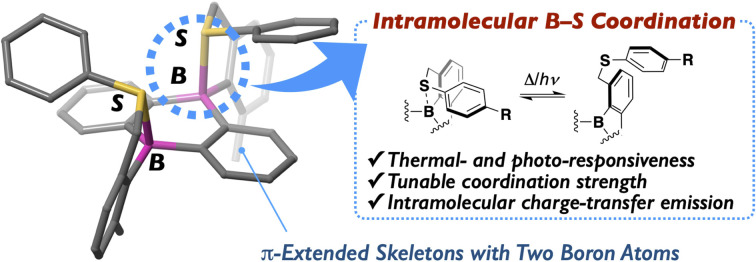

## Introduction

Organic π-conjugated compounds containing tricoordinate boron atoms have attracted much attention owing to their potential utilities in a wide range of applications,^1^ such as nonlinear optical materials,^2,3^ light-emitting materials,^4^ and fluorescent probes for bioimaging.^5–8^ The vacant p orbital of the boron atom in these molecules plays crucial roles in furnishing not only electron-accepting properties but also stimuli-responsive properties. In particular, boron-containing π-conjugated skeletons can form intermolecular Lewis acid–base complexes with various types of Lewis bases, for example, fluoride ions,^9^ cyanide ions,^10^ pyridine derivatives,^11^ and phosphine derivatives.^12^ The complexation impairs the electron-accepting ability of the boron moiety, resulting in substantial changes in their electronic structures and thereby photophysical properties, typically, hypsochromic shifts in the absorption and fluorescence spectra. By adjusting the balance between the Lewis acidity of the boron atom and the Lewis basicity of the base, the reversible switching between the tri- and tetracoordinate states is realized in some complexes in response to the surrounding environment or external stimuli, giving rise to various intriguing phenomena, such as thermochromism,^13^ solubility tuning,^14^ and photodissociation-induced dual emission.^15^

**Fig. 1:**
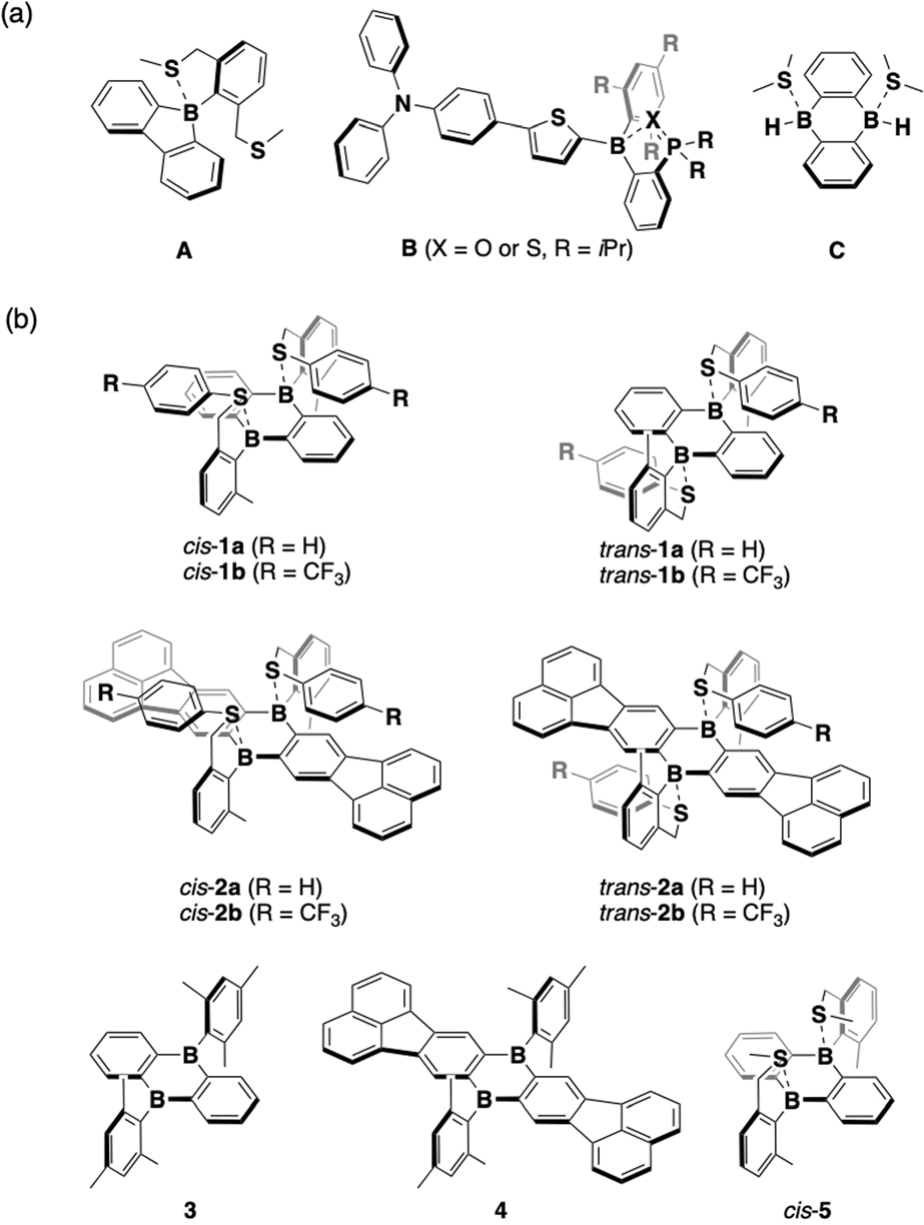
(a) Examples of boron-containing π-conjugated compounds with boron–sulfur coordination bonds. (b) Chemical structures of 1 and 2 and their reference compounds 3, 4, and *cis*-5 studied in this work.

A design strategy to form labile Lewis acid–base complexes is the introduction of a weak Lewis basic moiety into a triarylborane scaffold in an intramolecularly coordinating fashion. For this purpose, various coordinating groups have been utilized, such as –NR_2_,^16^ –OR,^17^ –C(R′)=O,^18^ –C(R′)=NR,^19^ or –P(=O)R_2_.^20^ As for intramolecularly sulfur-coordinated organoboranes, Rupar and coworkers reported a tetracoordinate borafluorene with a pincer-like aryl group (A), in which one of the sulfur atoms was coordinated to the boron center in the ground state, and the resulting B–S bond underwent dissociation in the excited state (Fig. 1a).^17^ Recently, we reported donor–π–acceptor (D–π–A)-type organoborane fluorophores bearing *ortho*-P=X-substituted phenyl groups on the boron atom (B; X = O or S).^20*d*^ The P=X groups also underwent photodissociation in the excited state, where the P=S derivative facilitated the dissociation compared with the P=O congeners. Thus, the B–S coordination bond can be expected to enable the formation of labile Lewis acid–base complexes with stimuli- or environment-responsiveness; however, examples of such complexes are still limited. To expand the application scope of this compound class, more in-depth knowledge of the boron–sulfur interaction is required.

In addition, as for the boron-containing π-conjugated scaffold, most of the intramolecular borane–Lewis base complexes reported so far contain only one boron atom, with only a few examples bearing more than two boron atoms.^19^ In this context, 9,10-dihydro-9,10-diboraanthracene (DBA) derivatives are promising scaffolds because of their rigid framework, in which the vacant p orbitals on two boron atoms are effectively π-conjugated with the 1,2-phenylene moieties.^21^ The photophysical properties of some DBA derivatives have been studied. For instance, Cheng and coworkers reported DBA-based D–A–D-type compounds exhibiting highly efficient thermally activated delayed fluorescence properties.^22^ Recently, one of our groups demonstrated that two laterally π-expanded DBA derivatives exhibited ultralong room-temperature phosphorescence in a rigid poly(methyl methacrylate) matrix.^23^ Although a few examples of DBAs complexed with externally added Lewis bases, such as a fluoride ion,^24^ pyridine,^25^ 1,2-diazine derivatives,^26^ and dimethylsulfide (C)^26^ have been reported, those are limited to intermolecular Lewis acid–base complexed systems.

To elucidate the impact of the B–S coordination bonds on the properties of boron-based π-electron systems, we synthesized in this study arylsulfide-substituted DBAs 1 and 2 as a new family of boron-based π-electron systems with intramolecular B–S coordination bonds (Fig. 1b). Arylthiomethyl groups were attached to the *ortho* position of the phenyl group on the boron atom to form a coordination bond in a five-membered ring fashion. Since the DBAs have two boron atoms, the intramolecular B–S coordination would form *cis* and *trans* isomers. To tune the intramolecular B–S coordination strength, an electron-withdrawing CF_3_ group was introduced at the *para* position of the arylthio moiety with respect to the sulfur atom. A comparison of their photophysical properties with those of mesityl-substituted tricoordinate congeners 3 and 4 and methylsulfide-coordinated DBA *cis*-5 confirmed that the arylsulfide–boron coordination perturbs their electronic structures. The fundamental behavior of the B–S-coordinated compounds upon heating or light irradiation is discussed in this article.

## Results and discussion

**Scheme 1:**
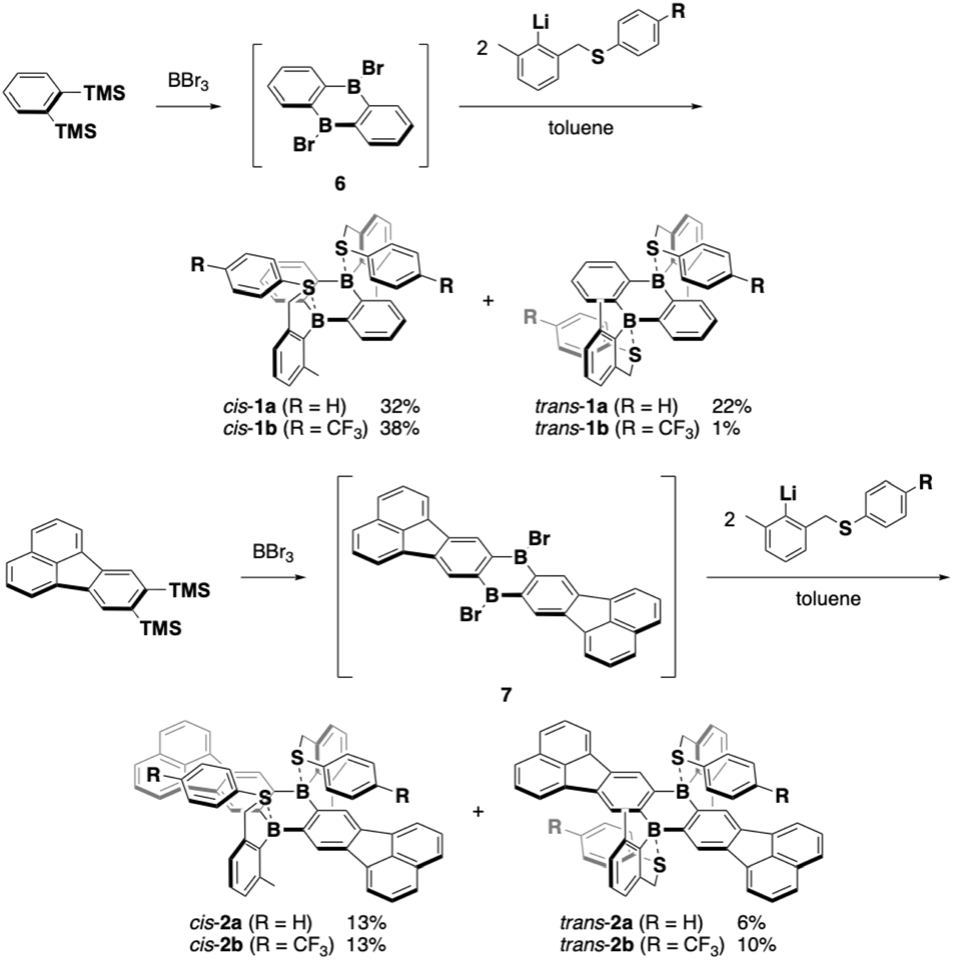
Synthesis of intramolecular arylsulfide-coordinated diboraanthracenes.

Intramolecularly arylsulfide-coordinated DBAs 1 and 2 were synthesized by employing 9,10-dibromo-9,10-dihydro-9,10-diboraanthracene (6) and its π-extended analogue 7 as key precursors, respectively (Scheme 1).^23,27^ Thus, compound 6, which was prepared *in situ* according to the literature method,^28^ was treated with 2 equiv. of [2-(phenylthiomethyl)-6-methylphenyl]lithium. This reaction gave a mixture of *cis* and *trans* isomers of 1a, which could be separated by silica gel column chromatography. CF_3_-substituted derivative 1b and π-extended analogues 2a and 2b were prepared in a similar manner, and all *cis* and *trans* isomers were successfully separated by silica gel column chromatography or HPLC on silica gel. In both scaffolds, the *cis* isomers were predominantly obtained. These compounds thus obtained were sufficiently stable to be handled under ambient conditions without special precautions. In particular, the *cis* and *trans* isomers showcased sufficient configurational stability at room temperature, whereas *cis*–*trans* isomerization proceeded at higher temperatures (*vide infra*).

**Fig. 2:**
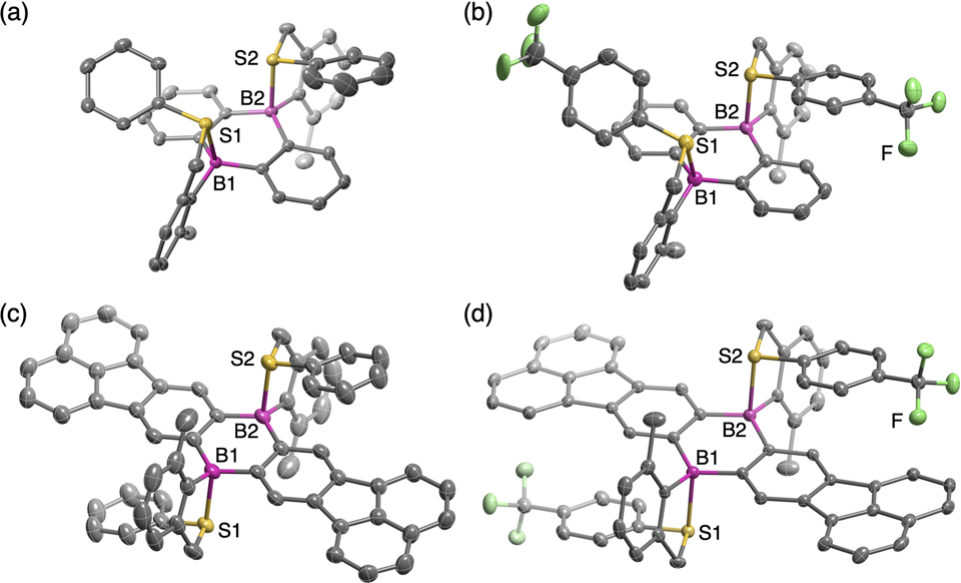
Crystal structures of (a) *cis*-1a, (b) *cis*-1b, (c) *trans*-2a, and (d) *trans*-2b with thermal ellipsoids at 50% probability. Only one of the two crystallographically independent molecules of *cis*-1b is shown. Hydrogen atoms and solvent molecules are omitted for clarity.

The structures of some of the compounds were unequivocally determined by single-crystal X-ray diffraction analysis. Crystal structures of *cis*-1a, *cis*-1b, *trans*-2a, and *trans*-2b are shown in Fig. 2, which clearly showcases that the coordination of the sulfur atoms to the boron atoms formed 5-membered rings irrespective of their *cis*- or *trans*-configurations. The central dibora-hexagon rings in the DBA skeletons of *cis*-1a and *cis*-1b adopted slightly distorted boat-like conformations, while *trans*-2a and *trans*-2b retained planar structures. The B–S distances in these compounds (*cis*-1a: 2.161(1) Å, *cis*-1b: 2.146(3)–2.381(3) Å, *trans*-2a: 2.228(4) Å, and *trans*-2b: 2.289(2) Å, Table 1) are much shorter than the sum (3.72 Å) of the van der Waals radii of the boron and sulfur atoms.^29^ The B–S distances are slightly longer compared to those of hitherto-known sulfur-coordinate compounds such as A (2.029(1) Å),^17^B (2.104(6) Å),^20*d*^ and C (2.031(2) Å),^26^ suggesting that the B–S interaction in 1 and 2 are rather weak. The slightly longer B–S distances observed for the CF_3_-substituted derivatives *cis*-1b and *trans*-2b relative to *cis*-1a and *trans*-2a, respectively, demonstrated that the B–S interaction is weakened by decreasing the Lewis basicity of the sulfur atom. As a result of the coordination, the boron atoms adopted tetrahedral geometries with the sum of the bond angles around the boron atoms of 350.2° for *cis*-1a, 350.6–354.4° for *cis*-1b, 353.5° for *trans*-2a, and 355.1° for *trans*-2b. The tetrahedral characters (THCs)^30^ of their boron centers were calculated to be 31.1% for *cis*-1a, 17.8–29.8% for *cis*-1b, 20.7% for *trans*-2a, and 15.6% for *trans*-2b. It should be also noted that the B–S coordination resulted in the face-to-face orientation of the arylthio group against the DBA skeleton with the interfacial distances of 3.05–3.45 Å, although the overlaps between the π-planes were too small to form strong π–π interactions.

**Table 1:** Selected bond lengths (Å) and angles (°), and tetrahedral characters (THC) (%)

Compound		B–S bond lengths/Å		Σ(C–B–C)/°	THC/%
*cis*-1a		B1–S1	2.161(1)	350.2	31.1
		B2–S2	2.161(1)	350.2	31.1
*cis*-1b^a^	A	B1–S1	2.181(2)	350.6	29.8
		B2–S2	2.231(3)	353.4	21.0
	B	B1–S1	2.146(3)	351.0	28.6
		B2–S2	2.381(3)	354.4	17.8
*trans*-2a		B1–S1/B2–S2	2.228(4)	353.5	20.7
*trans*-2b		B1–S1/B2–S2	2.289(2)	355.1	15.6

a *cis*-1b contains two crystallographically independent molecules A and B in the unit cell.

To gain insight into the intramolecular B–S coordination bond, natural bond orbital (NBO) analyses were conducted at the B3LYP-D3/6-311+G(d,p) level of theory on *cis*-1a, *cis*-1b, *trans*-1a, and *trans*-1b using their optimized structures obtained at the PBE0/6-31G(d) level.^31^ For comparison, NBO analyses were also conducted for methylsulfide-coordinated borafluorene A and DBA *cis*-5 (Fig. 1) as model compounds. The obtained Wiberg Bond Index (WBI) values are summarized in Table 2. In comparison with the WBI value of A, derived from antiaromatic and highly Lewis acidic borafluorene, those of the DBA compounds were rather small, indicating that their B–S coordination bonds are weak. A comparison between *cis*-1a, *cis*-1b, and *cis*-5 with different substituents on the sulfur atom demonstrated that the WBI values decreased in the order of *cis*-5 > *cis*-1a > *cis*-1b, indicating that the arylsulfide group renders the B–S coordination bond more labile. Moreover, the smaller WBI values of *trans*-1a and *trans*-1b compared with those of *cis*-1a and *cis*-1b, respectively, indicate that the B–S coordination bonds in the *trans*-isomers are weaker, suggesting that the configuration also affects the strength of the B–S coordination bonds. Although the origin of this difference remains unclear, it might be related to the fact that the *trans* isomers retain the planar conformation of the DBA moiety, whereas the *cis* isomers adopt a bent conformation deviated from the planar structures, which most likely decreases the steric congestion (Fig. S6†).

**Table 2:** B–S bond lengths (Å) and Wiberg bond index values for optimized structures of sulfide-coordinated compounds^a^

Compound	B–S bond lengths/Å		Wiberg bond index
*cis*-1a	B1–S1	2.233	0.537
	B2–S2	2.232	0.539
*cis*-1b	B1–S1	2.296	0.484
	B2–S2	2.296	0.484
*trans*-1a	B1–S1	2.284	0.504
	B2–S2	2.285	0.503
*trans*-1b	B1–S1	2.382	0.429
	B2–S2	2.347	0.452
*cis*-5	B1–S1	2.145	0.625
	B2–S2	2.145	0.625
A	B1–S1	2.053	0.714

a NBO calculations were conducted at the B3LYP-D3/6-311+G(d,p) level of theory.

The intramolecular B–S coordination was also observed in solution *via*^11^B NMR spectroscopy (Fig. S1†). Thus, the ^11^B NMR spectra of 1 and 2 in CDCl_3_ showed relatively sharp signals at around 20–40 ppm at room temperature. These results contrast with the broad signals observed at around 70 ppm for the tricoordinate congeners 3 and 4, indicating that 1 and 2 adopt tetracoordinate structures even in solution. However, the chemical shifts of 1 and 2 appeared in a relatively low magnetic field region for tetracoordinate boron species (for instance, 5.3 ppm in CDCl_3_ for A), which suggests a relative weakness of the B–S coordination bonds in 1 and 2. For derivatives 1 and 2, the *trans* configuration and the introduction of an electron-withdrawing CF_3_ group shifted the signals to a lower magnetic field, implying that the strength of the B–S coordination was further perturbed to some extent due to these structural and electronic modifications.

As a consequence of the weak bonding character of the B–S coordination, the DBA derivatives underwent thermal *cis*–*trans* isomerization. For example, *cis*-1a isomerized to form some of the corresponding *trans* isomer upon increasing the temperature in solution (Fig. S2†). Using the intensity ratios of their ^1^H NMR spectra in toluene-*d*_8_, the equilibrium constants (*K*_eq_) for the *cis*–*trans* isomerization of *cis*-1a and *cis*-1b at 363 K were determined to be 0.28 and 0.48, respectively (Table S1†). Similar NMR measurements were conducted at various temperatures to determine the thermodynamic parameters for the *cis*–*trans* isomerization of *cis*-1a and *cis*-1b. Based on the corresponding van't Hoff plots (Fig. S3†), enthalpy changes (Δ*H*) of 11.8 and 8.4 kJ mol^−1^ and entropy changes (Δ*S*) of 21.9 and 16.9 J mol^−1^ K^−1^ were determined for *cis*-1a and *cis*-1b, respectively (Table S2†). Using these values, the Gibbs free energy changes for the *cis* → *trans* conversions of *cis*-1a and *cis*-1b at 298 K were calculated to be 5.30 and 3.32 kJ mol^−1^, respectively, demonstrating that the isomerization from the *cis* isomer to the *trans* isomer was slightly endergonic. Furthermore, the introduction of an electron-withdrawing CF_3_ group to the phenyl group on the sulfur atom reduced the energy difference between the *cis* and *trans* isomers.

**Fig. 3:**
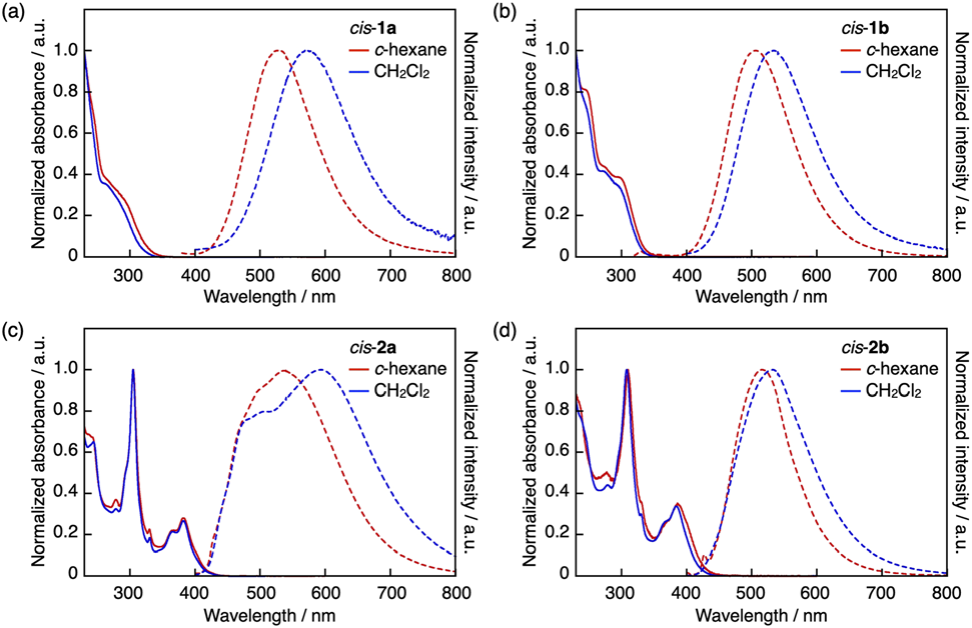
UV-vis absorption (solid lines) and fluorescence (dashed lines) spectra of (a) *cis*-1a, (b) *cis*-1b, (c) *cis*-2a, and (d) *cis*-2b in cyclohexane (red) and CH_2_Cl_2_ (blue).

The intramolecular B–S coordination affects considerably the photophysical properties of the DBAs. Thus, the UV-vis absorption spectra of *cis*-1a and *cis*-1b in cyclohexane showed absorption bands with a maximum wavelength (*λ*_abs_) of around 290 nm (Fig. 3a and b), which were blue-shifted compared with that of mesityl-substituted DBA 3 (*λ*_abs_ = 406 nm).^24*a*^ Similar blue shifts were also observed for π-expanded analogues *cis*-2a and *cis*-2b (*cis*-2a: *λ*_abs_ = 382 nm; *cis*-2b: *λ*_abs_ = 386 nm in cyclohexane; Fig. 3c and d) compared with that of 4 (*λ*_abs_ = 435 nm).^23^ These shifts can be attributed to the disruption of the p–π* conjugation through the vacant p orbital of the boron atom due to the coordination of the sulfur atom.

In stark contrast, in the fluorescence spectra, the B–S coordination gave rise to red shifts in the emission maxima. Thus, in cyclohexane, *cis*-1a and *cis*-1b showed substantially red-shifted emissions with maximum wavelengths (*λ*_em_) of 526 and 506 nm, respectively (Fig. 3a and b), while their fluorescence quantum yields (*Φ*_F_) were low (0.02–0.03). Notably, their *λ*_em_ values were more than or nearly 100 nm longer compared with that of tricoordinate compound 3 (*λ*_em_ = 413 nm).^24*a*^ As a consequence, *cis*-1a and *cis*-1b exhibited considerably large apparent Stokes shifts (Δ*ν* = *ν*_abs_ − *ν*_em_) of 16 500 and 13 800 cm^−1^, respectively, even in nonpolar cyclohexane. The emission bands of *cis*-1a and *cis*-1b were further shifted to 572 and 533 nm, respectively, in CH_2_Cl_2_, resulting in even larger apparent Stokes shifts of 18 600 and 15 400 cm^−1^. Several boron-based fluorophores with a weakly coordinated Lewis base are known to undergo photodissociation of the boron–Lewis base coordination bond in the excited state, resulting in an emission from the tricoordinate species.^15–17,20*d*^ However, the red-shifted emission of *cis*-1a and *cis*-1b relative to 3 cannot be explained by simply considering the photodissociation behavior. The broad shape of the emission bands and the dependence of the emission wavelength on solvent polarity suggest that intramolecular charge transfer (ICT) character in the excited state is likely responsible for these red-shifted emissions.

Meanwhile, *cis*-2a and *cis*-2b showed weak broad emission bands with *λ*_em_ at 540 and 516 nm in cyclohexane (Fig. 3c and d), respectively, which were comparable to that of tricoordinate congener 4 (*λ*_em_ = 520 nm),^23^ while the fluorescence quantum yields of *cis*-2a (0.02) and *cis*-2b (0.04) were lower than that of 4 (0.26). In CH_2_Cl_2_, *cis*-2a showed broad emission bands likely consisting of two bands at around 500 and 600 nm, while the emission band of *cis*-2b (*λ*_em_ = 532 nm) was only slightly red-shifted compared to that in cyclohexane. These results indicate that while the π-expanded DBA skeleton also undergoes photodissociation, the ICT transition character is not always involved. Instead, it depends on the electronic effect of the aryl group on the sulfur atom.

The difference in the *cis*/*trans* configuration also affected the photophysical properties of the arylsulfide-coordinated DBAs. Thus, in the UV-vis absorption spectra in cyclohexane (Fig. S4†), *trans*-1a showed a slightly red-shifted absorption band relative to that of *cis*-1a (*trans*-1a: *λ*_abs_ = 300 nm; *cis*-1a: *λ*_abs_ = 282 nm). In the emission spectrum in cyclohexane, the emission band of *trans*-1a was slightly blue-shifted with a decreased apparent Stokes shift compared to *cis*-1a (*trans*-1a: *λ*_em_ = 512 nm, Δ*ν* = 13 800 cm^−1^; *cis*-1a: *λ*_em_ = 526 nm, Δ*ν* = 16 500 cm^−1^). Thus, the large apparent Stokes shift observed for *cis*-1a likely results partly from the *cis*-configuration structure. A similar trend was observed for other derivatives except for 1b (Table S3†).

**Fig. 4:**
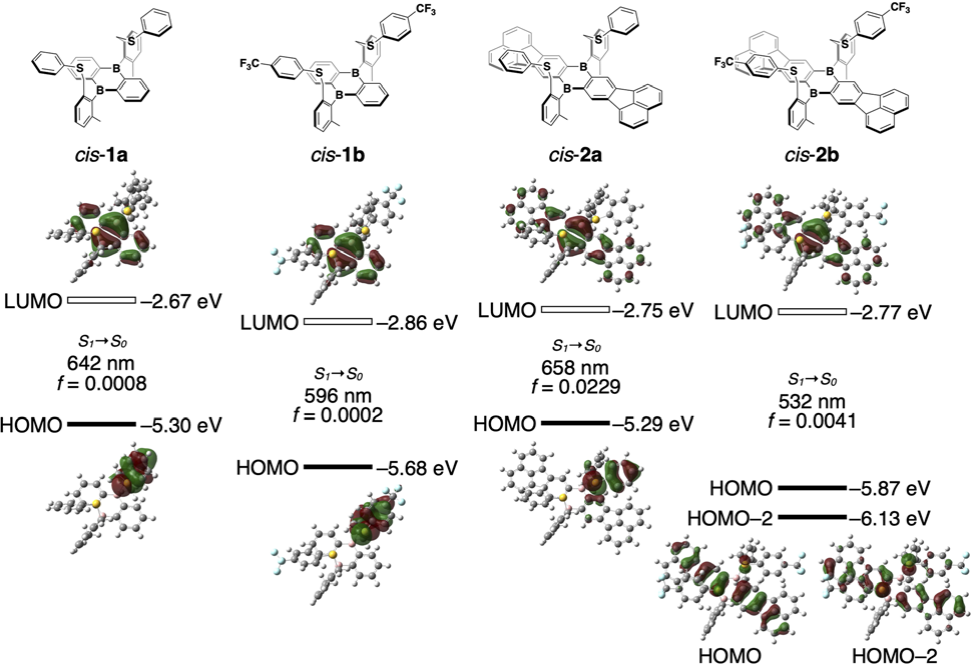
Kohn–Sham molecular orbitals for *cis*-1a, *cis*-1b, *cis*-2a, and *cis*-2b in the S_1_ optimized structures. TD-DFT calculations were carried out at the PBE0/6-31G(d) level of theory.

To gain more insight into the photodissociation process, time-dependent density functional theory (TD DFT) calculations were conducted on 1 and 2 at the PBE0/6-31G(d) level of theory. The structural optimization in *cis*-1a in the lowest excited singlet state (S_1_) only gave a tricoordinate structure where both B–S bonds are dissociated, even when starting the optimization from an initial B–S-coordinated structure (Fig. 4). Similar results were obtained for *cis*-1b, *cis*-2a, and *cis*-2b. In the optimized structure of *cis*-1a in S_1_, the DBA skeleton became planar and the phenylthio groups were displaced from the DBA skeleton compared with the optimized structure in S_0_. While the optimized structure of *cis*-1b in S_1_ was similar to that of *cis*-1a, the phenylthio groups in *cis*-2a and *cis*-2b were oriented in closer proximity to the DBA skeletons in their optimized structures in S_1_ (B⋯S distances in S_1_: *cis*-1a, 3.54, 3.56 Å; *cis*-2a, 3.42, 3.46; *cis*-2b, 3.25, 3.25 Å). Notably, in the structures of *cis*-1a, *cis*-1b, and *cis*-2a in S_1_, the HOMOs were localized on the phenylthio groups and the LUMOs on the DBA skeletons. In contrast, both HOMO and LUMO of CF_3_-substituted *cis*-2b were localized on the π-expanded DBA skeleton, indicating that the emission of *cis*-2b has mainly a π–π* transition character, which is consistent with the fact that the emission band of *cis*-2b did not show solvent polarity dependence.

In the structure optimized in S_1_, *cis*-1a has a rather high-lying HOMO of −5.30 eV despite the fact that it is mainly localized in the phenylthio moiety. In this structure, the bond length between the sulfur atom and the *ipso*-carbon atom of the phenyl group was shorter compared to that in S_0_ (Fig. S9†). In addition, a larger bond-length alternation was observed in the phenyl moiety in the S_1_ optimized structure, indicating that the sulfur atom donates a lone pair electron to the phenyl group upon photodissociation.

Although both the HOMO and HOMO−1 of *cis*-1a were localized on different phenylthio moieties, the HOMO level was higher by 0.77 eV than the HOMO−1 level (Fig. S10†). For comparison, the TD-DFT calculation was also conducted for phenylmethylsulfide. The HOMO level in the S_1_ optimized structure was estimated to be −6.08 eV, which was comparable to the HOMO−1 of *cis*-1a. While the lone pair orbital of the sulfur atom and π-orbital of the phenyl group were almost orthogonal in the HOMO−1 of *cis*-1a, these orbitals were parallel in the HOMO of *cis*-1a, which most likely contributes to the high-lying HOMO localized in the phenylthio moiety of *cis*-1a. In contrast, such a high energy level of the arylthio moiety was not observed in *cis*-2b, suggesting that the energy-level balance between the DBA skeleton and the arylthio moiety is crucial for the unusual emission properties observed in the phenylthio-substituted derivatives.

**Fig. 5:**
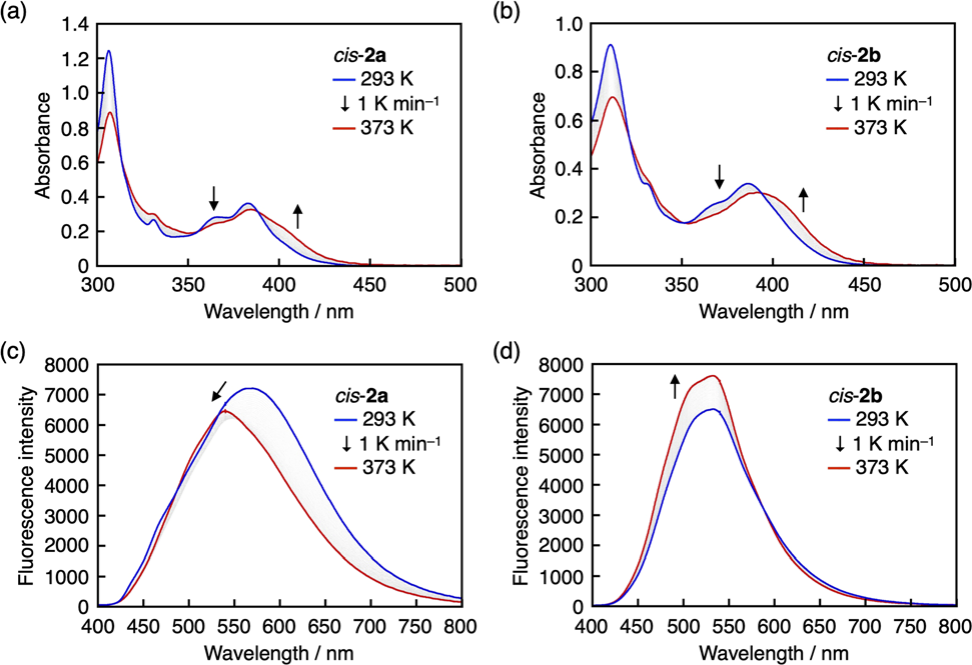
(a and b) UV-vis absorption and (c and d) fluorescence spectra of (a and c) *cis*-2a and (b and d) *cis*-2b upon heating from 293 to 373 K at 1 K min^−1^ in toluene.

As mentioned above, the *cis*–*trans* isomerization of arylsulfide-coordinated DBAs occurred in toluene-*d*_6_ upon heating, suggesting that the B–S bond dissociates at high temperatures. This behavior was confirmed *via* temperature-dependent UV-vis absorption and fluorescence measurements, which were performed for *cis*-2a and *cis*-2b because *cis*-1a and *cis*-1b lacked absorption bands in the visible region. Upon heating toluene solutions of *cis*-2a and *cis*-2b from 293 to 373 K, bathochromic shifts of the absorption bands were observed with isosbestic points (Fig. 5a and b). Since the absorption spectra of *cis*-2 and *trans*-2 are nearly identical (Fig. S4c and d†), these changes are unlikely to arise solely from *cis*–*trans* isomerization. We therefore attribute them to partial dissociation of the B–S coordination bond in the ground state. In the fluorescence spectra, the fluorescence intensity of *cis*-2a decreased with increasing temperature, which was accompanied by a hypsochromic shift of the emission maximum wavelength (Fig. 5c). In contrast to *cis*-2a, the fluorescence intensity of *cis*-2b was enhanced with increasing temperature (Fig. 5d). It should be noted that these spectral changes were reversible: the original spectra of both compounds were observed upon cooling from 373 to 293 K (Fig. S5†).

**Fig. 6:**
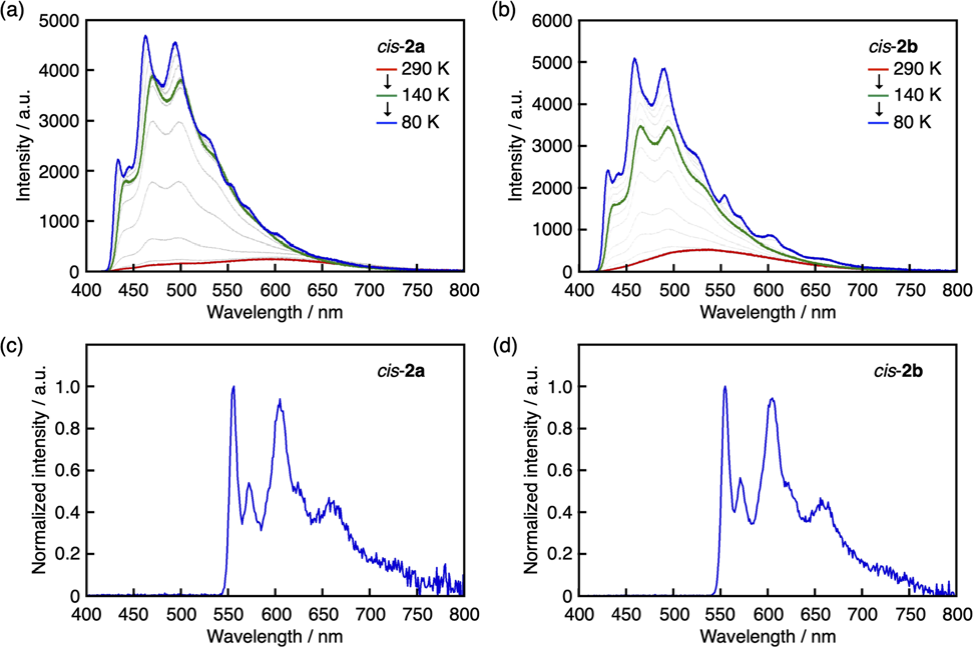
Temperature-dependent fluorescence spectra of (a) *cis*-2a and (b) *cis*-2b in 2-MeTHF. Phosphorescence spectra of (c) *cis*-2a and (d) *cis*-2b in 2-MeTHF at 77 K with a delay time of 50 ms.

In both π-expanded *cis*-2a and *cis*-2b in 2-MeTHF, when the temperature was decreased from 290 to 80 K, vibronically structured emission bands with increased intensity appeared at around 420–550 nm (Fig. 6a and b). These emission bands likely stem from tetracoordinate species in which the sulfur atoms remain coordinated to the boron atoms. The fact that these new bands appeared above the glass transition temperature of the solvent suggests that the decrease in temperature instead of the increased viscosity of the medium is responsible for the retardation of the B–S dissociation in the excited state. At temperatures lower than the glass transition temperature of 2-MeTHF (137 K), a new vibronically structured band was observed at around 550–700 nm, which is assignable to a phosphorescence band because it matched with an emission spectrum measured with a delay time of 50 ms (Fig. 6c and d). Ultimately, *cis*-2a and *cis*-2b showed photoluminescence quantum yields, including fluorescence and phosphorescence, of 0.40 and 0.37, respectively, at 77 K.

## Conclusions

A series of 9,10-dihydro-9,10-diboraanthracenes with arylthiomethyl substituents on the *ortho* positions of the phenyl groups on the boron atom were synthesized as a new class of intramolecularly sulfur-coordinated boron-containing π-conjugated molecules. The B–S coordination bonds were sufficiently strong to allow the separation of the *cis* and *trans* isomers by silica gel column chromatography. A crystallographic analysis revealed that the strength of the B–S coordination bond was perturbed by introducing an electron-withdrawing CF_3_ group on the arylthio moiety. A NBO analysis complemented the experimental findings suggesting that the CF_3_-containing derivatives have smaller WBI values. The B–S coordination bonds were retained even in solution at room temperature, whereas dissociation partially occurred in the ground state at elevated temperature, as evidenced by their ^1^H NMR spectra. The introduction of the CF_3_ group reduced the enthalpy change in the *cis*–*trans* isomerization. The B–S bonds also dissociated in response to light irradiation, resulting in photodissociation-induced emissions from tricoordinate species with large apparent Stokes shifts. Some derivatives also showcased substantially red-shifted emission bands with ICT transition character. Thus, the arylthio-substituted DBAs exhibited multifaceted emissions from a tetracoordinate species, a tricoordinate species after photodissociation, and a tricoordinate species in the ICT state in S_1_, as well as phosphorescence, depending on the environment. These results demonstrate the potential utility of B–S coordination bonds for the design of unprecedented multiply emissive materials.

## Data availability

The data supporting this article have been included as part of the ESI.†

## Author contributions

H. N., M. W. and S. Y. conceived the idea. H. N. synthesized the compounds and evaluated their properties with the support of H.-W. L., H. N. performed the X-ray crystal structure analyses of *cis*-1a, *cis*-1b, and *trans*-2b. A. V. performed the X-ray crystal structure analysis of *trans*-2a. H. N. and S. Y. wrote the manuscript, and all authors discussed and commented on the manuscript. M. W. and S. Y. directed the project.

## Conflicts of interest

There are no conflicts to declare.

## Supplementary Material

SC-OLF-D5SC01726B-s001

SC-OLF-D5SC01726B-s002
