# Supplementary material for: Intramolecular arylsulfide-coordinated diboraanthracenes: effect of B–S coordination on ground-state and excited-state behavior
Source: Chem Sci. 2025 Apr 14;16(20):8764–71. doi: 10.1039/d5sc01726b (PMC12012721; doi:10.1039/d5sc01726b)
Supplement: SC-016-D5SC01726B-s001 [file SC-016-D5SC01726B-s001.pdf]

Supplementary Information (SI) for Chemical Science.
This journal is © The Royal Society of Chemistry 2025

Supporting Information

Intramolecular Arylsulfide-Coordinated Diboraanthracenes: Effect of B–S Coordination on Ground-State and Excited-State Behavior

Hiroki Narita, Alexander Virovets, Hans-Wolfram Lerner, Matthias Wagner and Shigehiro Yamaguchi*

Contents

1. Experimental Details S2
2. X-ray Crystallographic Analysis S8
3. Analysis of B–S Interaction S11
4. Thermal *cis–trans* Isomerization S12
5. Photophysical Properties S14
6. Theoretical Study S17
7. References S37
8. NMR Spectra S39

1. Experimental Section

General Procedures. Melting points (Mp) were determined by a Yanaco MP-S3 instrument (MP-S3). ^{1}H, ^{11}B{^{1}H}, and ^{19}F NMR spectra were recorded with a JEOL JNM-ECS-400 spectrometer (400 MHz for ^{1}H, 128 MHz for ^{11}B{^{1}H}, and 376 MHz for ^{19}F) in $CDCl_3$, CD_2Cl_2, or acetone-d_6. ^{13}C{^{1}H} NMR spectra were recorded with a JEOL ECA 600II with an Ultra COOLTM probe (151 MHz for ^{13}C{^{1}H}) in $CDCl_3$. The chemical shifts in ^{1}H NMR spectra are reported in δ ppm using the residual protons of the solvents, $CHCl_3$ (δ 7.26) in $CDCl_3$, CH_2Cl_2 (δ 5.32) in CD_2Cl_2, or $(CH_3)_2CO$ (δ 2.05) in acetone-d_6 as an internal standard and those in ^{13}C{^{1}H} NMR spectra are reported using the solvent signals of $CDCl_3$ (δ 77.16) as an internal standard. The chemical shifts in ^{11}B{^{1}H} NMR spectra are reported using $BF_3{\cdot}OEt_2$ (δ 0.00) as an external standard. The chemical shifts in ^{19}F NMR spectra are reported using CF_3COOH (δ –78.50) as an external standard. Mass spectra were measured with a Bruker micrOTOF Focus spectrometer with the APCI ionization method using toluene. Thin layer chromatography (TLC) was performed on glass coated with 0.25 mm thickness of silica gel 60 F_{254} (Merk). Column chromatography was performed using silica gel PSQ60B (Fuji Silysia Chemical). Preparative high performance liquid chromatography (HPLC) was performed using LaboACE LC-5060 equipped with a silica gel column (JAIGEL-SIL-L, Japan Analytical Industry). Anhydrous THF and toluene were purchased from FUJIFILM Wako Chemicals, and further purified by Glass Contour Solvent Systems. 2-Bromo-1-(bromomethyl)-3-methylbenzene,[S1] 9,10-dibromo-9,10-dihydro-9,10-diboraanthracene,[S2] and bisacenaphtheno[1,2-*b*;1′,2′-*i*](9,10-dibromo-9,10-dihydro-9,10-diboraanthracene)[S3] were prepared according to the literature methods. All reactions were performed with dry glassware and under a nitrogen atmosphere.

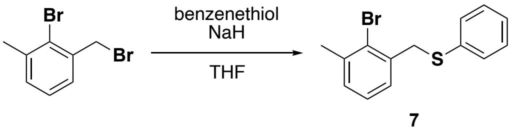

2-Bromo-1-methyl-3-(phenylsulfanylmethyl)benzene (7). To a solution of benzenethiol (2.42 g, 22.0 mmol) in THF (50 mL) was added NaH (60% in paraffin liquid, 880 mg, 22.0 mmol) at room temperature. After stirring at room temperature for 1 h, 2-bromo-1-(bromomethyl)-3-methylbenzene (5.28 g, 20.0 mmol) was added to the mixture

followed by stirring at 80 °C for 3 h. After cooling to room temperature, a saturated NH_4Cl aqueous solution was added to the reaction mixture. The organic layer was separated and the aqueous layer was extracted with CH_2Cl_2. The combined organic layer was washed with brine, dried over Na_2SO_4, filtered, and concentrated under reduced pressure. The mixture was subjected to silica gel column chromatography (hexane, $R_f = 0.21$) to give 5.59 g (19.1 mmol, 95%) of **7** as a colorless solid: Mp. 36.9–37.6 °C; ^{1}H NMR (400 MHz, acetone-d_6) δ 7.39–7.37 (m, 2H), 7.31 (td, J = 6.5, 1.7 Hz, 2H), 7.25–7.14 (m, 4H), 4.33 (s, 2H), 2.41 (s, 3H); ^{13}C{^{1}H} NMR (100 MHz, $CDCl_3$) δ 139.1, 137.2, 136.2, 130.6, 129.9, 129.0, 128.2, 127.2, 126.9, 126.7, 40.7, 24.1; HRMS (EI) *m*/*z* calcd for $C_{14}H_{13}BrS$ [*M*]$^+$ 291.9916, found: 291.9911.

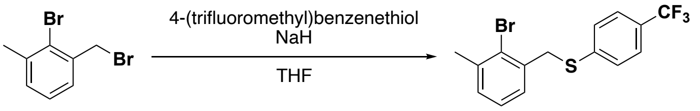

2-Bromo-1-methyl-3-[(4-trifluoromethylphenyl)sulfanylmethyl]benzene (8). To a solution of 4-(trifluoromethyl)benzenethiol (3.92 g, 22.0 mmol) in THF (50 mL) was added NaH (60% in paraffin liquid, 880 mg, 22.0 mmol) at room temperature. After stirring at room temperature for 1 h, 2-bromo-1-(bromomethyl)-3-methylbenzene (5.28 g, 20.0 mmol) was added to the mixture followed by stirring at 80 °C for 3 h. After cooling to room temperature, a saturated NH_4Cl aqueous solution was added to the reaction mixture. The organic layer was separated and the aqueous layer was extracted with CH_2Cl_2. The combined organic layer was washed with brine, dried over Na_2SO_4, filtered, and concentrated under reduced pressure. The mixture was subjected to silica gel column chromatography (hexane, $R_f = 0.33$) to give 6.60 g (18.3 mmol, 92%) of **8** as a colorless solid: Mp. 93.8–94.3 °C; ^{1}H NMR (400 MHz, acetone-d_6) δ 7.64 (d, J = 8.7 Hz, 2H), 7.56 (d, J = 8.7 Hz, 2H), 7.34 (dd, J = 7.3, 1.8 Hz, 1H), 7.27 (dd, J = 7.3, 1.8 Hz, 1H), 7.21 (t, J = 7.5 Hz, 1H), 4.47 (s, 2H), 2.43 (s, 3H); ^{13}C{^{1}H} NMR (100 MHz, $CDCl_3$) δ 141.9, 139.3, 136.1, 130.2, 128.6, 128.09, 128.07 (J = 32.8 Hz), 127.3, 127.1, 125.8 (J = 2.9 Hz), 124.2 (J = 272 Hz), 39.3, 24.1; ^{19}F NMR (376 MHz, $CDCl_3$) δ –63.5; HRMS (EI) *m*/*z* calcd for $C_{15}H_{12}BrF_3S$ [*M*]$^+$ 359.9790, found: 359.9792.

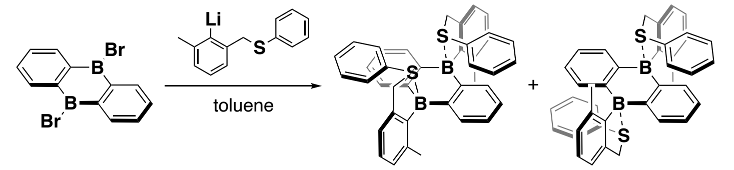

Compound 1a. To a solution of **7** (293 mg, 1.00 mmol) in toluene (5 mL) was added a cyclohexane solution of *s*-BuLi (1.3 M, 0.769 mL, 1.00 mmol) dropwise at 0 °C. After stirring at the same temperature for 1 h, the reaction mixture was added to a suspension of 9,10-dibromo-9,10-dihydro-diboraanthracene (167 mg, 0.500 mmol) in toluene (5 mL) at 0 °C. The mixture was allowed to warm to room temperature followed by stirring for 20 h. After addition of MeOH (5 mL), all volatiles were removed under reduced pressure. The mixture was subjected to silica gel column chromatography (7/3 hexane/CH_2Cl_2, R_f = 0.19 and 0.21 for *cis* and *trans* isomers, respectively) to give 96.5 mg (0.161 mmol, 32%) of *cis*-**1a** and 67.2 mg (0.112 mmol, 22%) of *trans*-**1a** as colorless solids.

cis-**1a**: Mp. 210.6–211.4 °C; ^{1}H NMR (400 MHz, CD_2Cl_2) δ 7.36 (d, J = 7.3 Hz, 2H), 7.28 (t, J = 7.5 Hz, 2H), 7.17–7.13 (m, 2H), 7.10–7.03 (m, 6H), 6.92 (d, J = 8.7 Hz, 4H), 6.76–6.69 (m, 8H), 4.59 (s, 4H), 1.72 (s, 6H); ^{13}C{^{1}H} NMR (151 MHz, $CDCl_3$) δ 149.4, 147.6, 142.4, 142.3, 134.1, 132.6, 129.2, 128.8, 127.8, 127.6, 126.73, 126.66, 121.7, 41.5, 22.7; ^{11}B{^{1}H} NMR (128 MHz, $CDCl_3$) δ 24.5; HRMS (APCI) *m/z* calcd for $C_{40}H_{34}B_2S_2$ $[M]^+$ 600.2283, found: 600.2296.

trans-**1a**: Mp. 183.0–183.9 °C; ^{1}H NMR (400 MHz, CD_2Cl_2) δ 7.35 (d, J = 7.3 Hz, 2H), 7.29 (t, J = 7.3 Hz, 2H), 7.13 (d, J = 6.9 Hz, 2H), 7.08 (t, J = 7.3 Hz, 2H), 6.97 (t, J = 7.1 Hz, 4H), 6.83–6.81 (m, 4H), 6.75–6.72 (m, 4H), 6.60 (d, J = 7.8 Hz, 4H), 4.49 (s, 4H), 1.99 (s, 6H); ^{13}C{^{1}H} NMR (151 MHz, $CDCl_3$) δ 148.7, 146.9, 142.6, 141.2, 134.2, 132.8, 128.9, 128.7, 127.5, 127.2, 127.1, 126.9, 122.4, 40.4, 23.2; ^{11}B{^{1}H} NMR (128 MHz, $CDCl_3$) δ 30.4; HRMS (APCI) *m/z* calcd for $C_{40}H_{34}B_2S_2$ $[M]^+$ 600.2283, found: 600.2267.

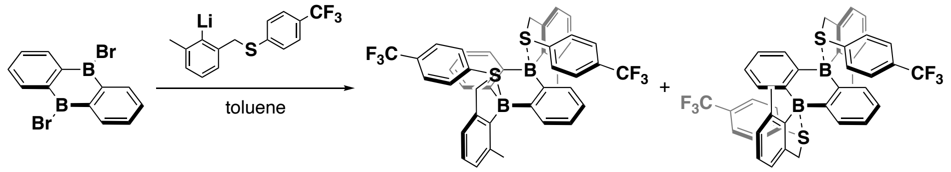

5 *cis*-**1b** *trans*-**1b**

Compound 1b. To a solution of **8** (361 mg, 1.00 mmol) in toluene (5 mL) was added a cyclohexane solution of *s*-BuLi (1.3 M, 0.769 mL, 1.00 mmol) dropwise at 0 °C. After stirring at the same temperature for 1 h, the reaction mixture was added to a suspension of 9,10-dibromo-9,10-dihydro-diboraanthracene (167 mg, 0.500 mmol) in toluene (5 mL) at 0 °C. The mixture was allowed to warm to room temperature followed by stirring for 20 h. After addition of MeOH (5 mL), all volatiles were removed under reduced pressure. The mixture was subjected to silica gel column chromatography (8/2 hexane/CH_2Cl_2, R_f = 0.19) followed by HPLC (8/2 hexane/$CHCl_3$) to give 141.0 mg (0.191 mmol, 38%) of *cis*-**1b** and 4.2 mg (5.7 μmol, 1%) of *trans*-**1b** as colorless solids.

cis-**1b**: Mp. 203.3–204.0 °C; ^{1}H NMR (400 MHz, CD_2Cl_2) δ 7.40 (d, J = 7.8 Hz, 2H), 7.33–7.26 (m, 6H), 7.11 (d, J = 7.3 Hz, 2H), 6.96 (d, J = 8.2 Hz, 4H), 6.77–6.73 (m, 8H), 4.63 (s, 4H), 1.73 (s, 6H); ^{13}C{^{1}H} NMR (151 MHz, $CDCl_3$) δ 147.7, 146.9, 142.1, 141.4, 137.6, 134.4, 129.53 (J = 32.8 Hz), 129.50, 127.6, 127.2, 127.1, 125.6 (J = 4.4 Hz), 123.9 (J = 272 Hz), 122.2, 40.2, 22.8; ^{11}B{^{1}H} NMR (128 MHz, $CDCl_3$) δ 30.7; ^{19}F NMR (376 MHz, $CDCl_3$) δ –63.8; HRMS (APCI) *m/z* calcd for $C_{42}H_{32}B_2F_6S_2$ $[M]^+$ 736.2030, found: 736.2034.

trans-**1b**: Mp. 200.5–201.3 °C; ^{1}H NMR (400 MHz, $CDCl_3$) δ 7.36–7.31 (m, 4H), 7.18–7.15 (m, 6H), 6.87–6.84 (m, 4H), 6.80–6.77 (m, 4H), 6.61 (d, J = 8.2 Hz, 4H), 4.44 (s, 4H), 2.05 (s, 6H); ^{13}C{^{1}H} NMR (151 MHz, $CDCl_3$) δ 147.1, 146.3, 141.9, 140.9, 137.9, 134.4, 129.2, 129.0 (J = 33.2 Hz), 128.3, 127.3, 126.6, 125.6 (J = 2.9 Hz), 124.0 (J = 272 Hz), 122.9, 39.3, 23.2; ^{11}B{^{1}H} NMR (128 MHz, $CDCl_3$) δ 35.4; ^{19}F NMR (376 MHz, $CDCl_3$) δ –63.7; HRMS (APCI) *m/z* calcd for $C_{42}H_{32}B_2F_6S_2$ $[M]^+$ 736.2030, found: 736.2015.

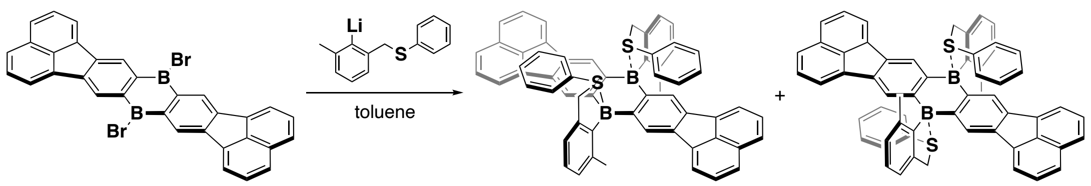

Compound 2a. To a solution of **7** (117 mg, 0.400 mmol) in toluene (5 mL) was added a cyclohexane solution of *s*-BuLi (1.4 M, 0.286 mL, 0.400 mmol) dropwise at 0 °C. After stirring at the same temperature for 1 h, the reaction mixture was added to a suspension of bisacenaphtheno[1,2-*b*;1′,2′-*i*](9,10-dibromo-9,10-dihydro-diboraanthracene) (116 mg, 0.200 mmol) in toluene (5 mL) at 0 °C. The mixture was allowed to warm to room temperature followed by stirring for 20 h. After addition of MeOH (5 mL), all volatiles were removed under reduced pressure. The mixture was subjected to silica gel column chromatography (6/4 hexane/CH_2Cl_2, R_f = 0.31 and 0.33 for *cis* and *trans* isomers, respectively) to give 21.5 mg (25.3 μmol, 13%) of *cis*-**2a** and 9.3 mg (10.9 μmol, 6%) of *trans*-**2a** as yellow solids.

cis-**2a**: Mp. 271.9–272.8 °C; ^{1}H NMR (400 MHz, $CDCl_3$) δ 7.69 (d, J = 8.2 Hz, 4H), 7.55–7.40 (m, 12H), 7.34 (s, 4H), 7.19 (d, J = 7.3 Hz, 2H), 6.98 (d, J = 8.7 Hz, 4H), 6.84 (t, J = 7.8 Hz, 4H), 6.68 (t, J = 7.3 Hz, 2H), 4.71 (s, 4H), 1.88 (s, 6H); ^{13}C{^{1}H} NMR (151 MHz, $CDCl_3$) δ 149.0, 148.1, 142.5, 142.4, 138.3, 138.1, 132.9, 132.3, 129.9, 129.5, 128.6, 128.0, 127.7, 127.42, 127.37, 127.0, 125.7, 122.0, 119.5, 41.2, 22.8; ^{11}B{^{1}H} NMR (128 MHz, $CDCl_3$) δ 27.6; HRMS (APCI) *m*/*z* calcd for $C_{60}H_{42}B_2S_2$ [M]$^+$ 848.2909, found: 849.2915.

trans-**2a**: Mp. >300 °C; ^{1}H NMR (400 MHz, CD_2Cl_2) δ 7.73 (d, J = 8.2 Hz, 4H), 7.57 (d, J = 6.9 Hz, 4H), 7.53–7.50 (m, 6H), 7.46–7.42 (m, 6H), 7.25 (d, J = 7.8 Hz, 2H), 6.75-6.70 (m, 8H), 6.52–6.48 (m, 2H), 4.64 (s, 4H), 2.14 (s, 6H); ^{13}C{^{1}H} NMR (151 MHz, $CDCl_3$) δ 148.5, 147.3, 142.9, 141.4, 138.7, 138.1, 132.9, 132.4, 129.9, 129.2, 128.5, 127.7, 127.53, 127.49, 127.2, 126.8, 125.9, 122.8, 119.9, 40.2, 23.3; ^{11}B{^{1}H} NMR (128 MHz, $CDCl_3$) δ 32.2; HRMS (APCI) *m*/*z* calcd for $C_{60}H_{42}B_2S_2$ [M]$^+$ 848.2909, found: 849.2914.

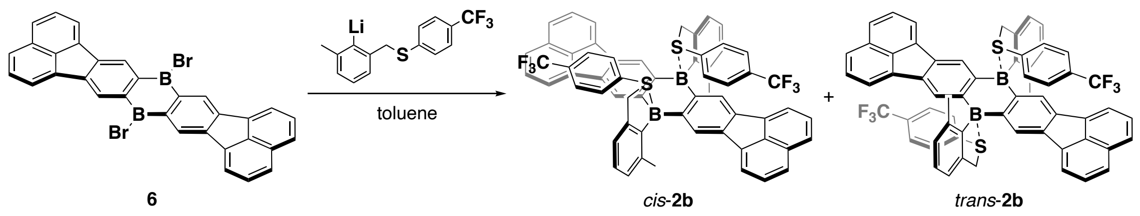

Compound 2b. To a solution of **8** (146 mg, 0.400 mmol) in toluene (5 mL) was added a cyclohexane solution of *s*-BuLi (1.4 M, 0.286 mL, 0.400 mmol) dropwise at 0 °C. After stirring at the same temperature for 1 h, the reaction mixture was added to a suspension of bisacenaphtheno[1,2-*b*;1′,2′-*i*](9,10-dibromo-9,10-dihydro-diboraanthracene) (116 mg, 0.200 mmol) in toluene (5 mL) at 0 °C. The mixture was allowed to warm to room temperature followed by stirring for 20 h. After addition of MeOH (5 mL), all volatiles were removed under reduced pressure. The mixture was subjected to silica gel column chromatography (7/3 hexane/CH_2Cl_2, R_f = 0.24 and 0.26 for *cis* and *trans* isomers, respectively) followed by HPLC (7/3 hexane/$CHCl_3$) to give 25.6 mg (26.0 μmol, 13%) of *cis*-**2b** and 18.9 mg (19.2 μmol, 10%) of *trans*-**2b** as yellow solids.

cis-**2b**: Mp. 226.4–227.2°C; ^{1}H NMR (400 MHz, CD_2Cl_2) δ 7.75 (d, J = 7.8 Hz, 4H), 7.56–7.44 (m, 12H), 7.39 (s, 4H), 7.23 (d, J = 7.3 Hz, 2H), 7.08–7.03 (m, 8H), 4.76 (s, 4H), 1.91 (s, 6H); ^{13}C{^{1}H} NMR (151 MHz, $CDCl_3$) δ 147.4, 147.2, 142.1, 141.5, 139.0, 137.6, 137.4, 132.9, 130.0, 129.8, 129.4 (J = 33.2 Hz), 127.78, 127.75, 127.5, 127.0, 126.4, 125.4 (J = 4.4 Hz), 123.3 (J = 272 Hz), 122.8, 119.9, 39.9, 22.9; ^{11}B{^{1}H} NMR (128 MHz, $CDCl_3$) δ 34.5; ^{19}F NMR (376 MHz, $CDCl_3$) δ –64.3; HRMS (APCI) *m*/*z* calcd for $C_{62}H_{40}B_2F_6S_2$ [M]$^+$ 984.2656, found: 984.2699.

trans-**2b**: Mp. 288.1–288.7 °C; ^{1}H NMR (400 MHz, $CDCl_3$) δ 7.75 (d, J = 8.2 Hz, 4H), 7.59 (d, J = 6.4 Hz, 4H), 7.53–7.45 (m, 12H), 7.30 (d, J = 6.4 Hz, 2H), 6.88 (d, J = 8.7 Hz, 4H), 6.70 (d, J = 8.2 Hz, 4H), 4.55 (s, 4H), 2.20 (s, 6H); ^{13}C{^{1}H} NMR (151 MHz, $CDCl_3$) δ 147.0, 146.6, 142.0, 141.1, 139.4, 137.8, 137.2, 132.8, 130.0, 129.5, 128.7 (J = 32.8 Hz), 127.8, 127.6, 126.5, 125.2 (J = 4.4 Hz), 123.4, 123.3 (J = 272 Hz), 120.1, 39.2, 23.3, two signals were not observed due to the overlap with other signals; ^{11}B{^{1}H} NMR (128 MHz, $CDCl_3$) δ 37.3; ^{19}F NMR (376 MHz, $CDCl_3$) δ –64.4; HRMS (APCI) *m*/*z* calcd for $C_{62}H_{40}B_2F_6S_2$ [M]$^+$ 984.2656, found: 984.2648.

2. X-ray Crystallographic Analysis

Structural Analysis of cis-1a. A single crystal of *cis*-**1a** was obtained by slow diffusion of MeOH into a CH_2Cl_2 solution of *cis*-**1a**. Intensity data were collected at 123 K on a Rigaku Single Crystal X-ray diffractometer equipped with FR-X generator, Varimax optics, and PILATUS 200K photon counting detector with MoKα radiation (λ = 0.71073 Å). A total of 36613 reflections were measured at the maximum 2θ angle of 60.0°, of which 9243 were independent reflections (R_{int} = 0.0226). The structure was solved by direct methods (SHELXT–2018/2)[S4] and reflections by full-matrix least squares procedures of F^2 for all reflections (SHELXL–2018/1).[S5] All non-hydrogen atoms were refined anisotropically and all hydrogen atoms were placed using AFIX instructions. The crystal data are as follows: $C_{81}H_{70}B_4Cl_2S_4$; FW = 1285.75, crystal size 0.20 × 0.20 × 0.20 mm^3, *monoclinic*, $P2_1/c$, a = 16.0140(3) Å, b = 14.2052(2) Å, c = 15.6151(3) Å, β = 106.374(2)°, V = 3408.09(11) Å, Z = 2, D_c = 1.253 g cm^{-3}, μ = 0.263 mm^{-1}, R_1 = 0.0392 ($I > 2\sigma(I)$), wR_2 = 0.1091 (all data), GOF = 1.039. Crystallographic data have been deposited at the Cambridge Crystallographic Data Centre with the deposition number CCDC 2387655. This data can be obtained free of charge from The Cambridge Crystallographic Data Centre at www.ccdc.cam.ac.uk/data_request/cif.

Structural Analysis of *cis*-1b. A single crystal of *cis*-**1b** was obtained by slow diffusion of MeOH into a CH_2Cl_2 solution of *cis*-**1b**. Intensity data were collected at 123 K on a Rigaku Single Crystal X-ray diffractometer equipped with FR-X generator, Varimax optics, and PILATUS 200K photon counting detector with MoKα radiation (λ = 0.71073 Å). A total of 52878 reflections were measured at the maximum 2θ angle of 60.0°, of which 18939 were independent reflections (R_{int} = 0.0650). The structure was solved by direct methods (SHELXT–2018/2)[S4] and reflections by full-matrix least squares procedures of F^2 for all reflections (SHELXL–2018/1).[S5] All non-hydrogen atoms were refined anisotropically and all hydrogen atoms were placed using AFIX instructions. The crystal data are as follows: $C_{42}H_{32}B_2F_6S_2$; FW = 736.41, crystal size 0.10 × 0.05 × 0.01 mm^3, *triclinic*, P–1, a = 13.4613(4) Å, b = 15.9611(4) Å, c = 18.0475(5) Å, α = 88.617(2)°, β = 74.222(2)°, γ = 75.940(2)°, V = 3616.09(18) Å, Z = 4, D_c = 1.353 g cm^{-3}, μ = 0.209 mm^{-1}, R_1 = 0.0641 ($I > 2\sigma(I)$), wR_2 = 0.1615 (all data), GOF = 1.022. Crystallographic

data have been deposited at the Cambridge Crystallographic Data Centre with the deposition number CCDC 2387656. This data can be obtained free of charge from The Cambridge Crystallographic Data Centre at www.ccdc.cam.ac.uk/data_request/cif.

Structural Analysis of *trans*-2a. A single crystal of *trans*-**2a** was obtained by slow diffusion of *n*-hexane into a CH_2Cl_2 solution of *trans*-**2a**. Intensity data were collected at 100 K on a STOE IPDS II two-circle diffractometer equipped with a Xenocs GeniX 3D HS microfocus MoKα X-ray source (λ = 0.71073 Å). A total of 28668 reflections were measured at the maximum 2θ angle of 51.4°, of which 5366 were independent reflections (R_{int} = 0.0977). The structure was solved by direct methods (SHELXT)[S4] and reflections by full-matrix least squares procedures of F^2 for all reflections (SHELXL–2018/3).[S5] All non-hydrogen atoms were refined anisotropically and all hydrogen atoms were placed using AFIX instructions. The crystal data are as follows: $C_{72}H_{70}B_2S_2$; FW = 1021.02, crystal size 0.26 × 0.16 × 0.10 mm^3, *tetragonal*, $I4_1/a$, a = 28.7061(7) Å, b = 28.7061(7) Å, c = 13.7250(12) Å, V = 11310.0(11) Å, Z = 8, D_c = 1.199 g cm^{-3}, μ = 0.138 mm^{-1}, R_1 = 0.0733 ($I > 2\sigma(I)$), wR_2 = 0.2245 (all data), GOF = 1.048. Crystallographic data have been deposited at the Cambridge Crystallographic Data Centre with the deposition number CCDC 2416563. This data can be obtained free of charge from The Cambridge Crystallographic Data Centre at www.ccdc.cam.ac.uk/data_request/cif.

Structural Analysis of *trans*-2b. A single crystal of *trans*-**2b** was obtained by slow diffusion of MeOH into a CH_2Cl_2 solution of *trans*-**2b**. Intensity data were collected at 123 K on a Rigaku Single Crystal X-ray diffractometer equipped with FR-X generator, Varimax optics, and PILATUS 200K photon counting detector with MoKα radiation (λ = 0.71073 Å). A total of 13767 reflections were measured at the maximum 2θ angle of 60.0°, of which 6341 were independent reflections (R_{int} = 0.0241). The structure was solved by direct methods (SHELXT–2018/2)[S4] and reflections by full-matrix least squares procedures of F^2 for all reflections (SHELXL–2018/1).[S5] All non-hydrogen atoms were refined anisotropically and all hydrogen atoms were placed using AFIX instructions. The crystal data are as follows: $C_{63}H_{42}B_2Cl_2F_6S_2$; FW = 1069.60, crystal size 0.10 × 0.05 × 0.05 mm^3, *triclinic*, P–1, a = 8.0487(3) Å, b = 11.4610(4) Å, c = 13.8943(4) Å, α =

83.718(2)°, β = 78.136(3)°, γ = 84.362(3)°, V = 1243.00(7) Å, Z = 1, D_c = 1.429 g cm^{-3}, μ = 0.281 mm^{-1}, R_1 = 0.0457 ($I > 2\sigma(I)$), wR_2 = 0.1196 (all data), GOF = 1.068. Crystallographic data have been deposited at the Cambridge Crystallographic Data Centre with the deposition number CCDC 2387657. This data can be obtained free of charge from The Cambridge Crystallographic Data Centre at www.ccdc.cam.ac.uk/data_request/cif.

3. Analysis of B–S Interaction

NBO Analysis. Geometry optimizations were performed using the Gaussian 16 Revision C.01 suite of programs[S6] at the PBE0/6-31G(d) level of theory. All the optimized structures have been confirmed as the energy minima by frequency analysis at the same level of theory, which showed only positive values. Natural bond orbital (NBO) analysis (NBO 7.0)[S7] implemented in the Gaussian 16 Revision C.01 suite of programs were conducted for *cis*-**1a**, *cis*-**1b**, *trans*-**2a**, *trans*-**2b**, *cis*-**5**, and **A** at the B3LYP-D3/6-311+G(d,p) level of theory. The results of the NBO analysis was summarized in Table 2.

Comparison of Chemical Shifts in ^{11}B NMR Spectra.

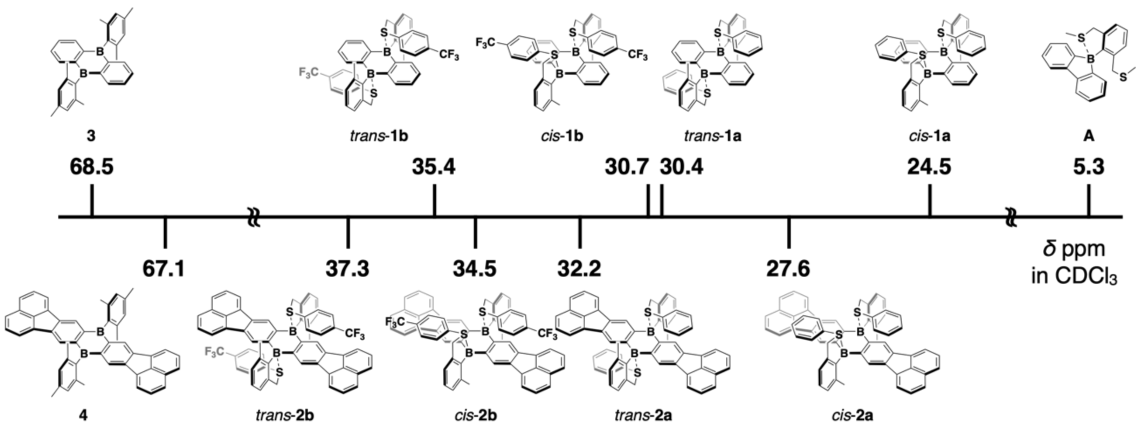

Figure S1. Chemical shifts of **1**–**4** and **A**[S8] in ^{11}B NMR spectra in $CDCl_3$.

4. Thermal *cis–trans* Isomerization

Methods. ^{1}H NMR spectra were recorded with a JEOL JNM-ECS-400 spectrometer (400 MHz) for *cis*-**1a** in toluene-d_6 after heating at 363, 353, 343, and 333 K, respectively. The ratio of *cis*- and *trans*-isomers was determined from the integral values of the benzylic protons (Figure S2a), following that equilibrium had been reached by monitoring the *cis/trans* ratios over time (Figure S2b). As shown in Figure S2b, equilibrium is reached after 4 h at 363 K, whereas it requires 36 h at 333 K.

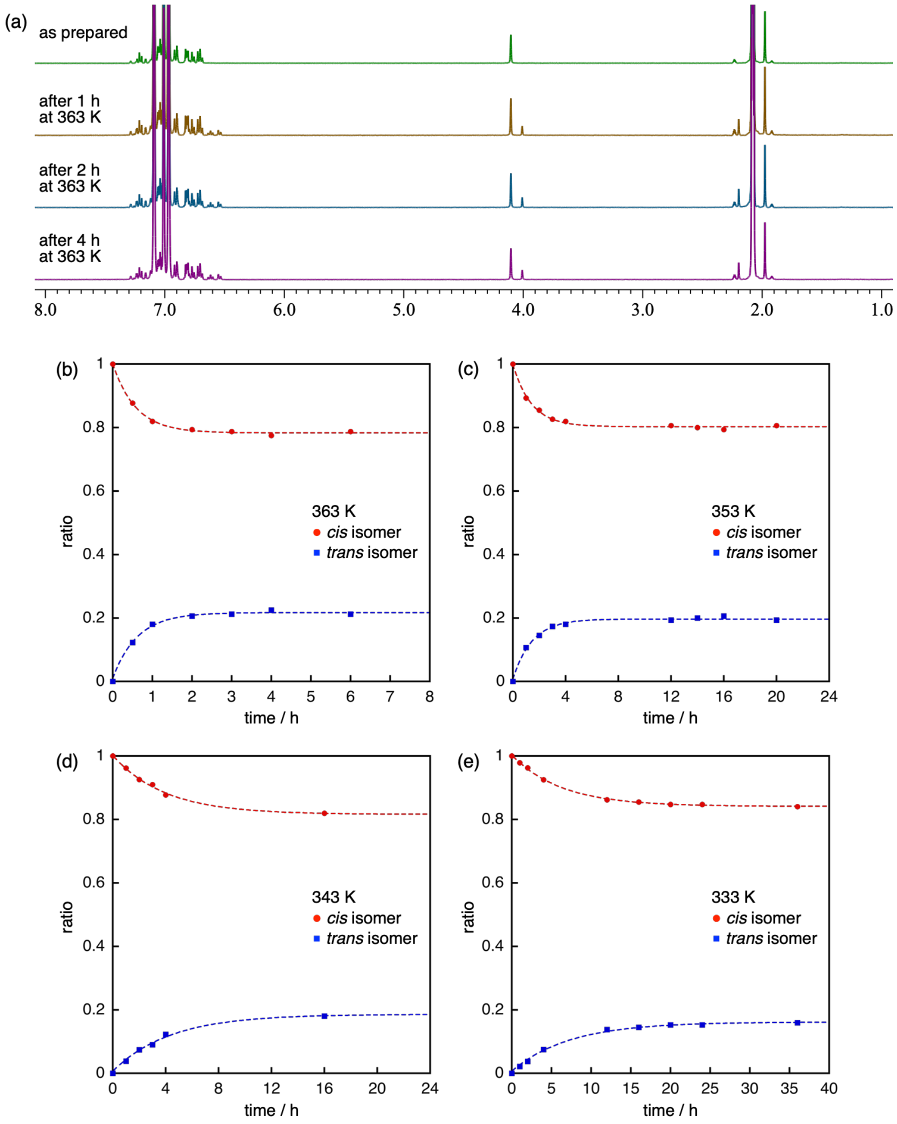

Figure S2. (a) ^{1}H NMR spectra of *cis*-**1a** in toluene-d_8 upon heating at 363 K (400 MHz). Time-dependent changes in the *cis*/*trans* isomer ratio of *cis*-**1a** at (b) 363 K, (c) 353 K, (d) 343 K, and (e) 333 K.

Table S1. Equilibrium Constants (K_{eq}) for *cis–trans* Isomerization of *cis*-**1a** and *cis*-**1b**

Temperature/K	K_{eq} (*cis*-**1a**)	K_{eq} (*cis*-**1b**)
363	0.28	0.48
353	0.24	0.44
343	0.23	0.41
333	0.19	0.37

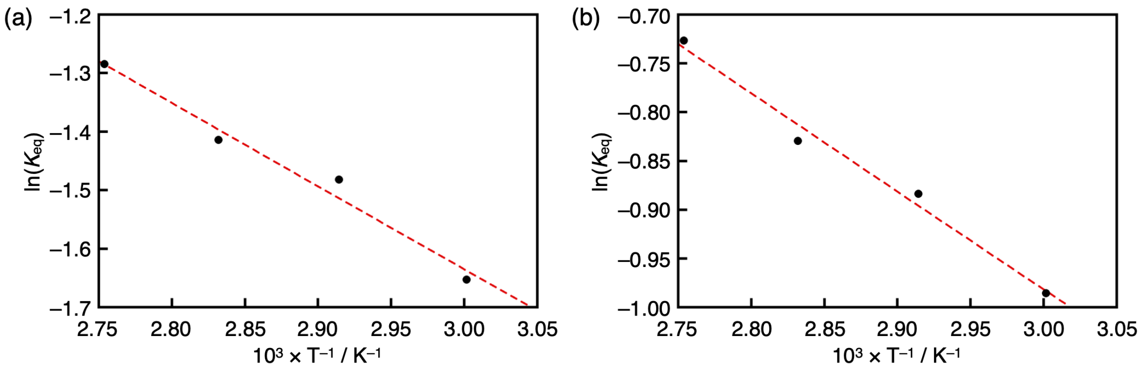

Figure S3. Van't Hoff plots for *cis–trans* isomerization of (a) *cis*-**1a** (R^2 = 0.96744) and (b) *cis*-**1b** (R^2 = 0.97900).

Table S2. Thermodynamic Parameters for *cis-trans* Isomerization of *cis*-**1a** and *cis*-**1b** in Toluene-d_8

Compound	ΔH /kJ mol^{-1}	ΔS /J mol^{-1} K^{-1}	ΔG /kJ mol^{-1} [a]	$K_{eq,298}$ [b]	*cis*/*trans* ratio[c]
cis-**1a**	11.8	21.9	5.30	0.12	89.5/10.5
cis-**1b**	8.4	16.9	3.32	0.26	79.2/20.8

[a] Gibbs free energy changes at 298 K. [b] Calculated equilibrium constants (K_{eq}) for *cis–trans* isomerization at 298 K. [c] Estimated ratios of *cis* and *trans* isomers at 298 K.

5. Photophysical Properties

Methods. UV–vis absorption and fluorescence spectra were measured with a Shimadzu UV-3600 Plus spectrometer and a JASCO FP-8500 spectrofluorometer, respectively, using dilute sample solutions in a 1 cm square quartz cuvette. Absolute fluorescence quantum yields were determined with a Hamamatsu Absolute PL Quantum Yield spectrometer C11347-01 calibrated integrating sphere system.

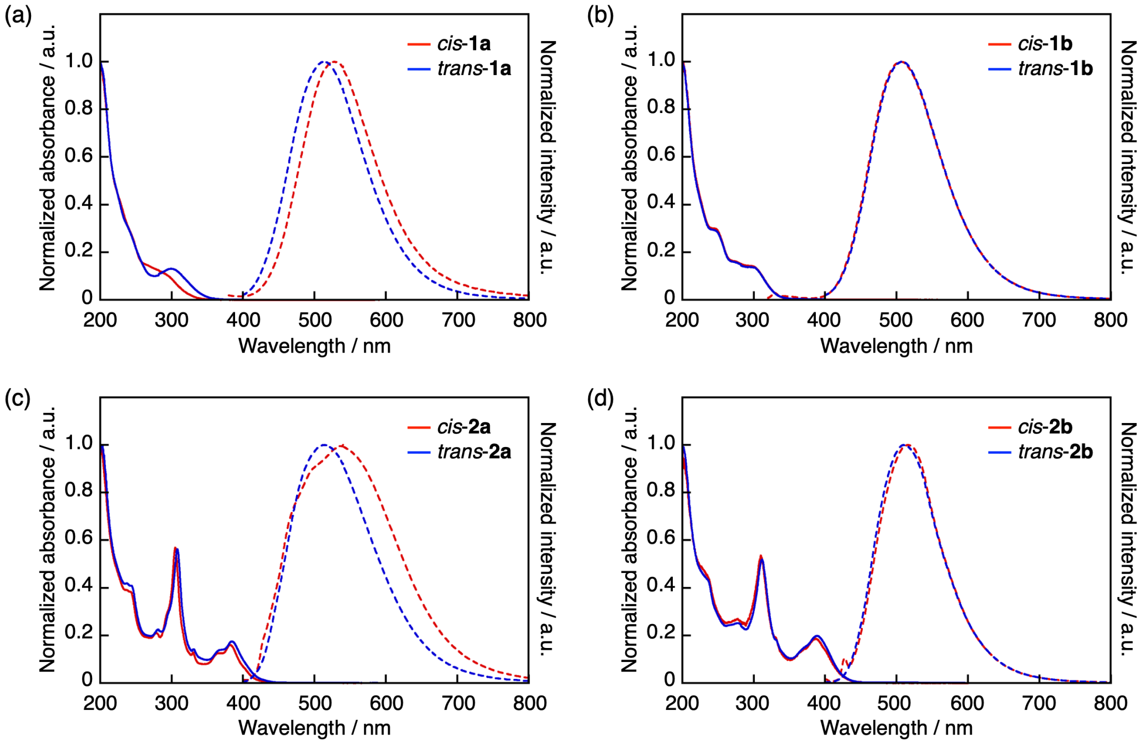

Figure S4. UV–vis absorption (solid lines) and fluorescence (dashed lines) spectra of (a) *cis*/*trans*-**1a**, (b) *cis*/*trans*-**1b**, (c) *cis*/*trans*-**2a**, and (d) *cis*/*trans*-**2b** in cyclohexane.

Table S3. Photophysical Data for Arylsulfide-Coordinate Diboraanthracenes and Reference Compounds

Compound	Solvent	λ_{abs} /nm[a]	ε /10^4 $M^{-1}cm^{-1}$	λ_{em} /nm	Stokes shift /cm^{-1}	Φ_F[b]
cis-**1a**	cyclohexane	282	–[c]	526	16500	0.015
	CH_2Cl_2	277	1.50	572	18600	0.011
cis-**1b**	cyclohexane	298	1.61	506	13800	0.031
	CH_2Cl_2	293	1.62	533	15400	0.018
cis-**2a**	cyclohexane	382	–[c]	540	7660	0.024
	toluene	383	2.90	568	8500	0.013
	CH_2Cl_2	382	3.06	595	9370	0.030
cis-**2b**	cyclohexane	386	–[c]	516	6530	0.037
	toluene	386	3.33	532	7110	0.010
	CH_2Cl_2	384	3.15	532	7240	0.040
trans-**1a**	cyclohexane	300	1.48	512	13800	0.028
trans-**1b**	cyclohexane	297	1.74	507	13900	0.034
trans-**2a**	cyclohexane	385	2.86	515	6560	0.033
trans-**2b**	cyclohexane	389	–[c]	509	6060	0.036
**3**[S9]	cyclohexane	406	0.0944	413	476	0.02
**4**[S10]	cyclohexane	436	4.34	522	3780	0.26

[a] Only the longest absorption maximum wavelengths are shown. [b] Absolute fluorescence quantum yields determined by a calibrated integrating sphere system within ±0.1% error. [c] Not determined due to low solubility.

Temperature-Dependent Photoluminescence Spectroscopy. Photoluminescence spectra were measured with a JASCO FP-8500 spectrofluorometer. 2-Methyltetrahydrofuran was dried over CaH_2 and distilled prior to use. The sample solution (1.35×10^{-5} M for *cis*-**2a**, 1.18×10^{-5} M for *cis*-**2b**) was degassed by two freeze-pump-thaw cycles before measurements. The solution in a 1 cm square quartz cuvette equipped with a J. Young stopcock was cooled in an Oxford Optistat DN cryostat.

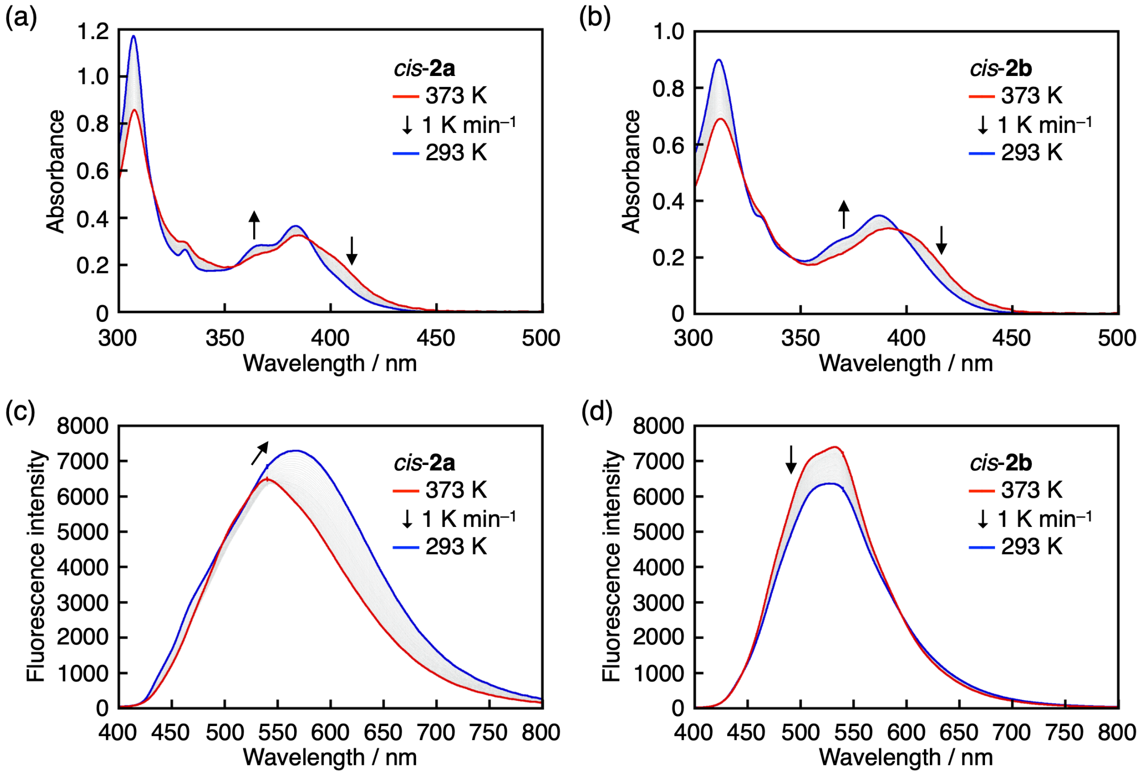

Figure S5. (a,b) UV–vis absorption and (c,d) fluorescence spectra of (a,c) *cis*-**2a** and (b,d) *cis*-**2b** upon cooling from 373 to 293 K at 1 K min^{-1} in toluene.

6. Theoretical Study

Methods. Geometry optimizations were performed using the Gaussian 16 Revision C.01 suite of programs[S6] at the PBE0/6-31G(d) level of theory. All the optimized structures have been confirmed as the energy minima by frequency analysis at the same level of theory, which showed only positive values. The optimized geometries were used for the TD-DFT calculations at the PBE0/6-31G(d) level of theory.

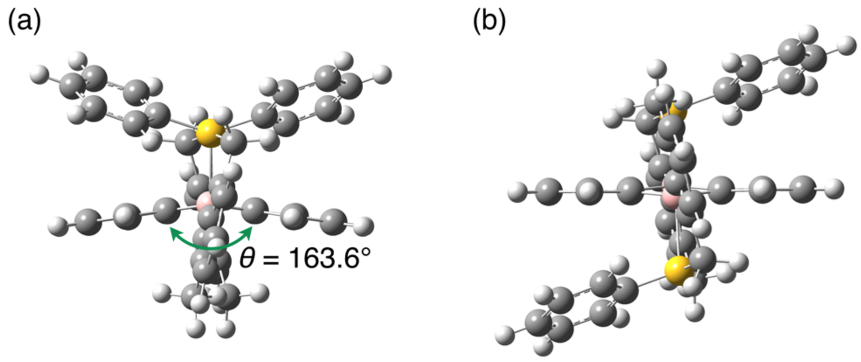

Figure S6. Side views of the optimized structures of (a) *cis*-**1a** and (b) *trans*-**1a** calculated at the PBE0/6-31G(d) level of theory. The bent angle θ between the two benzene rings in the DBA moiety for *cis*-**1a** is 163.6°.

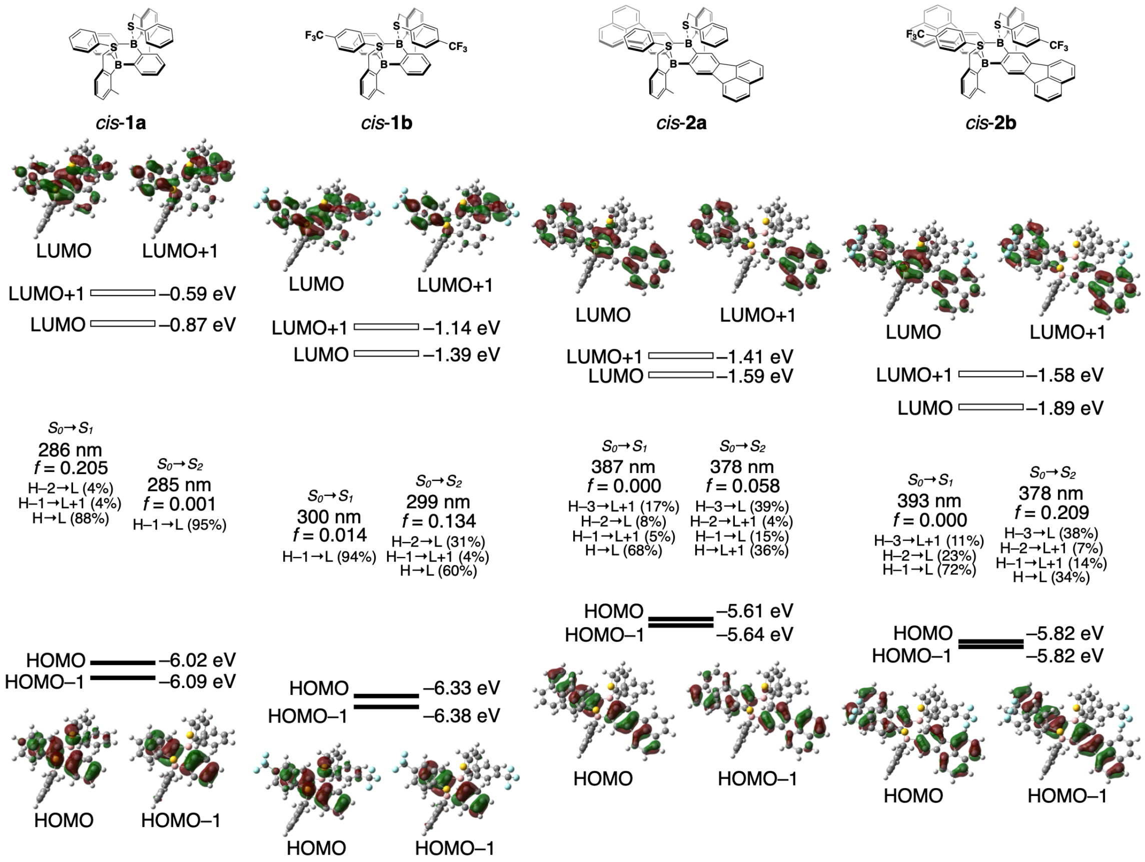

Figure S7. Kohn-Sham molecular orbitals for *cis*-**1a**, *cis*-**1b**, *cis*-**2a**, and *cis*-**2b** in S_0 optimized structures. TD-DFT calculations were carried out at the PBE0/6-31G(d) level of theory.

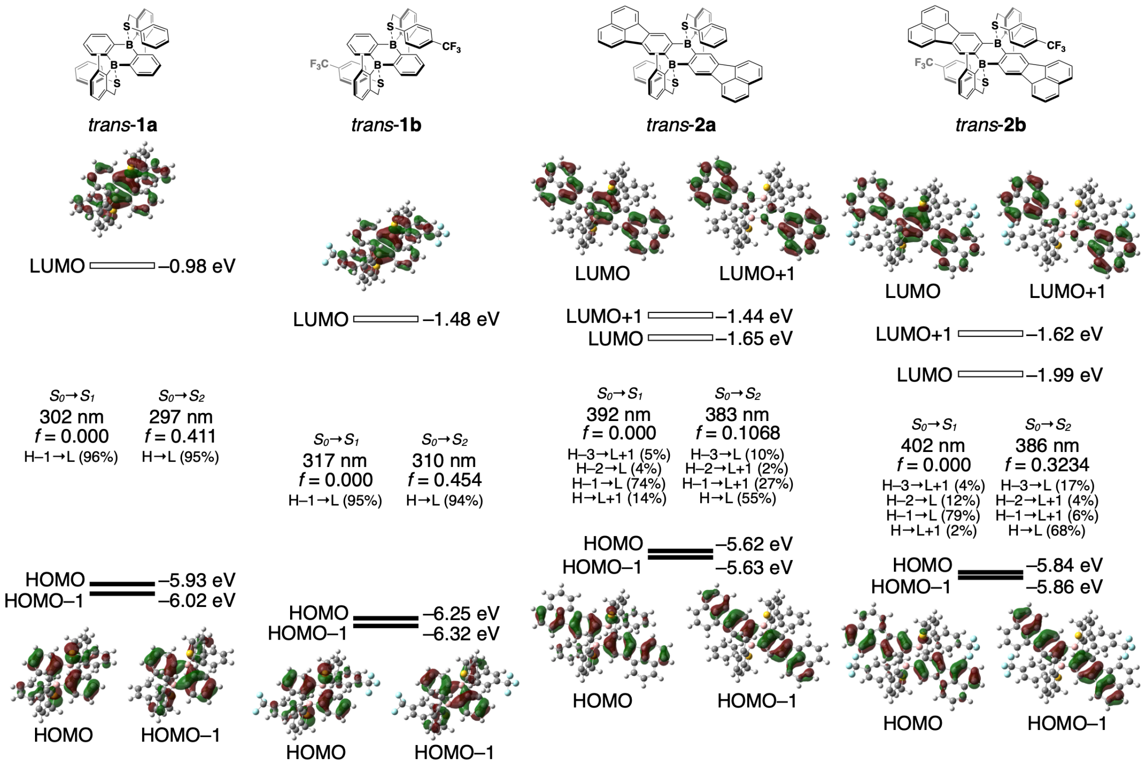

Figure S8. Kohn-Sham molecular orbitals for *trans*-**1a**, *trans*-**1b**, *trans*-**2a**, and *trans*-**2b** in S_0 optimized structures. TD-DFT calculations were carried out at the PBE0/6-31G(d) level of theory.

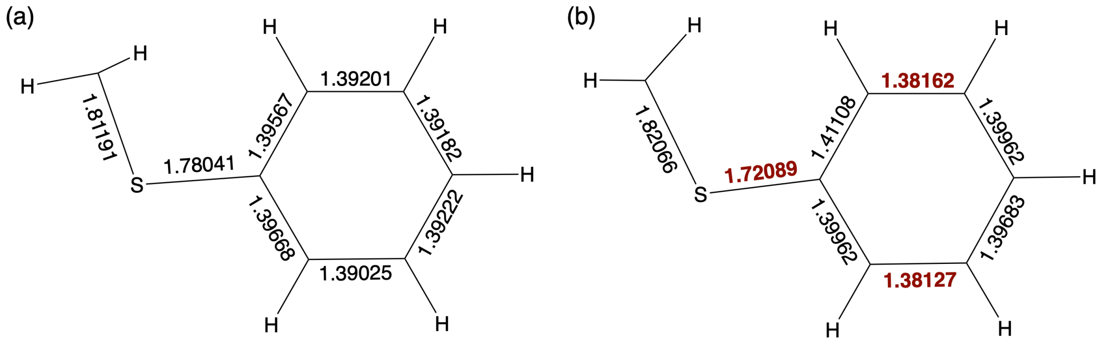

Figure S9. Bond lengths (Å) of the optimized structures in (a) S_0 and (b) S_1 of phenylthio moiety of *cis*-**1a** calculated at the PBE0/6-31G(d) level of theory.

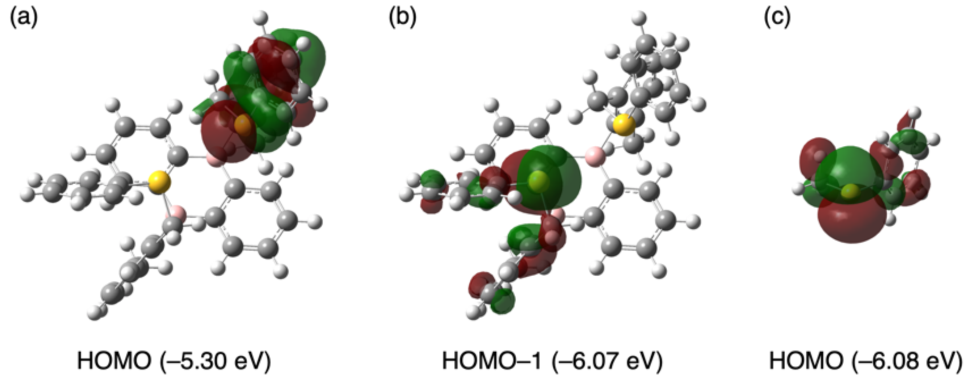

Figure S10. Kohn-Sham molecular orbitals for (a) HOMO and (b) HOMO–1 of *cis*-**1a**, and (c) HOMO of phenylmethylsulfide in the S_1 optimized structures.

Table S4. Cartesian Coordinates (Å) for *cis*-**1a** in the Gas Phase in S_0 Calculated at PBE0/6-31G(d) Level of Theory

atom	*x*	*y*	*z*	*atom*	*x*	*y*	*z*
B	1.44757009	0.47109083	-0.70104647	H	-6.42335464	-2.45386523	-2.03337475
B	-1.44757249	-0.47011760	-0.70130655	C	3.13018223	1.84009212	1.21781662
C	-1.09811029	1.06889798	-0.89164908	H	2.64357399	2.81848710	1.31646789
C	-0.51680387	3.80420003	-1.24443644	H	3.86611103	1.75577989	2.02417683
C	-2.11622795	2.01874389	-1.06251687	C	2.55006141	0.35389424	-3.54559305
C	0.24855546	1.50945797	-0.89168647	H	1.53231547	0.76019043	-3.53061299
C	0.50756870	2.87720147	-1.07686855	H	2.45589426	-0.73218315	-3.43513572
C	-1.83968526	3.37185015	-1.23377542	H	2.98644372	0.56082382	-4.52808707
H	-3.15275301	1.68589824	-1.06821233	C	-2.55023133	-0.34850937	-3.54605712
H	1.54018201	3.22159879	-1.11092010	H	-2.98589451	-0.55522876	-4.52891493
H	-2.65169603	4.08365903	-1.36488046	H	-1.53196656	-0.75346458	-3.53088838
H	-0.28392201	4.85682204	-1.39026799	H	-2.45753686	0.73761772	-3.43473600
C	-0.24849728	-1.50824337	-0.89341268	C	-3.12971034	-1.84188132	1.21501839
C	1.83978104	-3.37018649	-1.23789664	H	-3.86538646	-1.75913528	2.02176952
C	1.09815886	-1.06766058	-0.89297689	H	-2.64260562	-2.82020781	1.31199499
C	-0.50748170	-2.87576460	-1.08028468	S	1.80130739	0.64059524	1.49749495
C	0.51690047	-3.80253930	-1.24901491	S	-1.80113429	-0.64232328	1.49591721
C	2.11629301	-2.01728451	-1.06505689	C	2.67497767	-0.81118746	2.04412663
H	-1.54009272	-3.22012201	-1.11480988	C	3.87480636	-3.15849900	2.94379956
H	0.28403229	-4.85499053	-1.39609666	C	4.02880797	-1.03828850	1.79197315
H	3.15280293	-1.68439386	-1.07045397	C	1.91930956	-1.75995851	2.73650269
H	2.65179344	-4.08182553	-1.36989719	C	2.52130259	-2.93245905	3.17876584
C	2.90559184	1.01332065	-1.11947524	C	4.62286837	-2.20981719	2.25282369
C	5.44200041	2.06690024	-1.77250017	H	4.61486432	-0.32242509	1.22464278
C	3.70671492	1.65557268	-0.16404545	H	0.86497640	-1.57865281	2.92651906
C	3.38572154	0.95173155	-2.44587441	H	1.92898997	-3.66834197	3.71534631
C	4.64812785	1.46985327	-2.74715741	H	5.67781629	-2.38274472	2.05807037

C	4.96526098	2.17350407	-0.47212918	H	4.34466534	-4.07245940	3.29604890
H	5.01304676	1.40687824	-3.77044797	C	-2.67504453	0.80842274	2.04495598
H	5.56355401	2.65976695	0.29624560	C	-3.87527727	3.15395647	2.94864504
H	6.42340867	2.45673719	-2.02999656	C	-4.02891288	1.03569217	1.79320865
C	-2.90552012	-1.01177396	-1.12095370	C	-1.91951600	1.75614653	2.73891909
C	-5.44193008	-2.06441620	-1.77535813	C	-2.52172366	2.92777273	3.18320107
C	-3.70659677	-1.65543610	-0.16641059	C	-4.62318553	2.20632415	2.25606415
C	-3.38569561	-0.94820013	-2.44720705	H	-4.61482695	0.32066797	1.22467311
C	-4.64811142	-1.46593996	-2.74917200	H	-0.86514643	1.57470991	2.92860473
C	-4.96515160	-2.17290848	-0.47513726	H	-1.92953193	3.66285329	3.72101211
H	-5.01308066	-1.40150288	-3.77235596	H	-5.67816745	2.37939124	2.06162438
H	-5.56343103	-2.66026657	0.29255336	H	-4.34530223	4.06722484	3.30246390

Table S5. Cartesian Coordinates (Å) for *cis*-**1b** in the Gas Phase in S_0 Calculated at PBE0/6-31G(d) Level of Theory

atom	*x*	*y*	*z*	*atom*	*x*	*y*	*z*
B	-1.01902876	-1.12664429	-1.32654371	H	-2.48629684	-3.45159638	1.43680167
B	1.01907849	1.12581234	-1.32723757	C	-2.04718569	-1.58882972	-4.14498348
C	1.48400201	-0.38096384	-1.49786994	H	-2.33482528	-1.98749335	-5.12299098
C	2.34283013	-3.04687959	-1.81212027	H	-0.96007455	-1.44937095	-4.13978284
C	2.84101283	-0.69964298	-1.65567443	H	-2.49263140	-0.59341686	-4.03804766
C	0.53481847	-1.43362580	-1.49370338	C	2.04752332	1.58603190	-4.14587279
C	0.99145516	-2.75112371	-1.65965715	H	2.33471268	1.98432203	-5.12416545
C	3.27514454	-2.01332429	-1.80736291	H	0.96048808	1.44603051	-4.14031082
H	3.57441265	0.10482402	-1.66743749	H	2.49349797	0.59089538	-4.03853548
H	0.26707386	-3.56365930	-1.69086370	C	1.81059820	3.14417164	0.62989358
H	4.33428220	-2.22880708	-1.92851102	H	2.48610981	3.45263378	1.43467633
H	2.66636712	-4.07710813	-1.94257136	H	0.89877263	3.74782586	0.72041451
C	-0.53475088	1.43268275	-1.49470082	S	-1.25124978	-1.44828938	0.93485659
C	-3.27505356	2.01218367	-1.80891050	S	1.25114994	1.44895370	0.93397811
C	-1.48393582	0.38002068	-1.49822407	C	-2.73190688	-0.61254640	1.44856999
C	-0.99137034	2.75007043	-1.66156574	C	-4.94331394	0.85455624	2.28469617
C	-2.34273372	3.04573169	-1.81430103	C	-4.01856168	-1.10283715	1.21824768
C	-2.84093716	0.69860111	-1.65632552	C	-2.55484029	0.61821019	2.08625433
H	-0.26697808	3.56257553	-1.69328435	C	-3.65909208	1.35011152	2.49869440
H	-2.66625829	4.07587449	-1.94545756	C	-5.11923193	-0.36969495	1.64572828
H	-3.57433734	-0.10587197	-1.66761214	H	-4.17129169	-2.03602376	0.68662626
H	-4.33418262	2.22759284	-1.93026418	H	-1.55308807	1.00227149	2.25637973
C	-2.02320099	-2.32120175	-1.71364178	H	-3.52242804	2.30961730	2.98692996
C	-3.73010761	-4.48010333	-2.34321767	H	-6.12091450	-0.74606311	1.46359849
C	-2.40658066	-3.26443239	-0.74876596	C	2.73178325	0.61354252	1.44828372
C	-2.47950043	-2.51092504	-3.03662064	C	4.94315021	-0.85310264	2.28533568
C	-3.33112795	-3.58019032	-3.32649602	C	4.01845078	1.10374527	1.21783441
C	-3.25646488	-4.33055510	-1.04603147	C	2.55469099	-0.61689474	2.08657496

H	-3.68503511	-3.71012181	-4.34718984	C	3.65892172	-1.34856840	2.49948091
H	-3.53956950	-5.04043347	-0.27101961	C	5.11909554	0.37083498	1.64576857
H	-4.39902465	-5.30002718	-2.59105857	H	4.17121786	2.03666362	0.68575491
C	2.02328933	2.32010356	-1.71504721	H	1.55293382	-1.00090149	2.25679569
C	3.73031443	4.47851778	-2.34593004	H	3.52223220	-2.30783034	2.98818531
C	2.40660709	3.26397142	-0.75077302	H	6.12078638	0.74712836	1.46352151
C	2.47971111	2.50890924	-3.03811125	C	-6.13416176	1.61431337	2.79069598
C	3.33139824	3.57795120	-3.32863577	C	6.13401121	-1.61254541	2.79177307
C	3.25654958	4.32986622	-1.04868311	F	-6.44868519	1.25697293	4.04869268
H	3.68539966	3.70718741	-4.34938564	F	-5.91074678	2.93776345	2.80445487
H	3.53960215	5.04026410	-0.27412860	F	-7.22284637	1.39238616	2.03742287
H	4.39927310	5.29825713	-2.59426895	F	5.91040647	-2.93594554	2.80692493
C	-1.81069921	-3.14370550	0.63187262	F	6.44895571	-1.25397401	4.04931467
H	-0.89889785	-3.74732218	0.72288678	F	7.22251807	-1.39153540	2.03796734

Table S6. Cartesian Coordinates (Å) for *cis*-**2a** in the Gas Phase in S_0 Calculated at PBE0/6-31G(d) Level of Theory

atom	*x*	*y*	*z*	*atom*	*x*	*y*	*z*
B	-0.19486650	-1.50589813	-0.23769062	C	-4.57926635	-2.08915637	3.28833114
B	0.19480552	1.50594790	-0.23783626	C	-2.69792295	-3.17842016	2.22958958
C	1.43679001	0.53439001	-0.42975610	C	-2.46063525	-0.94451579	3.13336016
C	3.64780285	-1.17613594	-0.74599640	C	-3.79332085	-0.96546260	3.52840275
C	2.72928714	1.06579912	-0.59237544	C	-4.02704836	-3.19198816	2.64365132
C	1.25858228	-0.87300475	-0.42276583	H	-2.29048434	-4.03053479	1.69518198
C	2.37831094	-1.71044508	-0.59268133	H	-1.84472239	-0.07030501	3.32577102
C	3.82644662	0.23229712	-0.73955106	H	-4.21673205	-0.09922348	4.02876174
H	2.86205258	2.14630109	-0.60302715	H	-4.63574142	-4.07060325	2.44777081
H	2.23868885	-2.79034077	-0.62129969	H	-5.62005008	-2.10335133	3.59860196
C	-1.25862838	0.87309190	-0.42275922	C	1.91322532	2.05186212	2.48256417
C	-3.82651187	-0.23216135	-0.73943190	C	4.57925275	2.08833006	3.28891679
C	-1.43685226	-0.53430029	-0.42970406	C	2.69832205	3.17775337	2.22961137
C	-2.37834624	1.71055814	-0.59264415	C	2.46025034	0.94432271	3.13433646
C	-3.64784996	1.17627058	-0.74590974	C	3.79292754	0.96500213	3.52943661
C	-2.72936450	-1.06568487	-0.59226552	C	4.02742503	3.19106856	2.64373505
H	-2.23869697	2.79045039	-0.62124545	H	2.29116515	4.02977740	1.69484178
H	-2.86216080	-2.14618395	-0.60285811	H	1.84403395	0.07040810	3.32711926
C	-0.33175514	-3.05648282	-0.65132068	H	4.21602521	0.09884856	4.03021046
C	-0.47237329	-5.79937132	-1.30309057	H	4.63642321	4.06939522	2.44750956
C	-0.10781202	-4.05526489	0.30775503	H	5.62002870	2.10231884	3.59922392
C	-0.58527781	-3.46399086	-1.97939998	H	-5.60557513	-2.64185248	-0.89957493
C	-0.66067713	-4.82658659	-2.28029175	C	-6.03724882	-1.64734484	-0.98078531
C	-0.18176468	-5.41427443	-0.00041586	C	-5.25917519	-0.51329941	-0.91312041
H	-0.86707777	-5.12939020	-3.30491245	C	-8.05050679	-0.27045000	-1.26978137
H	-0.00909715	-6.16335324	0.77021120	C	-5.89769102	0.74425944	-1.02398049

H	-0.54216903	-6.85315140	-1.56018471	C	-7.43771303	-1.50338199	-1.16066677
C	0.33175894	3.05657139	-0.65118775	C	-7.27466203	0.91753970	-1.20209631
C	0.47258818	5.79947970	-1.30285343	C	-4.96472477	1.80411909	-0.93213898
C	0.10804084	4.05532297	0.30796846	H	-8.04784820	-2.40150000	-1.21587289
C	0.58520114	3.46412722	-1.97927311	H	-8.78749940	2.46628825	-1.43552792
C	0.66069973	4.82672552	-2.28012153	H	-9.12731306	-0.20780047	-1.40869742
C	0.18209639	5.41433920	-0.00016836	C	-5.43474602	3.09502969	-1.02664545
H	0.86702717	5.12955124	-3.30474962	H	-4.76854644	3.95237543	-0.96842847
H	0.00959950	6.16339875	0.77051602	C	-6.82697559	3.30247273	-1.20902805
H	0.54244941	6.85326766	-1.55989787	H	-7.19265808	4.32343292	-1.28460208
C	0.29238110	-3.61576223	1.69457767	C	-7.72897398	2.26023766	-1.29491018
H	1.38374053	-3.60196501	1.80709628	H	5.60547294	2.64201285	-0.89981903
H	-0.11136772	-4.24413736	2.49526311	C	6.03715706	1.64750733	-0.98100464
C	-0.75777739	-2.45262842	-3.08057974	C	5.25910034	0.51345391	-0.91328584
H	0.04455855	-1.70644605	-3.05863869	C	8.05042704	0.27063419	-1.27002088
H	-1.70056295	-1.90321001	-2.97944408	C	5.89763117	-0.74409861	-1.02413621
H	-0.74882699	-2.93515524	-4.06312397	C	7.43761894	1.50355974	-1.16092083
C	0.75755402	2.45279516	-3.08050881	C	7.27459922	-0.91736423	-1.20228654
H	1.70028802	1.90327196	-2.97943095	C	4.96468114	-1.80396933	-0.93225013
H	0.74863023	2.93537336	-4.06302795	H	8.04773946	2.40168526	-1.21616685
H	-0.04485910	1.70669578	-3.05858893	H	8.78745083	-2.46609705	-1.43572629
C	-0.29199090	3.61592386	1.69487581	H	9.12723051	0.20799546	-1.40896389
H	0.11211417	4.24423991	2.49542872	C	5.43471729	-3.09487572	-1.02674397
H	-1.38332814	3.60247207	1.80762248	H	4.76853076	-3.95222963	-0.96849560
S	-0.21484909	-1.89926240	1.97189195	C	6.82694437	-3.30230396	-1.20915861
S	0.21485873	1.89931765	1.97228904	H	7.19263861	-4.32326071	-1.28472196
C	-1.91322346	-2.05216169	2.48209198	C	7.72892625	-2.26005797	-1.29508518

Table S7. Cartesian Coordinates (Å) for *cis*-**2b** in the Gas Phase in S_0 Calculated at PBE0/6-31G(d) Level of Theory

atom	*x*	*y*	*z*	*atom*	*x*	*y*	*z*
B	-0.41626378	-1.45460376	-0.81369411	C	-4.02929611	-0.47501680	2.79109307
B	0.41615328	1.45461767	-0.81373212	C	-4.45093192	-2.73080561	2.05253119
C	1.50257589	0.31300550	-0.94314808	H	-2.77235422	-3.81930973	1.29220532
C	3.43938585	-1.71506855	-1.17020584	H	-1.99254332	0.19373444	2.65241490
C	2.86760718	0.64140302	-1.03350604	H	-4.39211588	0.46674613	3.18861033
C	1.11211358	-1.05218187	-0.95432448	H	-5.14494985	-3.54696702	1.87396333
C	2.09667683	-2.04966446	-1.09110270	C	2.21851343	1.83380729	1.95499578
C	3.83187866	-0.35056404	-1.12316963	C	4.91644264	1.52449158	2.57104013
H	3.16377578	1.68893893	-1.02581220	C	3.10791880	2.88921493	1.73734732
H	1.79457248	-3.09465165	-1.14473713	C	2.68310224	0.62872688	2.48791767
C	-1.11220780	1.05222128	-0.95428737	C	4.02944392	0.47490481	2.79090638
C	-3.83197213	0.35063548	-1.12319907	C	4.45105322	2.73071913	2.05241988
C	-1.50268229	-0.31296170	-0.94315549	H	2.77240243	3.81931501	1.29238860

C	-2.09676301	2.04972240	-1.09102059	H	1.99267362	-0.19380744	2.65240460
C	-3.43947342	1.71514140	-1.17015600	H	4.39229436	-0.46690858	3.18828018
C	-2.86770883	-0.64134209	-1.03355097	H	5.14507541	3.54685529	1.87376542
H	-1.79464369	3.09470781	-1.14461656	H	-5.97238275	-1.75145733	-1.05057533
H	-3.16388864	-1.68887609	-1.02593320	C	-6.24752656	-0.70397750	-1.14211236
C	-0.79322973	-2.97001818	-1.18512537	C	-5.29972047	0.29418254	-1.18338050
C	-1.36431968	-5.66083589	-1.82196815	C	-8.03865063	0.96636894	-1.31840580
C	-0.69788081	-3.99031439	-0.22623029	C	-5.74152182	1.63414996	-1.28361699
C	-1.14394047	-3.33599750	-2.50343688	C	-7.61861751	-0.34471369	-1.20986120
C	-1.43112275	-4.67157392	-2.79760121	C	-7.08504856	2.01832862	-1.35710457
C	-0.98567039	-5.32182383	-0.52914781	C	-4.65277919	2.53705159	-1.28619787
H	-1.70935967	-4.93919085	-3.81485467	H	-8.36293753	-1.13560349	-1.17114868
H	-0.90885639	-6.08794686	0.24032145	H	-8.35321459	3.78045949	-1.51759569
H	-1.59844012	-6.69235459	-2.07163298	H	-9.10057718	1.19516916	-1.36756394
C	0.79315097	2.97005205	-1.18500738	C	-4.92268885	3.88443095	-1.37918121
C	1.36440782	5.66086431	-1.82174027	H	-4.12951461	4.62809883	-1.38971488
C	0.69791061	3.99030594	-0.22604902	C	-6.27545595	4.30390163	-1.46527755
C	1.14383413	3.33608075	-2.50331177	H	-6.48395570	5.36802529	-1.54082911
C	1.43109363	4.67165238	-2.79742731	C	-7.33152345	3.41357315	-1.45374183
C	0.98579416	5.32180520	-0.52892074	H	5.97229142	1.75151725	-1.05025015
H	1.70929514	4.93929746	-3.81468306	C	6.24743443	0.70404852	-1.14190802
H	0.90906951	6.08789437	0.24059167	C	5.29962850	-0.29410290	-1.18333695
H	1.59858324	6.69238190	-2.07135736	C	8.03856707	-0.96628493	-1.31824458
C	-0.20469879	-3.63224643	1.15447190	C	5.74143334	-1.63406222	-1.28367158
H	0.88598346	-3.73299123	1.22237344	C	7.61853030	0.34478685	-1.20960102
H	-0.63497894	-4.25146701	1.94869654	C	7.08496395	-2.01823878	-1.35709428
C	-1.19688689	-2.30983335	-3.60374040	C	4.65269347	-2.53696603	-1.28630864
H	-1.29340186	-2.78811627	-4.58350359	H	8.36284768	1.13567186	-1.17074744
H	-0.29039660	-1.69374415	-3.61382566	H	8.35313839	-3.78036310	-1.51758554
H	-2.04229901	-1.62445334	-3.47537190	H	9.10049584	-1.19508540	-1.36735193
C	1.19671701	2.30996267	-3.60366526	C	4.92260934	-3.88434492	-1.37929218
H	2.04234425	1.62479685	-3.47555138	H	4.12943700	-4.62801463	-1.38984588
H	1.29285060	2.78830235	-4.58343876	C	6.27537880	-4.30381056	-1.46536114
H	0.29038452	1.69364161	-3.61353033	H	6.48388437	-5.36793237	-1.54092317
C	0.20474967	3.63225799	1.15466481	C	7.33144418	-3.41347963	-1.45376394
H	0.63498649	4.25155511	1.94885415	C	-6.37228046	-1.39060657	2.90685230
H	-0.88593922	3.73297853	1.22252764	C	6.37244059	1.39038458	2.90655278
S	-0.49942935	-1.88519624	1.52063721	F	-6.68624667	-2.08322980	4.01748898
S	0.49945239	1.88524930	1.52101647	F	-6.72847115	-0.11717007	3.11887257
C	-2.21844607	-1.83381570	1.95485211	F	-7.14802325	-1.87478772	1.92096566
C	-4.91628316	-1.52461601	2.57128786	F	6.68623402	2.08188172	4.01793177
C	-3.10784491	-2.88924111	1.73725061	F	7.14818031	1.87572818	1.92121178
C	-2.68298302	-0.62878808	2.48791932	F	6.72882856	0.11679155	3.11726244

Table S8. Cartesian Coordinates (Å) for *trans*-**1a** in the Gas Phase in S_0 Calculated at PBE0/6-31G(d) Level of Theory

atom	*x*	*y*	*z*	*atom*	*x*	*y*	*z*
B	-1.31434059	-0.77647758	-0.05904491	H	-5.06290363	-4.71699948	0.86939521
B	1.31399206	0.77685527	0.05866490	C	-1.15681447	-2.63380286	2.33035688
C	-1.11539545	0.41581791	0.97018928	H	-0.19607733	-2.63780130	1.80109254
C	-0.90028132	2.66264711	2.65936865	H	-1.15016536	-3.45316227	3.05643336
C	-2.13905077	0.76174252	1.86580648	H	-1.20592957	-1.68737167	2.88023597
C	0.06393664	1.20153209	0.95213479	C	-3.50852686	-1.30886008	-1.91115695
C	0.13252427	2.32745650	1.78915087	H	-4.52289973	-1.06089205	-2.24040387
C	-2.03906406	1.86367767	2.70952737	H	-3.01300504	-1.82239406	-2.74414517
H	-3.04079655	0.15295720	1.89615322	C	3.50840999	1.30881284	1.91117632
H	1.01590895	2.96347882	1.74997274	H	3.01280100	1.82243375	2.74406131
H	-2.85079870	2.10530940	3.39191920	H	4.52278068	1.06098977	2.24054404
H	-0.81682901	3.54098842	3.29583165	C	1.15709498	2.63409558	-2.33055957
C	-0.06415480	-1.20122438	-0.95241855	H	0.19632309	2.63771220	-1.80136062
C	2.03905406	-1.86350953	-2.70954123	H	1.20641398	1.68789984	-2.88083208
C	1.11518455	-0.41551892	-0.97041563	H	1.15035010	3.45374651	-3.05630668
C	-0.13263881	-2.32721409	-1.78934338	S	-2.56049174	0.21904520	-1.69412644
C	0.90026527	-2.66246748	-2.65942998	S	2.56048871	-0.21917807	1.69441132
C	2.13894204	-0.76150706	-1.86589865	C	-3.73337033	1.31864770	-0.93515157
H	-1.01600514	-2.96326407	-1.75019807	C	-5.43278235	3.19090681	0.23836696
H	0.81687880	-3.54086616	-3.29582337	C	-4.89927154	0.89386261	-0.29564912
H	3.04069255	-0.15272714	-1.89617970	C	-3.41438668	2.67801093	-0.97944829
H	2.85086874	-2.10520693	-3.39181338	C	-4.26119836	3.60788705	-0.38807964
C	2.40503911	1.91867214	-0.24308269	C	-5.74729361	1.83579529	0.28018530
C	4.32185498	3.94362942	-0.68501112	H	-5.13844898	-0.16227267	-0.22279173
C	2.30170421	2.77160311	-1.36320558	H	-2.50509936	3.00500662	-1.47657744
C	3.46197379	2.14067744	0.65258845	H	-4.00516026	4.66303326	-0.42362046
C	4.41829757	3.13481783	0.44003923	H	-6.65556794	1.50136240	0.77425192
C	3.26336207	3.76333563	-1.57034409	H	-6.09726890	3.91959510	0.69407125
H	5.22989486	3.27628974	1.15149041	C	3.73329096	-1.31877382	0.93537694
H	3.17842833	4.40755586	-2.44333102	C	5.43262286	-3.19108874	-0.23823535
H	5.06351152	4.71652687	-0.86942228	C	4.89928466	-0.89404974	0.29599768
C	-2.40504681	-1.91858213	0.24300312	C	3.41418924	-2.67812453	0.97949352
C	-4.32141796	-3.94394523	0.68495346	C	4.26095162	-3.60801644	0.38808903
C	-3.46196856	-2.14080288	-0.65262844	C	5.74725519	-1.83600283	-0.27988468
C	-2.30147897	-2.77150435	1.36310326	H	5.13858302	0.16206618	0.22326243
C	-3.26293142	-3.76343298	1.57025502	H	2.50483183	-3.00509528	1.47651449
C	-4.41807911	-3.13515024	-0.44009169	H	4.00480250	-4.66314059	0.42350202
H	-3.17784030	-4.40764377	2.44323380	H	6.65559313	-1.50159630	-0.77385379
H	-5.22966851	-3.27679004	-1.15151756	H	6.09707755	-3.91979005	-0.69396489

Table S9. Cartesian Coordinates (Å) for *trans*-**1b** in the Gas Phase in S_0 Calculated at PBE0/6-31G(d) Level of Theory

atom	*x*	*y*	*z*	*atom*	*x*	*y*	*z*
B	0.84819844	1.27594702	-0.05946884	H	0.71389651	1.85612842	2.94578423
B	-0.83323351	-1.25819200	-0.00417367	H	-0.69890283	2.38166915	2.04144986
C	1.28543734	0.03408384	0.81902504	C	2.46055466	2.78913348	-2.01842108
C	2.22110389	-2.22793121	2.21596796	H	3.43368424	3.04202147	-2.45233582
C	2.44720850	0.08619462	1.60438505	H	1.69674153	3.09696406	-2.74333829
C	0.55515513	-1.18019405	0.76628618	C	-2.41873050	-2.78240209	1.94768428
C	1.05643168	-2.29560447	1.45661814	H	-1.65309661	-3.08984989	2.67072118
C	2.91402946	-1.02368062	2.30280987	H	-3.39163919	-3.02938438	2.38531299
H	3.00412543	1.01958625	1.66111693	C	-0.21793566	-2.70629240	-2.57885845
H	0.52500242	-3.24387332	1.38856790	H	0.70929392	-2.33820293	-2.12301679
H	3.82250954	-0.95421844	2.89656238	H	-0.71080035	-1.83738343	-3.02913728
H	2.58617247	-3.10949113	2.73816667	H	0.04967413	-3.40142345	-3.38097669
C	-0.53294050	1.19626362	-0.83813108	S	2.28156824	0.99049343	-1.93976141
C	-2.87988503	1.03680761	-2.39022352	S	-2.23698846	-0.98382130	1.85724971
C	-1.26611209	-0.01627166	-0.88776993	C	3.84476339	0.44271588	-1.31011935
C	-1.02605132	2.30922006	-1.53862131	C	6.20938986	-0.62254326	-0.29516388
C	-2.18422766	2.23982692	-2.30717985	C	4.79683008	1.28486995	-0.73222202
C	-2.42333170	-0.06943019	-1.67938715	C	4.08235714	-0.93343310	-1.37937964
H	-0.49181292	3.25602422	-1.47251465	C	5.25760418	-1.46330424	-0.86867626
H	-2.54191228	3.11825706	-2.83960000	C	5.97799595	0.74865810	-0.23308737
H	-2.98285117	-1.00147956	-1.73429994	H	4.61626756	2.35056998	-0.64200585
H	-3.78052348	0.96419903	-2.99544987	H	3.34323357	-1.58993094	-1.82964694
C	-1.40610386	-2.71950034	-0.33844476	H	5.43940665	-2.53177804	-0.92317714
C	-2.39003531	-5.31196955	-0.86845847	H	6.72083629	1.40312453	0.21209197
C	-1.09554095	-3.37062043	-1.55210903	C	-3.81743129	-0.43968978	1.26476069
C	-2.18027491	-3.41912877	0.60008152	C	-6.22637254	0.61184692	0.34523071
C	-2.67700671	-4.69801097	0.34400986	C	-4.76934853	-1.28169831	0.68625211
C	-1.59657755	-4.65068982	-1.80054379	C	-4.07132693	0.93092776	1.37047057
H	-3.28040160	-5.21104765	1.09065457	C	-5.26973850	1.45412276	0.90819575
H	-1.35799388	-5.13751355	-2.74398962	C	-5.97335580	-0.75277040	0.23645199
H	-2.77649055	-6.30439613	-1.08495412	H	-4.57046445	-2.34021200	0.55691405
C	1.43195917	2.73407369	0.26445048	H	-3.32735877	1.58837876	1.81128187
C	2.43308097	5.32021164	0.79525078	H	-5.46076043	2.51980153	0.98232647
C	2.21662077	3.42563653	-0.67151349	H	-6.71204792	-1.40529102	-0.21831972
C	1.12093748	3.39019497	1.47557340	C	7.45081699	-1.20170963	0.31494398
C	1.62992068	4.66690508	1.72450449	C	-7.54862487	1.16717942	-0.09454628
C	2.72165426	4.70113262	-0.41411444	F	7.83667738	-2.32515678	-0.31086266
H	1.39022670	5.15703645	2.66594078	F	8.47824854	-0.33806885	0.27072589
H	3.33332890	5.20784792	-1.15838169	F	7.26261415	-1.51939688	1.60861488
H	2.82614015	6.31007446	1.01159335	F	-8.07747535	0.45472289	-1.10247116
C	0.23358394	2.73396819	2.49929192	F	-7.44121475	2.43971383	-0.50830341
H	-0.02446088	3.42928644	3.30436163	F	-8.44729701	1.15556211	0.90619155

Table S10. Cartesian Coordinates (Å) for *trans*-**2a** in the Gas Phase in S_0 Calculated at PBE0/6-31G(d) Level of Theory

atom	*x*	*y*	*z*	*atom*	*x*	*y*	*z*
B	0.23344700	1.48805011	0.23107821	C	5.04170463	2.66550571	-2.58899681
B	-0.23293449	-1.48696583	-0.23284208	C	3.06428942	3.60099525	-1.55810002
C	1.43494310	0.45525677	0.31198685	C	2.90009034	1.60397364	-2.91503853
C	3.62239092	-1.31219354	0.21185919	C	4.27081741	1.64557923	-3.14036279
C	2.73052911	0.90779926	0.62418505	C	4.43406987	3.64099922	-1.80414833
C	1.23581753	-0.91154432	-0.01393949	H	2.61139426	4.34587544	-0.91164749
C	2.34814228	-1.77349301	-0.07798629	H	2.29837562	0.81101616	-3.35106375
C	3.81403240	0.04481653	0.58335488	H	4.73637306	0.87710554	-3.75071548
H	2.87828138	1.95335266	0.88842500	H	5.02973326	4.43743411	-1.36605187
H	2.20182515	-2.81272880	-0.37014056	H	6.11288656	2.69615614	-2.76563002
C	-1.23548996	0.91228285	0.01301507	C	-2.29499058	-2.58233316	2.12106721
C	-3.81387166	-0.04452375	-0.58303199	C	-5.04210622	-2.66721384	2.58760326
C	-1.43457507	-0.45455152	-0.31281684	C	-3.06390332	-3.60226761	1.55778206
C	-2.34790213	1.77403148	0.07756845	C	-2.90098767	-1.60487116	2.91428469
C	-3.62223785	1.31251238	-0.21166844	C	-4.27178981	-1.64689716	3.13904602
C	-2.73024889	-0.90732977	-0.62437022	C	-4.43377982	-3.64266295	1.80323538
H	-2.20164330	2.81330685	0.36962478	H	-2.61050756	-4.34716452	0.91170690
H	-2.87794800	-1.95292368	-0.88847805	H	-2.29974802	-0.81156977	3.35034789
C	-0.34600299	-2.90177720	-0.98799116	H	-4.73786478	-0.87842421	3.74900517
C	-0.47943208	-5.41686380	-2.26315307	H	-5.02898241	-4.43940780	1.36506883
C	-0.45073649	-2.98866249	-2.39326619	H	-6.11335610	-2.69818463	2.76376843
C	-0.27183197	-4.10038539	-0.26184930	H	5.61198108	2.29959866	1.40732152
C	-0.34304404	-5.34852940	-0.88251868	C	6.03388267	1.31516854	1.22115889
C	-0.52307124	-4.24152859	-3.00692961	C	5.24664613	0.25108933	0.84145136
H	-0.28930715	-6.25960958	-0.28930436	C	8.03164448	-0.11165683	1.14271681
H	-0.61368090	-4.29490458	-4.09005551	C	5.87282647	-0.99663561	0.61252263
H	-0.54480747	-6.38129092	-2.76016579	C	7.43070788	1.11132704	1.36821391
C	0.34630271	2.90253404	0.98709409	C	7.24641254	-1.22724823	0.74693418
C	0.47920958	5.41758745	2.26233574	C	4.93235392	-1.98031573	0.22519932
C	0.27205812	4.10117810	0.26102718	H	8.04795451	1.95349440	1.67102421
C	0.45078569	2.98933604	2.39237114	H	8.74410354	-2.79625864	0.55534442
C	0.52290664	4.24221140	3.00605887	H	9.10631653	-0.22185915	1.26784738
C	0.34297526	5.34933904	0.88167577	C	5.39123045	-3.25151840	-0.03918940
H	0.61338657	4.29556096	4.08919706	H	4.71991613	-4.05063119	-0.34426853
H	0.28916404	6.26043769	0.28850089	C	6.77967900	-3.51617259	0.08974045
H	0.54440357	6.38198838	2.75942269	H	7.13675325	-4.52131759	-0.11987669
C	0.47103278	1.74607187	3.24029081	C	7.68874704	-2.54824616	0.46928723
H	-0.36897886	1.08735498	2.98859080	H	-5.61192414	-2.29955759	-1.40614274
H	0.40537556	1.99116908	4.30520043	C	-6.03384949	-1.31515909	-1.21987501
H	1.38425121	1.16239261	3.07885222	C	-5.24657357	-0.25099049	-0.84050967
C	0.04416433	4.01473482	-1.22871736	C	-8.03174513	0.11144429	-1.14065736
H	0.55460647	4.79750140	-1.79933696	C	-5.87280525	0.99666730	-0.61135751

H	-1.02360124	4.08143190	-1.47125910	C	-7.43075948	-1.11147343	-1.36636802
C	-0.04372189	-4.01433980	1.22790456	C	-7.24646964	1.22712801	-0.74521544
H	1.02415351	-4.08050607	1.47010417	C	-4.93228430	1.98046990	-0.22445823
H	-0.55345563	-4.79787627	1.79810745	H	-8.04803109	-1.95372133	-1.66890405
C	-0.47111822	-1.74542340	-3.24123822	H	-8.74425366	2.79597487	-0.55304877
H	0.36855038	-1.08636140	-2.98928437	H	-9.10648025	0.22152618	-1.26535007
H	-1.38462571	-1.16209162	-3.08016056	C	-5.39119119	3.25163173	0.04008674
H	-0.40499533	-1.99053835	-4.30611570	H	-4.71983821	4.05082506	0.34487094
S	0.54767402	2.39257144	-1.85516269	C	-6.77971991	3.51612733	-0.08827309
S	-0.54782360	-2.39297120	1.85579845	H	-7.13682348	4.52123567	0.12146992
C	2.29483008	2.58145306	-2.12130112	C	-7.68883319	2.54808409	-0.46742187

Table S11. Cartesian Coordinates (Å) for *trans*-**2b** in the Gas Phase in S_0 Calculated at PBE0/6-31G(d) Level of Theory

atom	*x*	*y*	*z*	*atom*	*x*	*y*	*z*
B	-0.48424815	-1.35414135	0.46900708	C	-5.06705741	-1.01823161	-2.10842712
B	0.48560889	1.35603490	-0.46708933	C	-5.29627399	-2.97094302	-0.71431951
C	-1.43972684	-0.09914840	0.52554546	H	-3.49122482	-3.99514465	-0.19565148
C	-3.22096027	2.07623313	0.41277593	H	-3.06956907	-0.52933984	-2.72057013
C	-2.75548699	-0.23331770	1.00526836	H	-5.51080668	-0.16974608	-2.61829807
C	-1.00918475	1.15373282	0.01164451	H	-5.92172879	-3.64685521	-0.13866620
C	-1.92222127	2.22386633	-0.04871982	C	3.11434580	2.29796300	1.48677551
C	-3.64196827	0.83185781	0.95360671	C	5.87065134	1.89087923	1.38341600
H	-3.08145113	-1.19358965	1.40117369	C	3.92315448	3.17348790	0.75726555
H	-1.60615056	3.17155563	-0.48237388	C	3.69411269	1.22101230	2.16454379
C	1.01015703	-1.15222464	-0.01043341	C	5.06572863	1.01747714	2.11013083
C	3.64241851	-0.83105765	-0.95393758	C	5.29642285	2.96885244	0.71435212
C	1.44071219	0.10056658	-0.52452490	H	3.49201957	3.99346160	0.19410180
C	1.92300910	-2.22259053	0.04952369	H	3.06776027	0.53020000	2.72207066
C	3.22149971	-2.07530045	-0.41270952	H	5.50889116	0.16923100	2.62090722
C	2.75616069	0.23433508	-1.00514658	H	5.92242773	3.64389355	0.13827907
H	1.60691686	-3.17015976	0.48341416	C	-7.35862325	-1.70723395	-1.33204845
H	3.08212619	1.19446770	-1.40138651	C	7.35789801	1.70458736	1.33388152
C	0.80624487	2.66399643	-1.33428179	F	-7.99014137	-2.54867627	-2.17200148
C	1.29269962	4.99246806	-2.85565067	F	-7.84337057	-1.95873717	-0.10204309
C	0.77006625	2.62121215	-2.74535191	F	-7.72523508	-0.46398393	-1.66826117
C	1.05386023	3.90352059	-0.72205961	F	7.84319827	1.95493679	0.10388454
C	1.30207829	5.05675965	-1.46802660	F	7.72369930	0.46138873	1.67114835
C	1.01954383	3.78148615	-3.48261666	F	7.98949641	2.54630563	2.17347859
H	1.49817816	6.00134055	-0.96369555	H	-5.78111947	-0.92115129	2.11830412
H	0.99708227	3.73187706	-4.56931989	C	-6.00759415	0.11136950	1.86566518
H	1.49163860	5.88248236	-3.44680284	C	-5.05652799	0.95382316	1.33424004
C	-0.80553815	-2.66269650	1.33487589	C	-7.67926000	1.90688629	1.76407858
C	-1.29286072	-4.99163198	2.85533581	C	-5.43783705	2.27767171	1.01409411

C	-1.05319029	-3.90195973	0.72209491	C	-7.32002480	0.60955743	2.07239797
C	-0.76985000	-2.62046353	2.74600565	C	-6.72210515	2.79785478	1.20976817
C	-1.01971417	-3.78092794	3.48281938	C	-4.36115136	3.00289630	0.45268672
C	-1.30184405	-5.05536245	1.46769527	H	-8.06891263	-0.06202189	2.48330342
H	-0.99758350	-3.73168585	4.56954556	H	-7.88879885	4.61647939	0.94537729
H	-1.49798593	-5.99972238	0.96296159	H	-8.69805813	2.24504844	1.93750366
H	-1.49211338	-5.88184232	3.44608703	C	-4.57923760	4.31059921	0.07905890
C	-0.45017795	-1.33989264	3.47072515	H	-3.79349303	4.91918939	-0.36199120
H	-1.24852316	-0.59868729	3.35226710	C	-5.86900411	4.86869472	0.27416794
H	0.46423823	-0.88022091	3.07642172	H	-6.03678188	5.90115925	-0.02137720
H	-0.30600997	-1.51662951	4.54125094	C	-6.91500608	4.14934471	0.81886223
C	-0.98764924	-3.99762702	-0.78352717	H	5.78111904	0.92138870	-2.12025158
H	-1.63896153	-4.77570268	-1.19549484	C	6.00758156	-0.11113639	-1.86761142
H	0.03185368	-4.22512561	-1.11948307	C	5.05670241	-0.95335202	-1.33546519
C	0.98862945	3.99909228	0.78355684	C	7.67895292	-1.90697192	-1.76691535
H	-0.03056268	4.22755865	1.11979770	C	5.43795543	-2.27724319	-1.01542330
H	1.64097174	4.77612506	1.19585351	C	7.31976947	-0.60960771	-2.07516499
C	0.45037375	1.34032193	-3.46946871	C	6.72199799	-2.79769156	-1.21186574
H	-0.46348804	0.88034573	-3.07426162	C	4.36148653	-3.00219915	-0.45325526
H	1.24915911	0.59951296	-3.35145978	H	8.06850940	0.06173827	-2.48672618
H	0.30526780	1.51670812	-4.53992590	H	7.88852018	-4.61651187	-0.94803192
S	-1.34985881	-2.41419860	-1.57759480	H	8.69756499	-2.24535849	-1.94099518
S	1.34937740	2.41466786	1.57637266	C	4.57956844	-4.30989845	-0.07962501
C	-3.11473806	-2.29827821	-1.48689111	H	3.79399010	-4.91829706	0.36198492
C	-5.87131646	-1.89269914	-1.38224951	C	5.86910900	-4.86826137	-0.27549601
C	-3.92291870	-3.17483568	-0.75788414	H	6.03687364	-5.90072617	0.02005489
C	-3.69536016	-1.22099880	-2.16345648	C	6.91489993	-4.14917692	-0.82093575

Table S12. Cartesian Coordinates (Å) for *cis*-**1a** in the Gas Phase in S_1 Calculated at PBE0/6-31G(d) Level of Theory

atom	*x*	*y*	*z*	*atom*	*x*	*y*	*z*
B	-1.32741262	1.99178403	-0.31007048	H	6.76178799	0.41627974	1.68854749
B	1.52817372	1.64985127	0.50806106	C	-2.53148465	-0.55794612	-1.18106257
C	1.14828538	1.77841656	-0.98469739	H	-1.48621870	-0.30775223	-1.37905187
C	0.46550134	1.99016359	-3.72942745	H	-2.85721302	-1.31007977	-1.90036147
C	2.12411630	1.70313708	-2.00328374	C	-3.17171100	4.38475471	-0.43603893
C	-0.22342810	1.95054253	-1.37658332	H	-2.31240075	4.63860968	-1.06678767
C	-0.51797899	2.04952994	-2.75964334	H	-2.81459539	4.40462418	0.59948647
C	1.80528159	1.81140316	-3.34631372	H	-3.93024951	5.16507446	-0.55532918
H	3.16433602	1.55617564	-1.71563584	C	3.70035418	3.70376021	0.44191326
H	-1.55383228	2.18770871	-3.06836683	H	4.56376630	4.36710663	0.56148598
H	2.58605228	1.76044637	-4.10231090	H	2.88259628	4.09290384	1.05962358
H	0.20632461	2.08169406	-4.78210907	H	3.36303916	3.77033657	-0.59902690
C	0.42196029	1.81801411	1.56997960	C	2.36658142	-1.05467902	1.59217523

C	-1.59426896	2.10561255	3.54167830	H	2.78294065	-1.84347906	2.22718831
C	-0.95563370	1.96575457	1.17731847	H	1.48967805	-0.64103958	2.10056674
C	0.72650748	1.83149878	2.95029642	S	-2.51718527	-1.25903956	0.49911554
C	-0.24710826	1.97606274	3.92335812	S	1.63129496	-1.86431681	0.11088424
C	-1.92886381	2.09309316	2.20049182	C	-3.52451648	-2.65419859	0.52121609
H	1.76878435	1.73068406	3.25116327	C	-5.07168706	-4.94701567	0.86764343
H	0.02577335	1.99284512	4.97624861	C	-4.10526743	-3.28875013	-0.59736119
H	-2.97658199	2.19799254	1.91901420	C	-3.73339006	-3.18964300	1.81734665
H	-2.36633279	2.22264055	4.29947903	C	-4.49977416	-4.32707134	1.98107453
C	-2.86216321	1.90377007	-0.75854857	C	-4.87108059	-4.42416369	-0.41505822
C	-5.58187587	1.64516575	-1.52530438	H	-3.95415127	-2.90048843	-1.59662162
C	-3.41539660	0.66654253	-1.14991818	H	-3.28647546	-2.69578096	2.67500882
C	-3.71304649	3.02797175	-0.79558699	H	-4.65694865	-4.73401490	2.97433992
C	-5.05084541	2.88446601	-1.17266785	H	-5.31586762	-4.91417838	-1.27501516
C	-4.76178776	0.52813434	-1.50708824	H	-5.67473235	-5.84023788	0.99616570
H	-5.69204041	3.76350834	-1.19161506	C	3.06049946	-2.60443254	-0.64794677
H	-5.15894374	-0.44436072	-1.79351457	C	5.27907720	-3.76988671	-1.88105057
H	-6.62434680	1.55837326	-1.81899235	C	3.63963396	-2.00026001	-1.76663437
C	3.02828546	1.30973102	0.91809997	C	3.59579358	-3.79768212	-0.15319829
C	5.72379454	0.66488964	1.48154829	C	4.70519857	-4.37384405	-0.76409111
C	3.39591892	0.01534914	1.34689391	C	4.74327924	-2.58651967	-2.38147846
C	4.04335800	2.28688915	0.82542919	H	3.22042875	-1.07136638	-2.14238194
C	5.37163005	1.95565636	1.10584614	H	3.13470973	-4.27120065	0.70962709
C	4.73266762	-0.29935502	1.60896918	H	5.11824638	-5.29992648	-0.37211559
H	6.13808249	2.72516202	1.03077190	H	5.19049833	-2.11041245	-3.25018803
H	4.99265388	-1.30893053	1.92144830	H	6.14317545	-4.22256282	-2.36046071

Table S13. Cartesian Coordinates (Å) for *cis*-**1b** in the Gas Phase in S_1 Calculated at PBE0/6-31G(d) Level of Theory

atom	*x*	*y*	*z*	*atom*	*x*	*y*	*z*
B	-1.71796930	2.31098163	-0.64348178	H	-4.86242882	4.88584499	-0.79435181
B	0.93225949	2.73470341	0.66545823	H	-3.10818707	4.75125004	-1.02563666
C	-0.30456130	2.44233105	1.52527508	C	2.39402327	5.25293370	1.47319296
C	-2.58255535	1.84353033	3.09595673	H	2.19206605	5.40186607	0.40710450
C	-1.57794397	2.23204885	0.89406183	H	3.01790372	6.07921814	1.82795936
C	-0.23274739	2.34545301	2.93814808	H	1.42677151	5.30439691	1.98481136
C	-1.33984116	2.06002414	3.71476798	C	-2.21325662	-0.23946333	-2.20990004
C	-2.68379686	1.92580482	1.71713209	H	-2.48036249	-0.96469135	-2.98527741
H	0.72409840	2.51203529	3.43227729	H	-1.29243958	0.26137952	-2.52735292
H	-1.25238492	2.00459963	4.79769031	C	2.31602542	0.21896905	1.36446648
H	-3.64843935	1.74762700	1.24423091	H	2.61834726	-0.60730716	2.00789423
H	-3.45961390	1.61793512	3.69888837	H	1.22945317	0.32026433	1.36447968
C	0.79809901	2.90221620	-0.85083565	S	-1.66250826	-1.16392095	-0.71793021
C	0.51189441	3.27402617	-3.64727491	S	2.80583282	-0.08161240	-0.36299211

C	1.89404079	3.26841486	-1.67299326	C	-3.14818725	-1.99440284	-0.21303416
C	-0.48293476	2.70853486	-1.47908040	C	-5.42407098	-3.34565921	0.67233742
C	-0.57651384	2.90631364	-2.87522506	C	-3.77751046	-2.93390375	-1.03682731
C	1.76463684	3.45690413	-3.03597772	C	-3.66821598	-1.73974894	1.05840344
H	2.87069253	3.41992605	-1.21347612	C	-4.79726260	-2.42008325	1.50202307
H	-1.54620446	2.77151786	-3.35290986	C	-4.91590528	-3.59797813	-0.60179383
H	2.62406018	3.75342891	-3.63370772	H	-3.36413411	-3.15341662	-2.01717290
H	0.40003658	3.42680412	-4.71828517	H	-3.19114845	-0.99514501	1.68908285
C	2.37866560	2.73680289	1.35336504	H	-5.19607521	-2.22445765	2.49253767
C	4.96059486	2.63678075	2.54134902	H	-5.39953510	-4.33019209	-1.24125444
C	3.02683049	1.51205457	1.65886688	C	4.12786855	-1.21536751	-0.32078235
C	3.04910988	3.92124742	1.71377232	C	6.23670822	-3.01619522	-0.53375691
C	4.32049282	3.85644672	2.29189455	C	4.99291075	-1.16484341	-1.43034135
C	4.31944337	1.45950265	2.21224861	C	4.32152101	-2.18930208	0.67330479
H	4.83021235	4.78241729	2.54953721	C	5.37340422	-3.08184888	0.56202786
H	4.79327654	0.49972139	2.40784210	C	6.04258462	-2.06206470	-1.53170524
H	5.94705638	2.62080628	2.99544802	H	4.83755490	-0.41474783	-2.19992382
C	-3.10995489	1.97635533	-1.33867666	H	3.64364073	-2.27047068	1.51518440
C	-5.62773850	1.34752044	-2.46332759	H	5.51925986	-3.84529318	1.31880831
C	-3.32564942	0.75400053	-2.01119069	H	6.71109593	-2.02474889	-2.38489758
C	-4.18126546	2.89402037	-1.27935507	C	-6.67697766	-4.03477302	1.12241322
C	-5.42085083	2.57070237	-1.83765761	C	7.41337632	-3.95450095	-0.60978292
C	-4.57601894	0.44529409	-2.55445423	F	-7.78014823	-3.36835447	0.73752614
H	-6.23281532	3.29354883	-1.78233479	F	-6.77550300	-5.27423468	0.60988537
H	-4.72071564	-0.50818869	-3.05876483	F	-6.73571749	-4.14637617	2.45996794
H	-6.59833853	1.10425878	-2.88812952	F	7.77943728	-4.17783909	-1.87703770
C	-3.99323343	4.24048669	-0.62861514	F	7.13189165	-5.13410118	-0.04311804
H	-3.84112887	4.14793368	0.45312040	F	8.47030167	-3.43904100	0.03354712

Table S14. Cartesian Coordinates (Å) for *cis*-**2a** in the Gas Phase in S_1 Calculated at PBE0/6-31G(d) Level of Theory

atom	*x*	*y*	*z*	*atom*	*x*	*y*	*z*
B	-0.55425697	-1.35218123	-1.08354961	C	-5.16998518	-3.55467373	3.38384278
B	0.29517945	1.51558255	-1.04698546	C	-2.76002161	-3.65745942	3.48724691
C	1.35164169	0.40385250	-1.15398165	C	-3.84148241	-1.87206878	2.27297395
C	3.29071758	-1.65330497	-1.20817658	C	-5.08535516	-2.39427746	2.62021851
C	2.73542390	0.71749911	-1.19098200	C	-4.00419863	-4.18693622	3.81272861
C	0.94965329	-0.97840462	-1.15038216	H	-1.85283397	-4.13658575	3.84616081
C	1.94290150	-1.97773335	-1.19448258	H	-3.77223587	-0.98219936	1.65454711
C	3.69360697	-0.27806603	-1.20355397	H	-5.98720485	-1.89504699	2.27527925
H	3.03912705	1.76452325	-1.20250432	H	-4.06387973	-5.08990248	4.41517798
H	1.63379105	-3.02262514	-1.20800616	H	-6.14081098	-3.96675867	3.64618220
C	-1.19615010	1.16049612	-1.03958326	C	3.43429960	2.02416229	2.48090533
C	-3.93277910	0.47613104	-0.94249917	C	5.86588789	3.02426291	3.38952990

C	-1.60419760	-0.21940253	-1.06882291	C	3.48478736	3.29072705	3.09634017
C	-2.18627446	2.17173839	-0.96462473	C	4.61795102	1.26328674	2.32686608
C	-3.52796718	1.85199996	-0.92291361	C	5.82341158	1.77120748	2.77548193
C	-2.98143002	-0.52394277	-1.01497307	C	4.69643463	3.77658966	3.55001796
H	-1.87687471	3.21620377	-0.93761919	H	2.58199083	3.87236901	3.22958094
H	-3.28895628	-1.56860993	-1.01162636	H	4.57598134	0.30062570	1.82675223
C	-1.01579330	-2.87209664	-1.02679531	H	6.73225763	1.19573161	2.63540500
C	-1.97429350	-5.52879796	-0.93718580	H	4.73838032	4.74649617	4.03524563
C	-0.91797810	-3.64203219	0.15276284	H	6.81215598	3.42031841	3.74490054
C	-1.57128094	-3.48180958	-2.17306960	H	-6.07468139	-1.61782916	-0.85689868
C	-2.04114780	-4.79633403	-2.11576615	C	-6.34713825	-0.56620264	-0.81319059
C	-1.40695908	-4.95098991	0.19093972	C	-5.39304844	0.43206292	-0.85351791
H	-2.46355468	-5.24859700	-3.01116406	C	-8.12635297	1.12062015	-0.65706064
H	-1.33591815	-5.52087666	1.11513707	C	-5.82812009	1.77828473	-0.79107759
H	-2.35163207	-6.54766071	-0.90094967	C	-7.71145197	-0.19734582	-0.71439789
C	0.76253285	3.01649568	-0.76945145	C	-7.16998167	2.16984322	-0.69458095
C	1.48910460	5.69247736	-0.22106462	C	-4.73525554	2.67766515	-0.83351402
C	0.94894643	3.47196292	0.55107563	H	-8.45988365	-0.98561501	-0.68585612
C	0.96844582	3.93766289	-1.81729580	H	-8.42975728	3.94064304	-0.57068486
C	1.33407040	5.25503128	-1.53234636	H	-9.18565951	1.35533681	-0.58324759
C	1.29867917	4.79717526	0.82290657	C	-5.00263481	4.03100720	-0.78192760
H	1.48499091	5.95367247	-2.35273399	H	-4.20748540	4.77188596	-0.81294327
H	1.38755982	5.14488565	1.85018304	C	-6.35082201	4.45525175	-0.68805110
H	1.74818909	6.72730082	-0.01437308	H	-6.55655194	5.52223267	-0.64928583
C	-0.24628906	-3.11449168	1.39306668	C	-7.41059941	3.56824937	-0.64386093
H	0.71838955	-2.65534704	1.14997384	H	5.84548520	1.81602028	-1.19819398
H	-0.04874421	-3.93398691	2.09158503	C	6.11642358	0.76299693	-1.18537099
C	-1.65152825	-2.72564044	-3.47466913	C	5.15858556	-0.23369255	-1.19916779
H	-0.68350835	-2.28347007	-3.73845777	C	7.89754297	-0.92829938	-1.16827874
H	-2.36794844	-1.89784272	-3.41662425	C	5.59409897	-1.58093285	-1.19675427
H	-1.95949735	-3.38170194	-4.29560983	C	7.48343230	0.39164049	-1.17007795
C	0.77539106	3.49697919	-3.24322080	C	6.93847567	-1.97573040	-1.18312934
H	1.42714561	2.64978836	-3.48627027	C	4.49762109	-2.47828653	-1.20978297
H	0.98874555	4.30728525	-3.94750775	H	8.23437620	1.17816417	-1.16875150
H	-0.25344829	3.15834273	-3.41167770	H	8.19984807	-3.75024552	-1.17923606
C	0.71277114	2.52940024	1.70785878	H	8.95885107	-1.16562743	-1.16205922
H	0.59981347	3.04992997	2.66418443	C	4.76588025	-3.83417560	-1.21278459
H	-0.18049210	1.91515453	1.55236862	H	3.96894573	-4.57326609	-1.22591894
S	-1.10249314	-1.76890401	2.30245968	C	6.11577497	-4.26002187	-1.20168192
S	2.00698377	1.24783602	1.90575708	H	6.32119168	-5.32756620	-1.20663758
C	-2.67191210	-2.50111473	2.70600486	C	7.17924841	-3.37507338	-1.18615517

Table S15. Cartesian Coordinates (Å) for *cis*-**2b** in the Gas Phase in S_1 Calculated at PBE0/6-31G(d) Level of Theory

atom	*x*	*y*	*z*	*atom*	*x*	*y*	*z*
B	-0.71840808	-1.33716431	-1.08891594	C	-5.40479602	-0.52751237	2.32082362
B	0.71834696	1.33728689	-1.08880239	C	-5.45443094	-2.92477512	2.07399050
C	1.51508698	0.01756620	-1.08216152	H	-3.56191935	-3.90675870	1.91761082
C	2.96769487	-2.40797893	-1.14127379	H	-3.46141374	0.37843414	2.31111126
C	2.92663831	0.04196409	-1.05129343	H	-5.92548304	0.41808393	2.43179245
C	0.82403959	-1.26813063	-1.10961759	H	-6.01542730	-3.85180806	2.00498993
C	1.58529376	-2.45122938	-1.16079531	C	3.33473682	1.76482536	2.10003403
C	3.65509900	-1.12885288	-1.06309034	C	6.12952929	1.71443096	2.21482337
H	3.43414105	1.00328243	-1.00666660	C	4.06693681	2.95460486	2.02586980
H	1.06993680	-3.40872548	-1.21297072	C	4.02044092	0.55138971	2.25843326
C	-0.82407853	1.26823802	-1.10947977	C	5.40490328	0.52736802	2.32120377
C	-3.65513861	1.12895543	-1.06299408	C	5.45472855	2.92452154	2.07342243
C	-1.51511726	-0.01745858	-1.08214728	H	3.56232341	3.90657936	1.91638690
C	-1.58535897	2.45135042	-1.16046297	H	3.46146362	-0.37844309	2.31183353
C	-2.96774808	2.40808816	-1.14095545	H	5.92550712	-0.41822878	2.43256133
C	-2.92666677	-0.04185895	-1.05130546	H	6.01580196	3.85148156	2.00405349
H	-1.07001029	3.40885837	-1.21250867	H	-6.17962047	-0.46264910	-0.79780971
H	-3.43416862	-1.00318257	-1.00677877	C	-6.22399124	0.61940469	-0.87402312
C	-1.44829019	-2.74667824	-1.17312496	C	-5.08440096	1.39180151	-1.00360444
C	-2.66645208	-5.28381925	-1.43042786	C	-7.61123802	2.64665006	-0.89641082
C	-1.57979113	-3.60620674	-0.06327940	C	-5.22891850	2.80143206	-1.06620723
C	-1.94191604	-3.19234591	-2.41990384	C	-7.47825509	1.27069182	-0.81903599
C	-2.54611866	-4.44683777	-2.53241532	C	-6.46153208	3.46950118	-1.02320974
C	-2.18113862	-4.86088644	-0.20015039	C	-3.97051505	3.45557326	-1.15670808
H	-2.91838564	-4.77195916	-3.50173863	H	-8.36768341	0.65926328	-0.69714301
H	-2.25854909	-5.51715236	0.66551965	H	-7.32026446	5.46562240	-1.07278855
H	-3.13236135	-6.26070588	-1.52976088	H	-8.59819402	3.10007524	-0.84921420
C	1.44818236	2.74682558	-1.17309110	C	-3.94950484	4.83835983	-1.22291349
C	2.66597008	5.28412229	-1.43066756	H	-3.01740861	5.39222959	-1.29460859
C	1.57976710	3.60640011	-0.06330510	C	-5.17885056	5.53115376	-1.19530860
C	1.94161029	3.19249540	-2.41995603	H	-5.16428444	6.61646671	-1.24965399
C	2.54562456	4.44706060	-2.53259908	C	-6.40187592	4.88414405	-1.09671665
C	2.18089958	4.86117326	-0.20030373	H	6.17960332	0.46265513	-0.79744181
H	2.91775168	4.77218590	-3.50197493	C	6.22394843	-0.61938433	-0.87385537
H	2.25837976	5.51745547	0.66534908	C	5.08435719	-1.39173061	-1.00367412
H	3.13173722	6.26106513	-1.53011689	C	7.61116362	-2.64665079	-0.89649814
C	-1.02980162	-3.24037234	1.29448217	C	5.22885195	-2.80135439	-1.06649452
H	0.05559514	-3.08700060	1.24445679	C	7.47820048	-1.27070957	-0.81888836
H	-1.20010101	-4.05104239	2.01115792	C	6.46144657	-3.46945731	-1.02351973
C	-1.80698264	-2.32800797	-3.64693062	C	3.97043210	-3.45546156	-1.15714876
H	-2.12206827	-2.86556281	-4.54699208	H	8.36762181	-0.65931460	-0.69679585
H	-0.77054656	-2.00123772	-3.79275882	H	7.32012942	-5.46559007	-1.07335364

H	-2.41505782	-1.41901899	-3.56782358	H	8.59810443	-3.10010909	-0.84931133
C	1.80665638	2.32806546	-3.64691089	C	3.94939497	-4.83824465	-1.22355729
H	2.41468144	1.41904923	-3.56772410	H	3.01728872	-5.39208058	-1.29537337
H	2.12177686	2.86553261	-4.54701187	C	5.17871962	-5.53106046	-1.19599570
H	0.77020331	2.00133903	-3.79272168	H	5.16414347	-6.61636463	-1.25050359
C	1.03010912	3.24050974	1.29459912	C	6.40175775	-4.88408305	-1.09724274
H	1.20081099	4.05105099	2.01132838	C	-7.62382985	-1.68056044	2.15957845
H	-0.05534126	3.08741260	1.24488500	C	7.62403750	1.68021775	2.15977163
S	-1.57798554	-1.66226188	2.02834816	F	-8.17409285	-2.79754763	2.66150988
S	1.57820485	1.66223752	2.02807146	F	-8.13288550	-0.63710375	2.83175191
C	-3.33453412	-1.76490257	2.10022944	F	-8.07059124	-1.57437892	0.88591814
C	-6.12932138	-1.71466963	2.21486579	F	8.17429929	2.79722409	2.66163537
C	-4.06662979	-2.95477154	2.02656399	F	8.07098690	1.57387833	0.88614514
C	-4.02032753	-0.55145776	2.25810516	F	8.13293281	0.63681442	2.83209981

Table S16. Cartesian Coordinates (Å) for *cis*-**5** in the Gas Phase in S_0 Calculated at PBE0/6-31G(d) Level of Theory

atom	*x*	*y*	*z*	*atom*	*x*	*y*	*z*
B	-0.00037897	1.52045043	-0.00998394	C	-0.16731154	5.86494760	-0.88473069
B	0.00037897	-1.52045043	-0.00998394	C	-0.35735286	4.01350809	0.62972839
C	1.35289300	0.71218117	-0.28018798	C	0.15349096	3.58817998	-1.68646481
C	3.75221397	-0.69222859	-0.75792012	C	0.09728232	4.96589405	-1.91402391
C	2.56225614	1.38310598	-0.51921452	C	-0.40997186	5.38753971	0.39674373
C	1.35460166	-0.70458633	-0.28155658	H	0.26071244	5.34039377	-2.92270393
C	2.56225614	-1.37674395	-0.52489604	H	-0.64631636	6.07572938	1.20608887
C	3.75241511	0.69927726	-0.75475835	H	-0.19634548	6.93266166	-1.08631339
H	2.56704807	2.47270068	-0.53845032	C	0.40224581	2.65708373	-2.84260597
H	2.56598460	-2.46558554	-0.55026457	H	-0.30998386	1.82457932	-2.83693042
H	4.67166743	1.24958130	-0.94370113	H	1.40314115	2.21333055	-2.79670785
H	4.67136986	-1.24151452	-0.94986653	H	0.30711204	3.18333889	-3.79795549
C	-1.35460166	0.70458633	-0.28155658	C	-0.73120335	3.44995171	1.97718692
C	-3.75241511	-0.69927726	-0.75475835	H	-1.81778464	3.33455824	2.07448297
C	-1.35289300	-0.71218117	-0.28018798	H	-0.38656649	4.05288419	2.82441793
C	-2.56225614	1.37674395	-0.52489604	C	0.73120335	-3.44995171	1.97718692
C	-3.75221397	0.69222859	-0.75792012	H	1.81778464	-3.33455824	2.07448297
C	-2.56225614	-1.38310598	-0.51921452	H	0.38656649	-4.05288419	2.82441793
H	-2.56598460	2.46558554	-0.55026457	C	-0.40224581	-2.65708373	-2.84260597
H	-4.67136986	1.24151452	-0.94986653	H	0.30998386	-1.82457932	-2.83693042
H	-2.56704807	-2.47270068	-0.53845032	H	-1.40314115	-2.21333055	-2.79670785
H	-4.67166743	-1.24958130	-0.94370113	H	-0.30711204	-3.18333889	-3.79795549
C	0.04426634	-3.09131482	-0.37968715	S	-0.05236867	1.77325093	2.11965304
C	0.16731154	-5.86494760	-0.88473069	S	0.05236867	-1.77325093	2.11965304
C	-0.15349096	-3.58817998	-1.68646481	C	1.65150579	2.16528768	2.57064714
C	0.35735286	-4.01350809	0.62972839	H	1.68322233	2.51303719	3.60633403

C	0.40997186	-5.38753971	0.39674373	H	2.05009337	2.92318676	1.89241655
C	-0.09728232	-4.96589405	-1.91402391	H	2.23602199	1.24955013	2.46506383
H	0.64631636	-6.07572938	1.20608887	C	-1.65150579	-2.16528768	2.57064714
H	-0.26071244	-5.34039377	-2.92270393	H	-2.23602199	-1.24955013	2.46506383
H	0.19634548	-6.93266166	-1.08631339	H	-1.68322233	-2.51303719	3.60633403
C	-0.04426634	3.09131482	-0.37968715	H	-2.05009337	-2.92318676	1.89241655

Table S17. Cartesian Coordinates (Å) for **A** in the Gas Phase in S_0 Calculated at PBE0/6-31G(d) Level of Theory

atom	*x*	*y*	*z*	*atom*	*x*	*y*	*z*
B	-0.18126536	-0.64461925	-0.22248647	C	-2.20622892	0.64045128	-0.77357626
C	1.40662620	-0.51215146	0.00829587	C	-3.06870194	1.47015515	-1.48662357
C	4.22066904	-0.44514553	0.18716523	C	-1.36586089	1.74015434	-3.18569196
C	2.04406632	0.38139834	0.88769461	H	0.49744122	0.71681356	-2.84907263
C	2.22017437	-1.33887857	-0.78932173	H	-4.06209484	1.70110155	-1.10818201
C	3.60827753	-1.32040066	-0.70583892	H	-1.04049018	2.18066060	-4.12496523
C	3.44150351	0.39884952	0.96750037	H	-3.30719696	2.66600208	-3.25896507
H	4.20860696	-1.97955407	-1.32981663	C	1.50832448	-2.22613983	-1.78007401
H	3.91812548	1.10097485	1.64753573	H	1.97895741	-3.20472956	-1.92195960
H	5.30380413	-0.42181372	0.27281543	H	1.41561648	-1.75578402	-2.76658895
C	-1.29100509	-0.73716840	0.93688026	C	1.24956354	1.32286496	1.76147109
C	-3.56845422	-0.59331557	2.56784010	H	1.42344566	1.10195391	2.82170914
C	-2.43336562	-0.00818783	0.52983464	H	0.17735395	1.20864073	1.58624585
C	-1.31091346	-1.36786392	2.17972580	S	-0.19663410	-2.45470210	-1.19123331
C	-2.44427780	-1.29841507	2.99365419	S	1.69270882	3.08405475	1.59791766
C	-3.56727263	0.05960446	1.33645852	C	0.10467146	-3.66022813	0.12071110
H	-0.43203280	-1.90295586	2.53662613	H	-0.80286489	-3.71660773	0.72479344
H	-2.44835508	-1.78734212	3.96478051	H	0.94157608	-3.33021778	0.74145884
H	-4.44352578	0.62080809	1.01955303	H	0.31449786	-4.63509684	-0.32611891
H	-4.44792633	-0.54162550	3.20483822	C	1.23465478	3.36320653	-0.12903509
C	-0.91362702	0.34474591	-1.26084138	H	1.81972483	2.72703852	-0.79933057
C	-2.64202859	2.01500679	-2.69706175	H	1.45367699	4.41078148	-0.35255672
C	-0.50487814	0.90751836	-2.46653512	H	0.16928959	3.17804156	-0.29310907

Table S18. Cartesian Coordinates (Å) for Phenylmethylsulfide in the Gas Phase in S_0 Calculated at PBE0/6-31G(d) Level of Theory

atom	*x*	*y*	*z*	*atom*	*x*	*y*	*z*
S	-1.86824078	-0.37085340	-0.53097297	H	0.21314836	-2.23110204	0.14691551
C	-0.12742190	-0.14284408	-0.22465274	H	2.65538346	-1.95684377	0.50040892
C	2.61730737	0.17393814	0.19212140	H	2.27000293	2.26828378	-0.17614134
C	0.45863976	1.12287333	-0.32215777	H	3.68458085	0.29787797	0.35433393
C	0.67146512	-1.24920094	0.07216994	C	-2.57537596	0.49878388	0.89741006
C	2.04065072	-1.09007409	0.27251260	H	-2.26761050	0.02973656	1.83541203

C	1.82289476	1.28038793	-0.10252439	H	-3.66245067	0.42709946	0.80211752
H	-0.15716031	1.98056108	-0.57961283	H	-2.29300072	1.55485640	0.90285917

Table S19. Cartesian Coordinates (Å) for Phenylmethylsulfide in the Gas Phase in S_1 Calculated at PBE0/6-31G(d) Level of Theory

atom	*x*	*y*	*z*	*atom*	*x*	*y*	*z*
S	-1.84934098	0.00005181	-0.54778246	H	0.06216745	-2.19395216	-0.53825972
C	-0.13986669	0.00037518	-0.64278678	H	2.34714858	-2.15782612	0.29896103
C	2.50843444	-0.00031947	0.40620491	H	2.34803720	2.15721337	0.29942821
C	0.54203640	1.24287431	-0.32212252	H	3.52367964	-0.00062105	0.78548094
C	0.54152233	-1.24247155	-0.32241016	C	-2.35814143	-0.00004022	1.21574278
C	1.82993961	-1.21188351	0.14854027	H	-1.91696375	-0.88766460	1.68167674
C	1.83048542	1.21147952	0.14876675	H	-3.44752382	0.00003132	1.30158333
H	0.06322436	2.19460655	-0.53798342	H	-1.91677432	0.88729817	1.68202091

7. References

S1. S. Stegbauer, C. Jandl, T. Bach, *Angew. Chem. Int. Ed.* **2018**, *57*, 14593–14596.

S2. E. Januszewski, A. Lorbach, R. Grewal, M. Bolte, J. W. Bats, H.-W. Lerner, M. Wagner, *Chem. Eur. J.* **2011**, *17*, 12696–12705.

S3. . Jovaišaitė, S. Kirschner, S. Raišys, G. Kreiza, P. Baronas, S. Juršėnas, M. Wagner, *Angew. Chem. Int. Ed.* **2023**, *62*, e202215071.

S4. G. M. Sheldrick, *Acta. Cryst.* **2015**, *A71*, 3–8.

S5. G. M. Sheldrick, *Acta. Cryst.* **2015**, *C71*, 3–8.

S6. Gaussian 16, Revision C.01, M. J. Frisch, G. W. Trucks, H. B. Schlegel, G. E. Scuseria, M. A. Robb, J. R. Cheeseman, G. Scalmani, V. Barone, G. A. Petersson, H. Nakatsuji, X. Li, M. Caricato, A. V. Marenich, J. Bloino, B. G. Janesko, R. Gomperts, B. Mennucci, H. P. Hratchian, J. V. Ortiz, A. F. Izmaylov, J. L. Sonnenberg, D. Williams-Young, F. Ding, F. Lipparini, F. Egidi, J. Goings, B. Peng, A. Petrone, T. Henderson, D. Ranasinghe, V. G. Zakrzewski, J. Gao, N. Rega, G. Zheng, W. Liang, M. Hada, M. Ehara, K. Toyota, R. Fukuda, J. Hasegawa, M. Ishida, T. Nakajima, Y. Honda, O. Kitao, H. Nakai, T. Vreven, K. Throssell, J. A. Montgomery, Jr., J. E. Peralta, F. Ogliaro, M. J. Bearpark, J. J. Heyd, E. N. Brothers, K. N. Kudin, V. N. Staroverov, T. A. Keith, R. Kobayashi, J. Normand, K. Raghavachari, A. P. Rendell, J. C. Burant, S. S. Iyengar, J. Tomasi, M. Cossi, J. M. Millam, M. Klene, C. Adamo, R. Cammi, J. W. Ochterski, R. L. Martin, K. Morokuma, O. Farkas, J. B. Foresman, and D. J. Fox, Gaussian, Inc., Wallingford CT, 2019. NBO 7.0. E. D. Glendening, J. K. Badenhoop, A. E. Reed, J. E. Carpenter, J. A. Bohmann, C. M. Morales, P. Karafiloglou, C. R. Landis, and F. Weinhold, Theoretical Chemistry Institute, University of Wisconsin, Madison, WI (2018)

S7. NBO 7.0. E. D. Glendening, J. K. Badenhoop, A. E. Reed, J. E. Carpenter, J. A. Bohmann, C. M. Morales, P. Karafiloglou, C. R. Landis, and F. Weinhold, Theoretical Chemistry Institute, University of Wisconsin, Madison, WI (2018)

S8. S. J. Cassidy, I. Brettell-Adams, L. E. McNamara, M. F. Smith, M. Bautista, H. Cao, M. Vasiliu, D. L. Gerlach, F. Qu, N. I. Hammer, D. A. Dixon, P. A. Rupar, *Organometallics* **2018**, *37*, 3732–3741.

S9. (a) T. Agou, M. Sekine and T. Kawashima, *Tetrahedron Lett.*, 2010, **51**, 5013–5015.

S10. J. Jovaišaitė, S. Kirschner, S. Raišys, G. Kreiza, P. Baronas, S. Juršėnas and M. Wagner, *Angew. Chem., Int. Ed.*, 2023, **62**, e202215071.

8. NMR Spectra

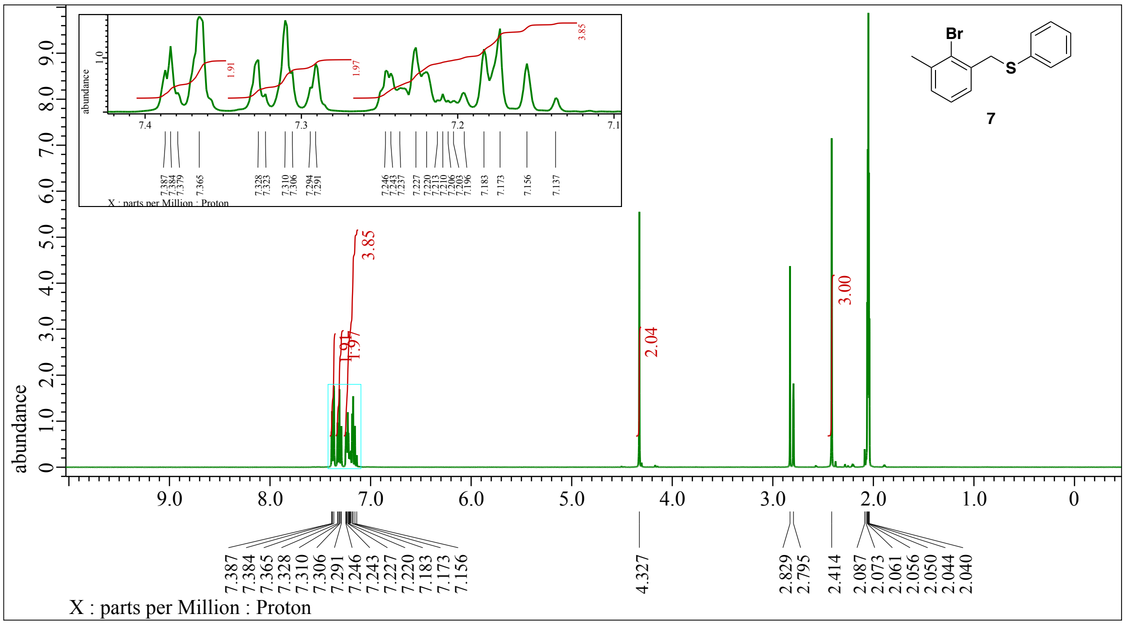

Figure S11. ^{1}H NMR spectrum of **7** (400 MHz, acetone-d_6).

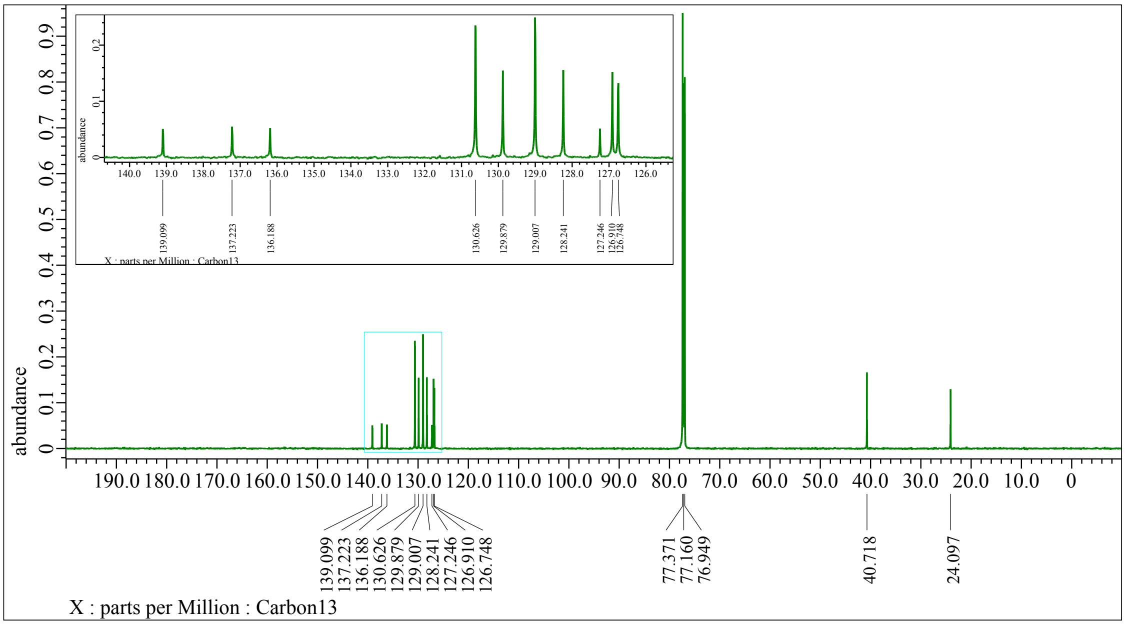

Figure S12. $^{13}C\{^1H\}$ NMR spectrum of **7** (100 MHz, $CDCl_3$).

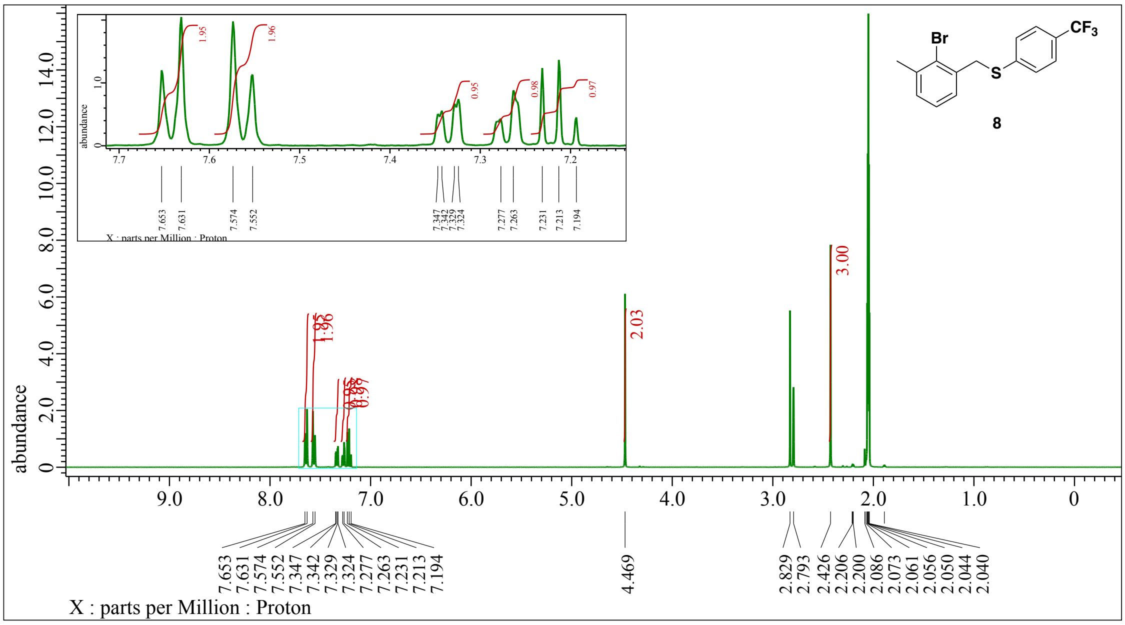

Figure S13. ^{1}H NMR spectrum of **8** (400 MHz, acetone-d_6).

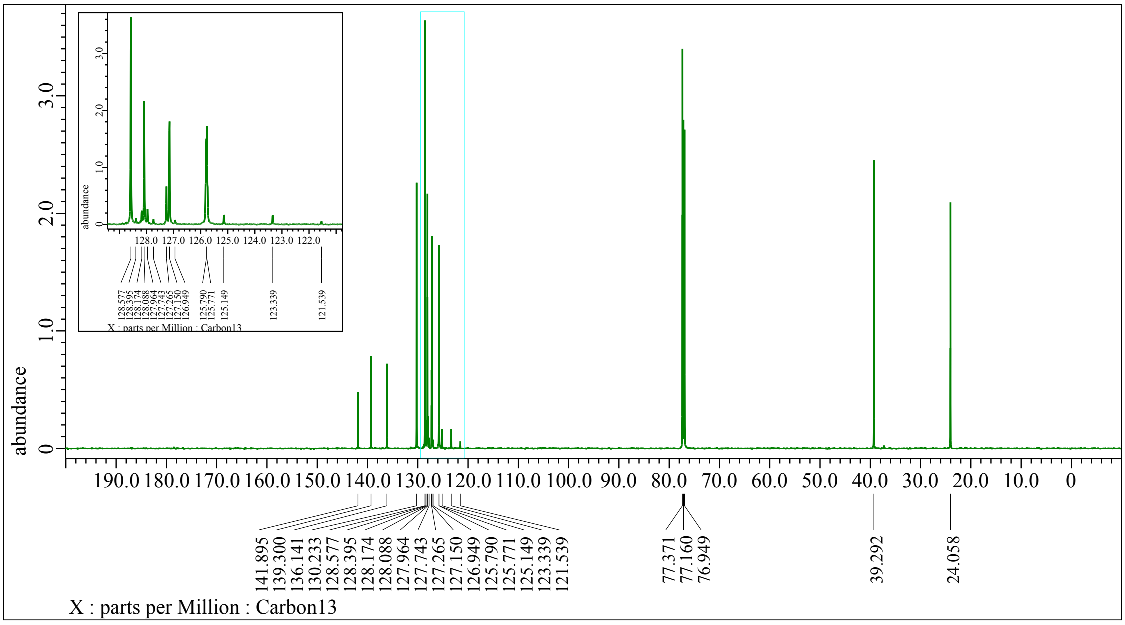

Figure S14. ^{13}C{^{1}H} NMR spectrum of **8** (100 MHz, $CDCl_3$).

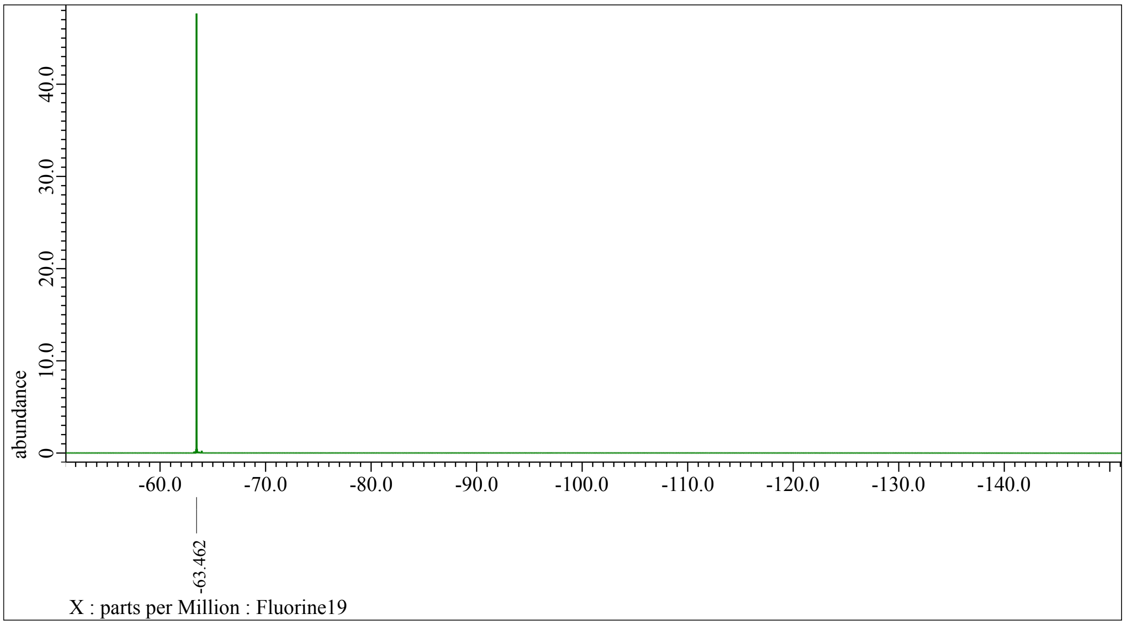

Figure S15. ^{19}F NMR spectrum of **8** (376 MHz, $CDCl_3$).

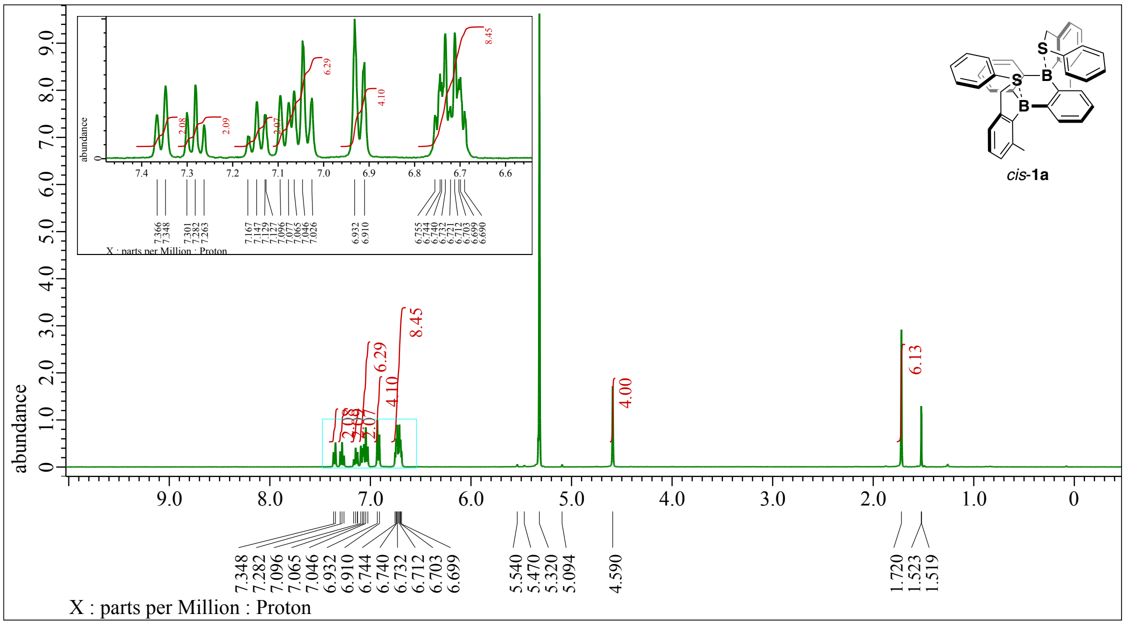

Figure S16. ^{1}H NMR spectrum of *cis*-**1a** (400 MHz, CD_2Cl_2).

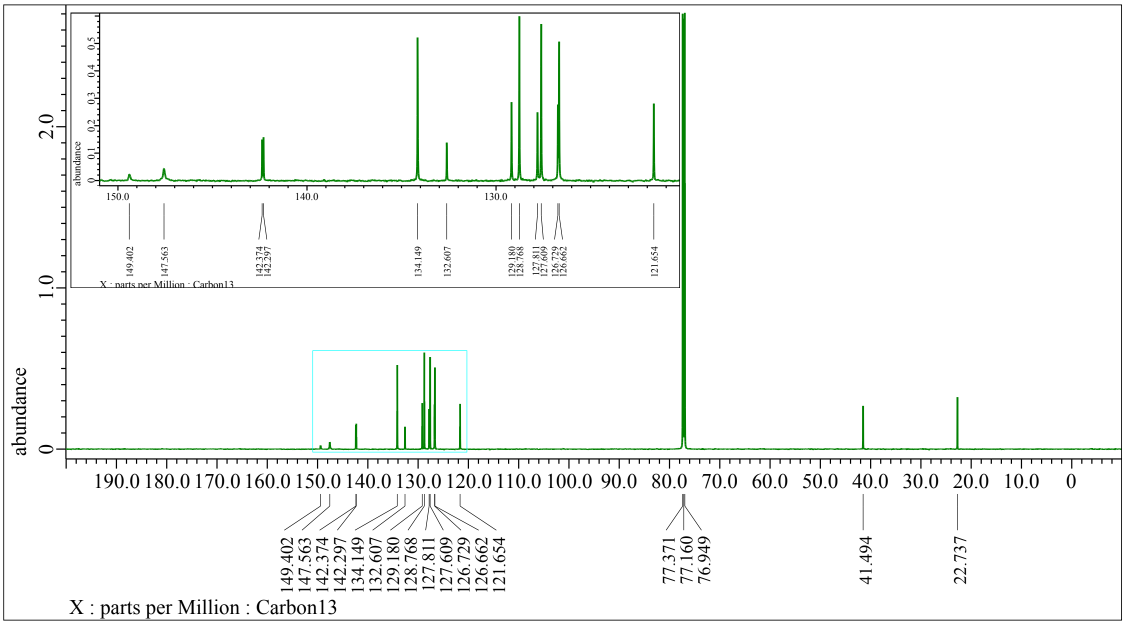

Figure S17. $^{13}C\{^{1}H\}$ NMR spectrum of *cis*-**1a** (151 MHz, $CDCl_3$).

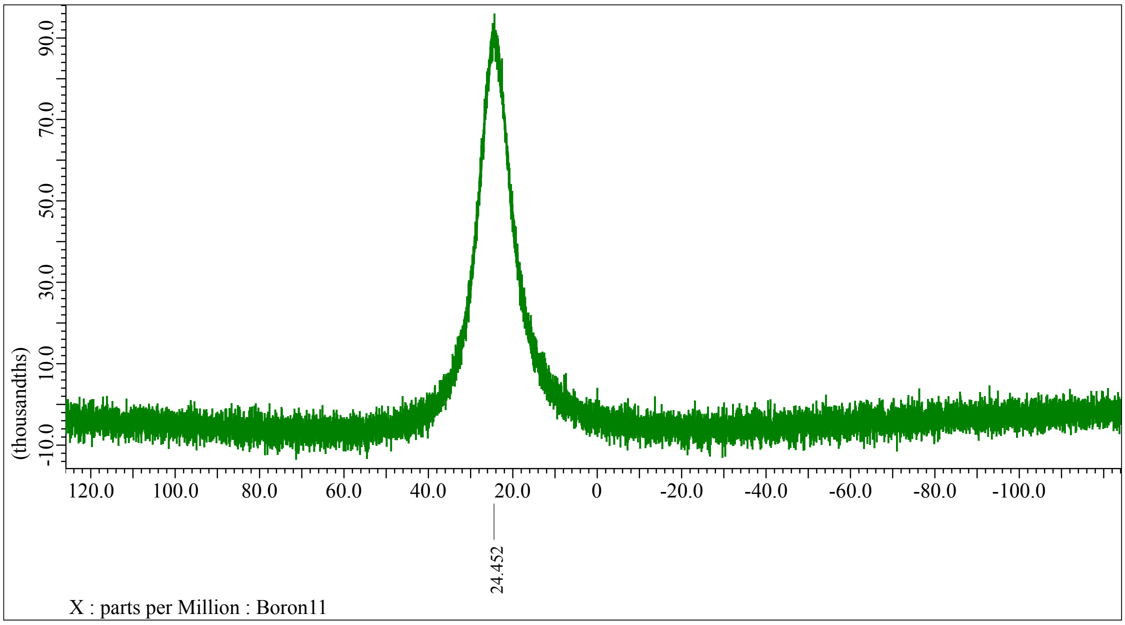

Figure S18. $^{11}B\{^{1}H\}$ NMR spectrum of *cis*-**1a** (128 MHz, $CDCl_3$).

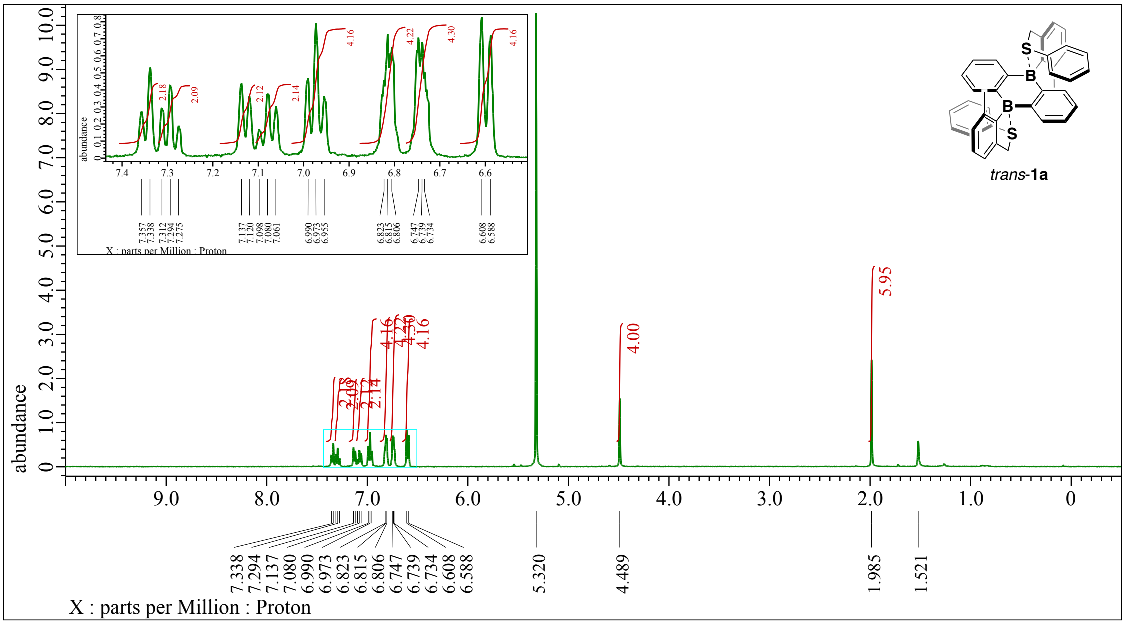

Figure S19. ^{1}H NMR spectrum of *trans*-**1a** (400 MHz, CD_2Cl_2).

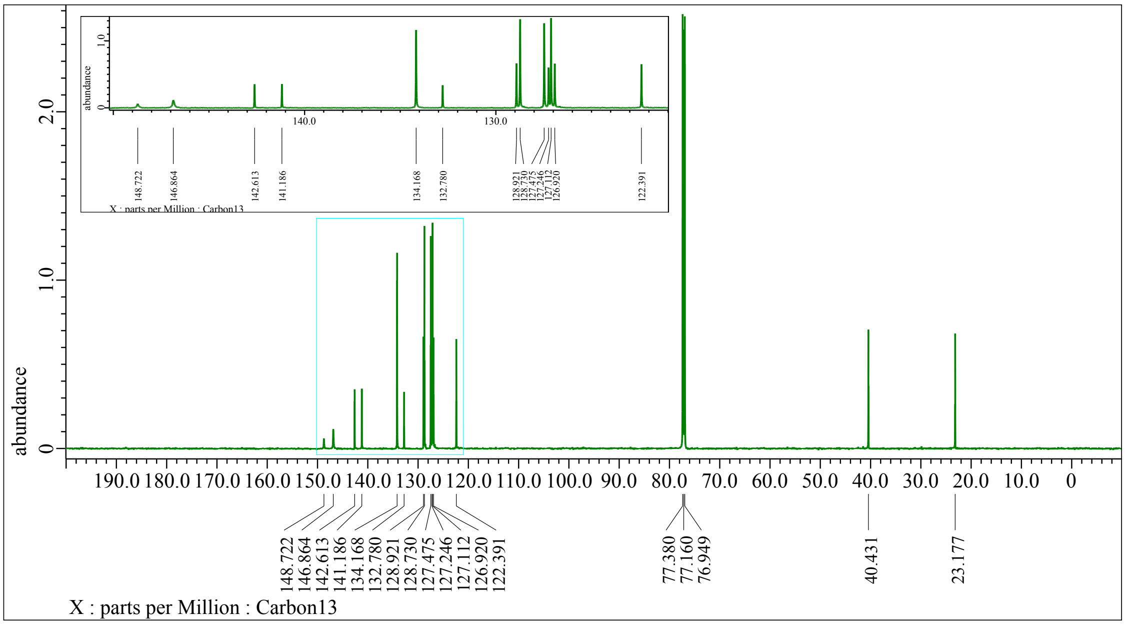

Figure S20. $^{13}C\{^{1}H\}$ NMR spectrum of *trans*-**1a** (151 MHz, $CDCl_3$).

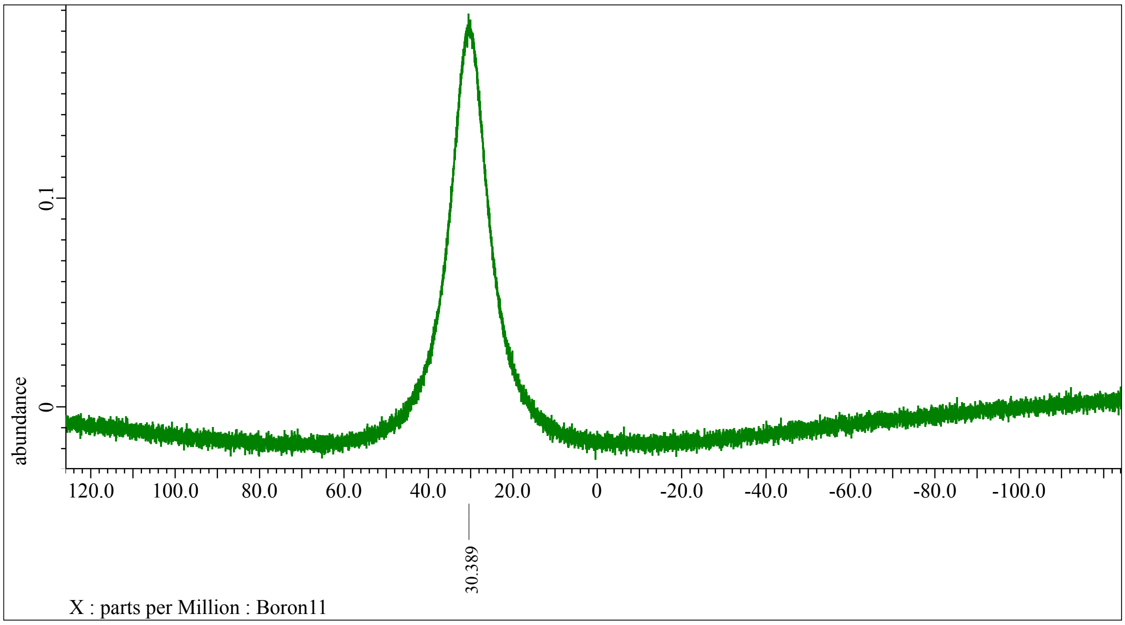

Figure S21. $^{11}B\{^{1}H\}$ NMR spectrum of *trans*-**1a** (128 MHz, $CDCl_3$).

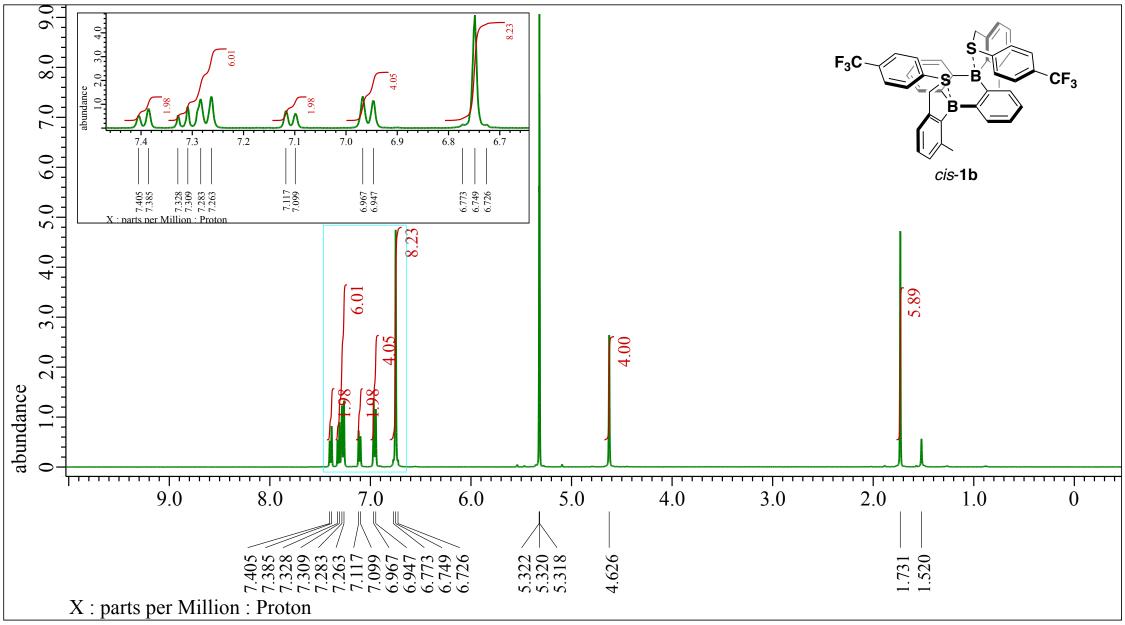

Figure S22. ^{1}H NMR spectrum of *cis*-**1b** (400 MHz, CD_2Cl_2).

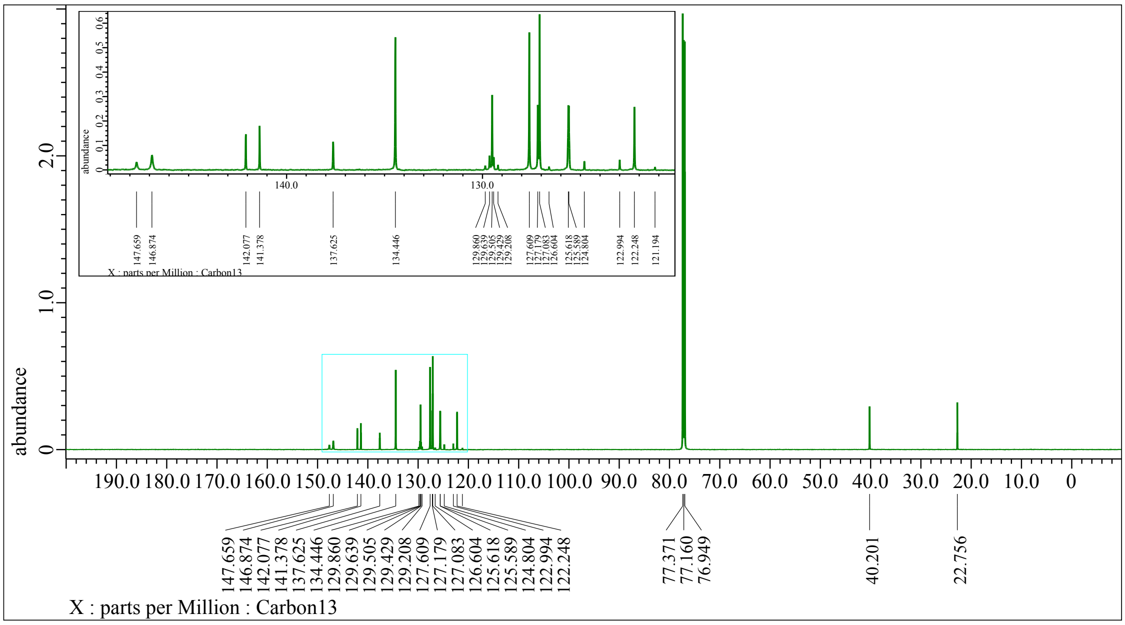

Figure S23. $^{13}C\{^1H\}$ NMR spectrum of *cis*-**1b** (151 MHz, $CDCl_3$).

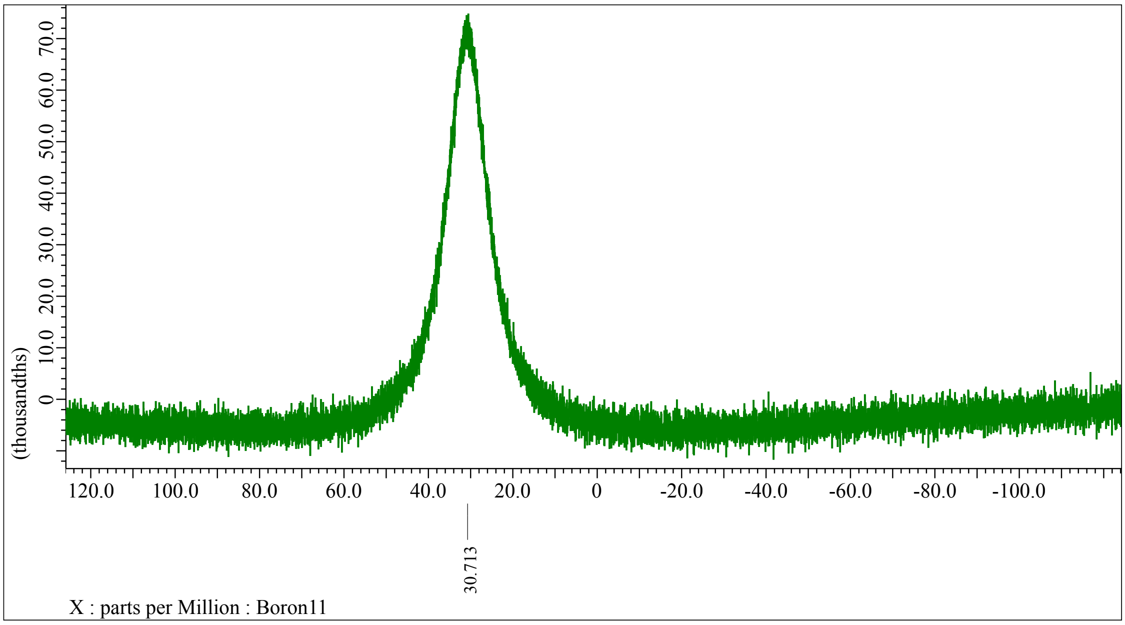

Figure S24. $^{11}B\{^1H\}$ NMR spectrum of *cis*-**1b** (128 MHz, $CDCl_3$).

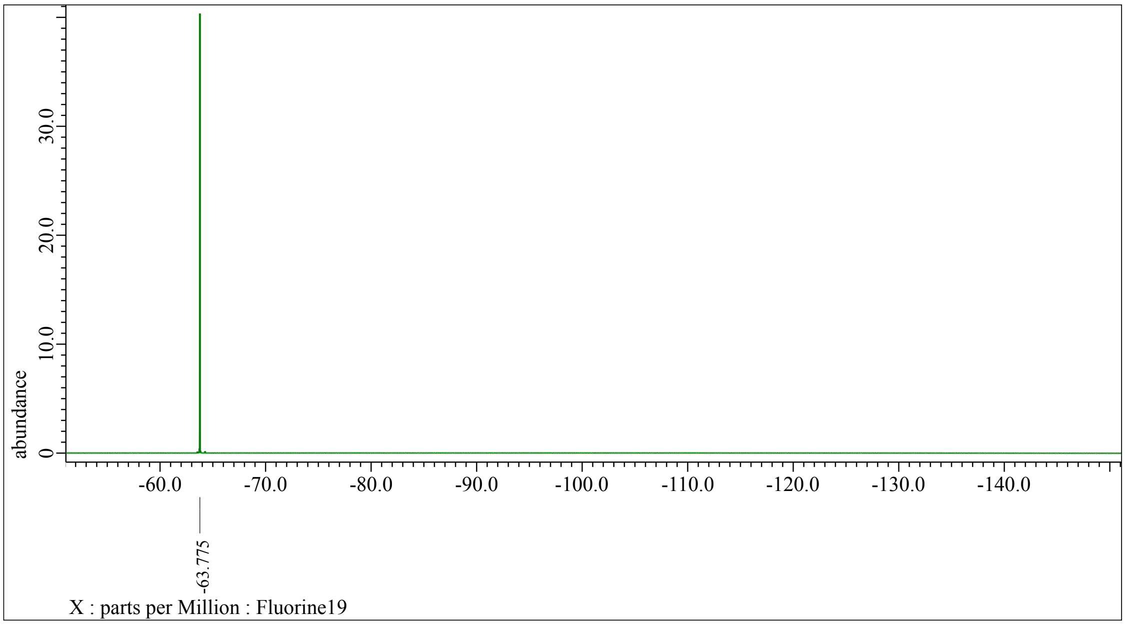

Figure S25. ^{19}F NMR spectrum of *cis*-**1b** (376 MHz, $CDCl_3$).

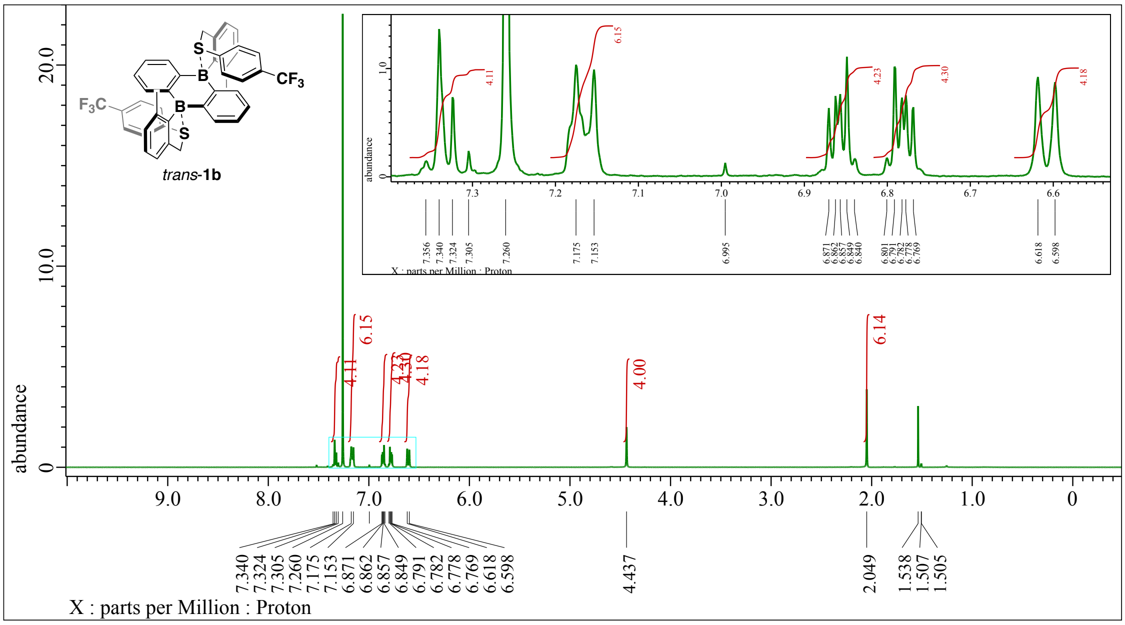

Figure S26. ^{1}H NMR spectrum of *trans*-**1b** (400 MHz, $CDCl_3$).

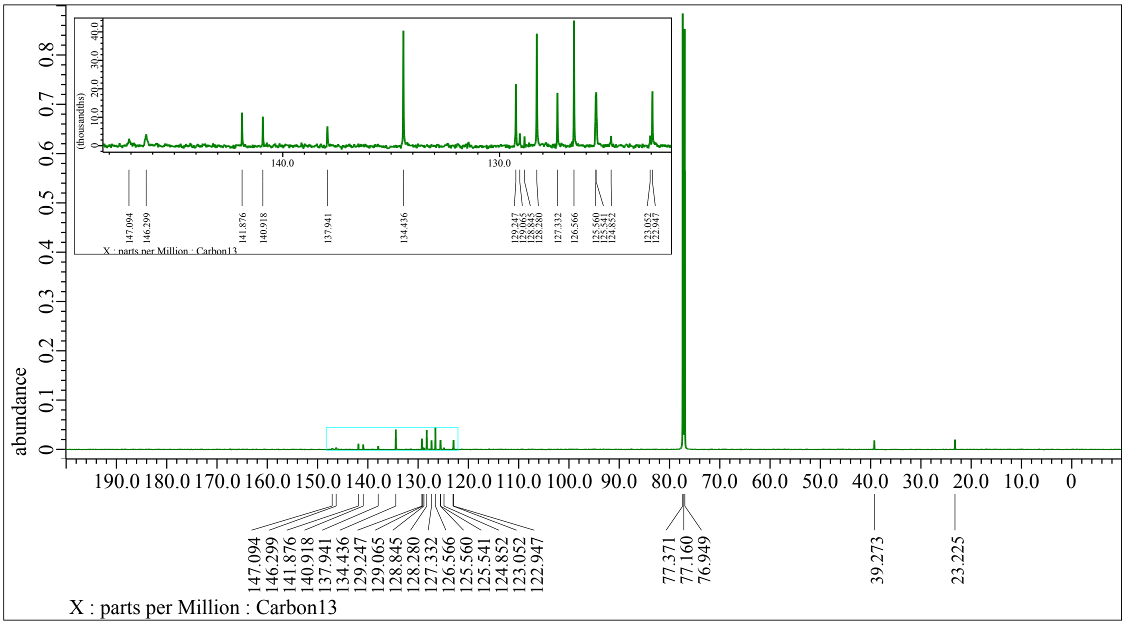

Figure S27. $^{13}C\{^1H\}$ NMR spectrum of *trans*-**1b** (151 MHz, $CDCl_3$).

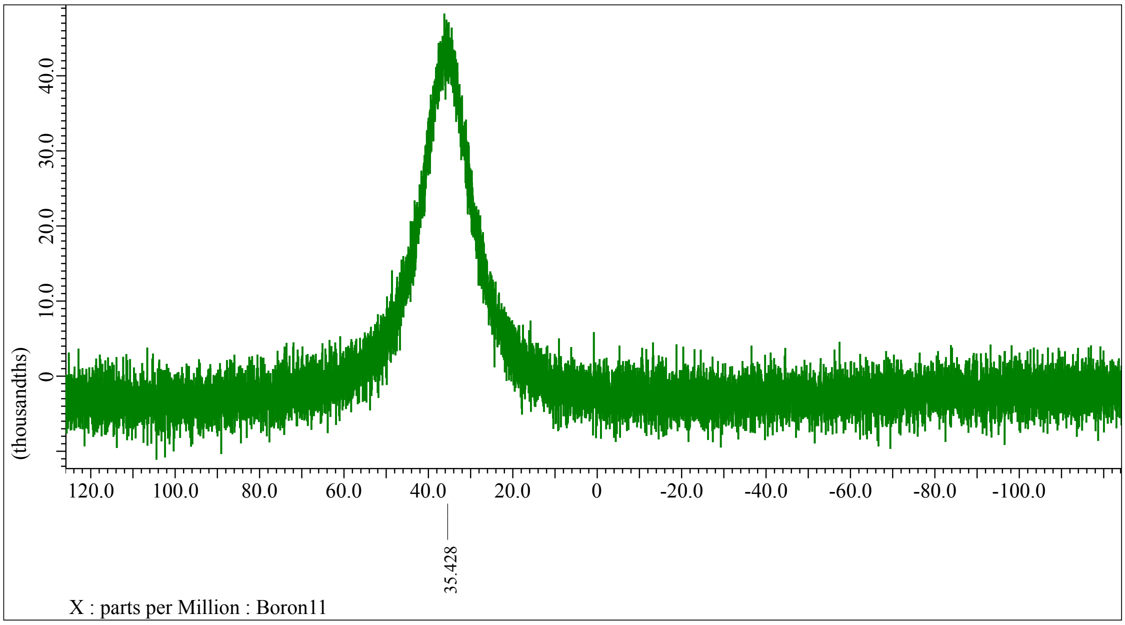

Figure S28. $^{11}B\{^1H\}$ NMR spectrum of *trans*-**1b** (128 MHz, $CDCl_3$).

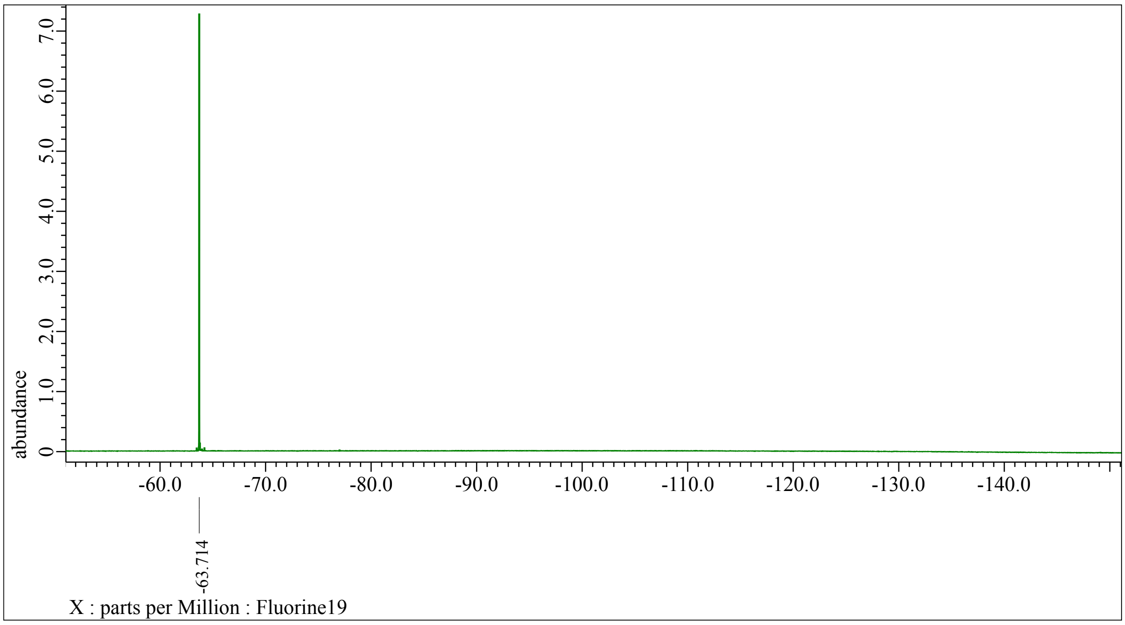

Figure S29. ^{19}F NMR spectrum of *trans*-**1b** (376 MHz, $CDCl_3$).

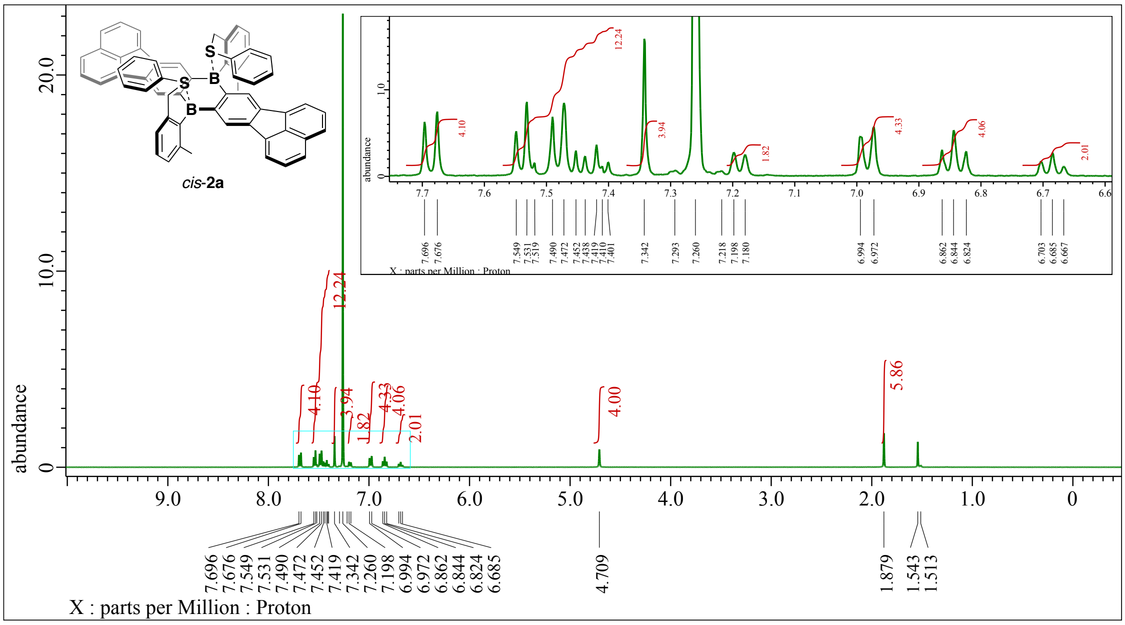

Figure S30. ^{1}H NMR spectrum of *cis*-**2a** (400 MHz, $CDCl_3$).

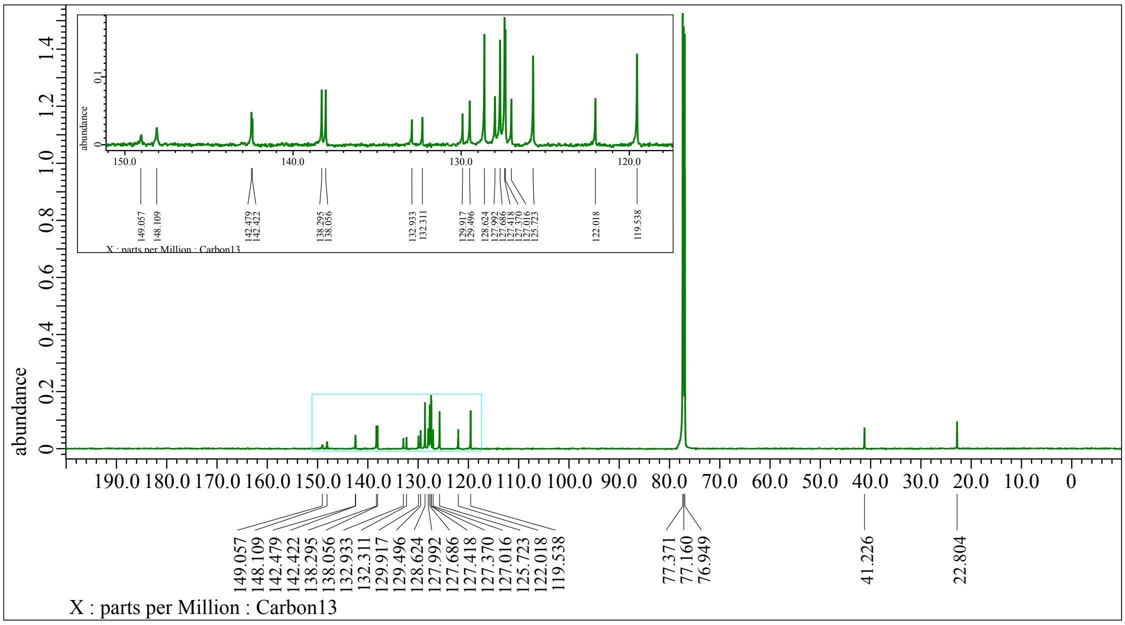

Figure S31. $^{13}C\{^1H\}$ NMR spectrum of *cis*-**2a** (151 MHz, $CDCl_3$).

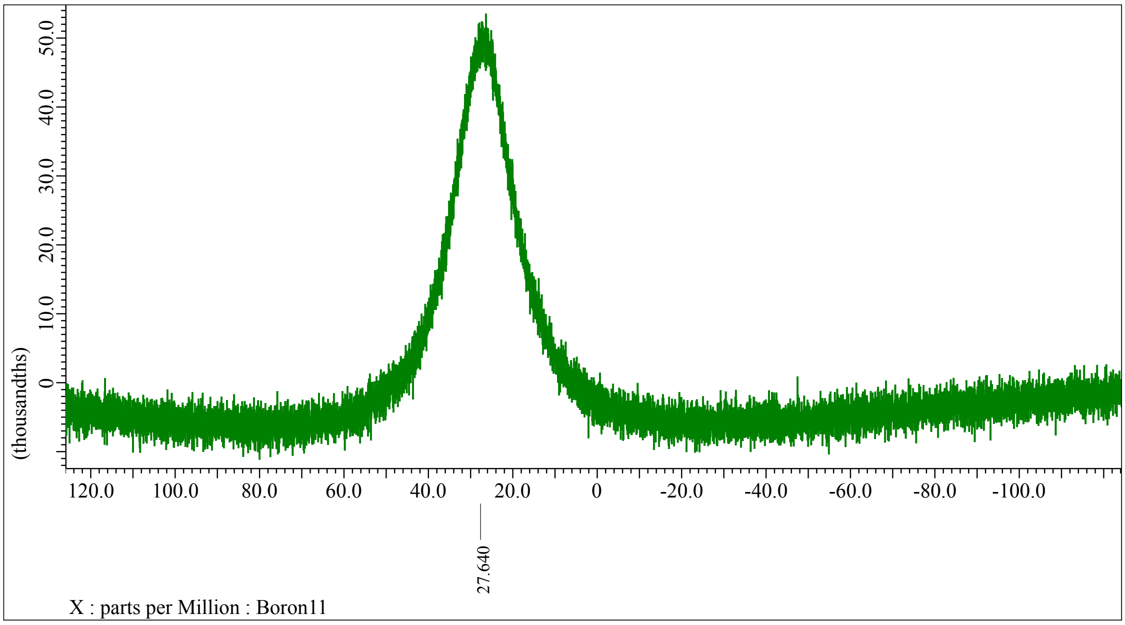

Figure S32. $^{11}B\{^1H\}$ NMR spectrum of *cis*-**2a** (128 MHz, $CDCl_3$).

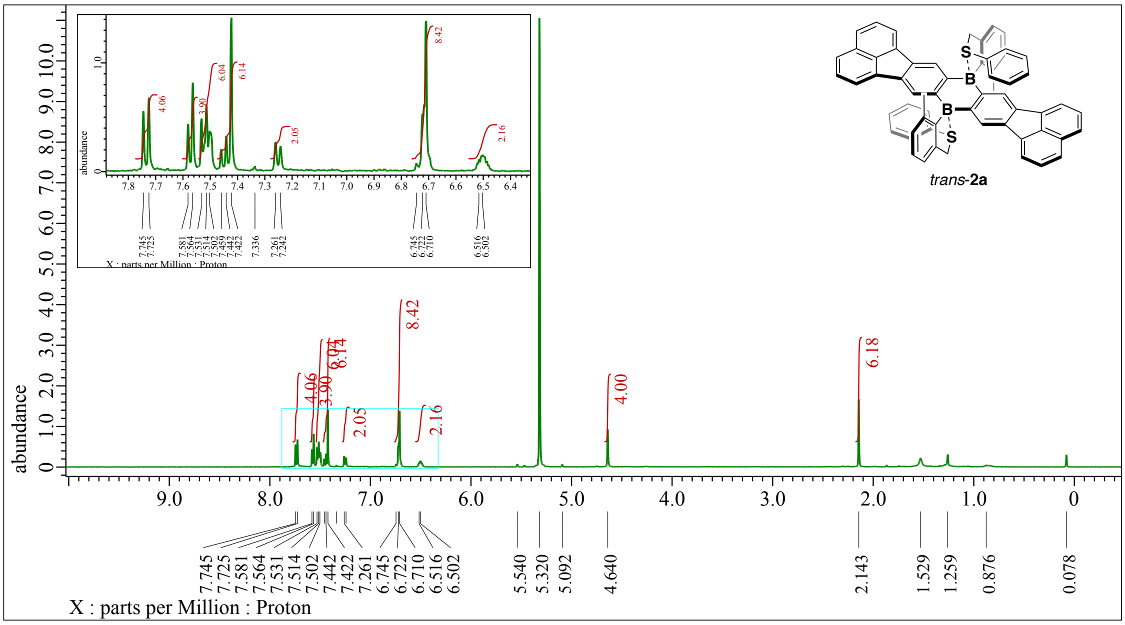

Figure S33. ^{1}H NMR spectrum of *trans*-**2a** (400 MHz, CD_2Cl_2).

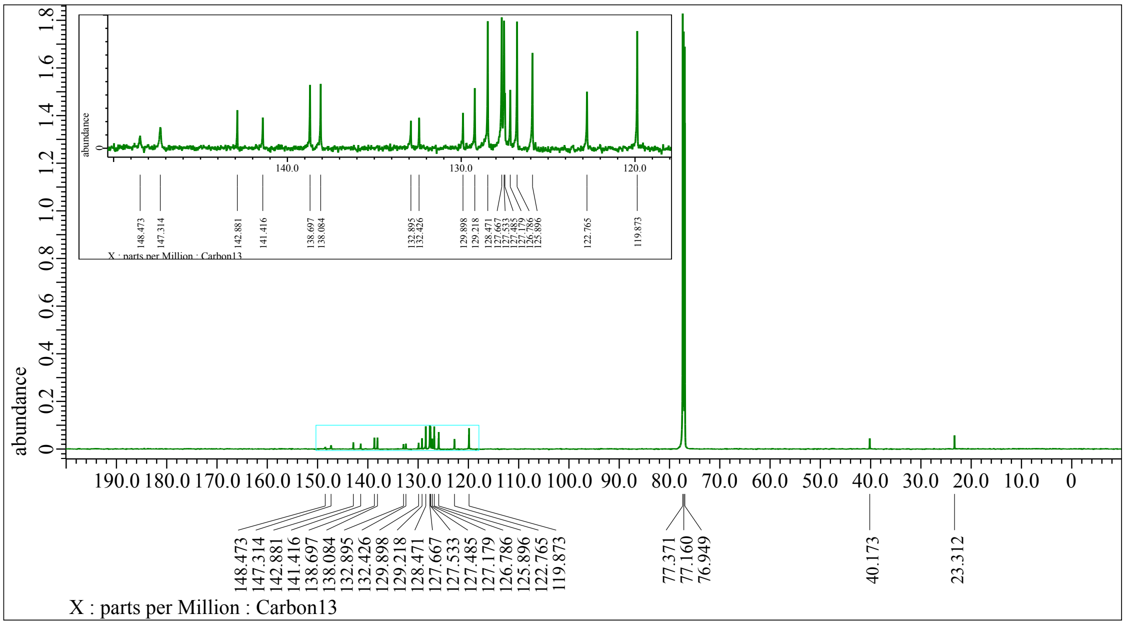

Figure S34. $^{13}C\{^{1}H\}$ NMR spectrum of *trans*-**2a** (151 MHz, $CDCl_3$).

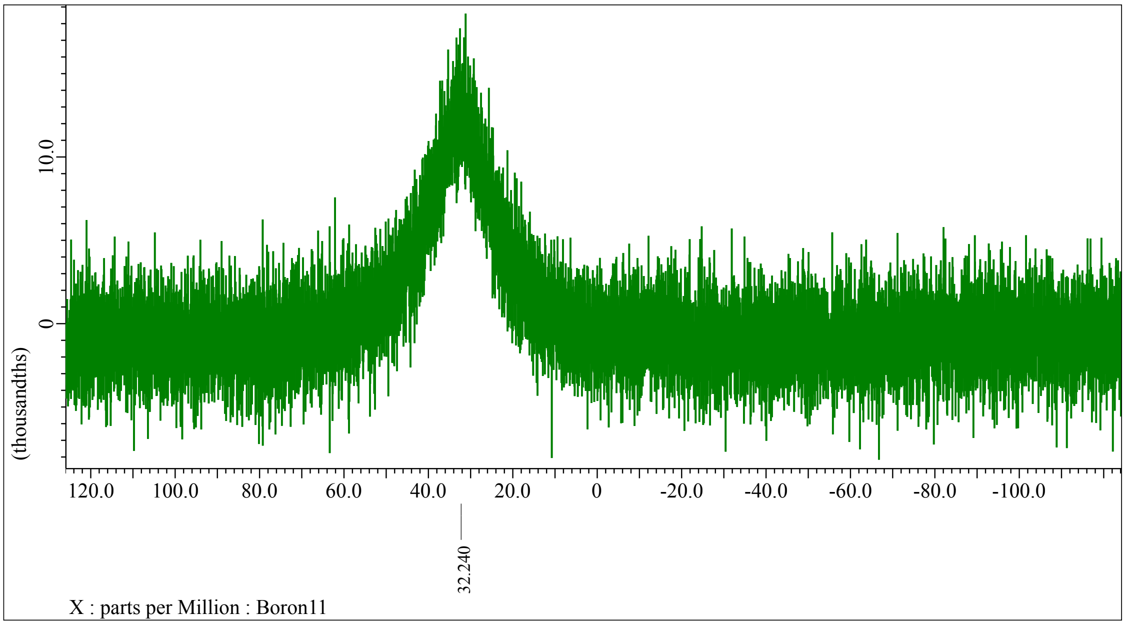

Figure S35. $^{11}B\{^{1}H\}$ NMR spectrum of *trans*-**2a** (128 MHz, $CDCl_3$).

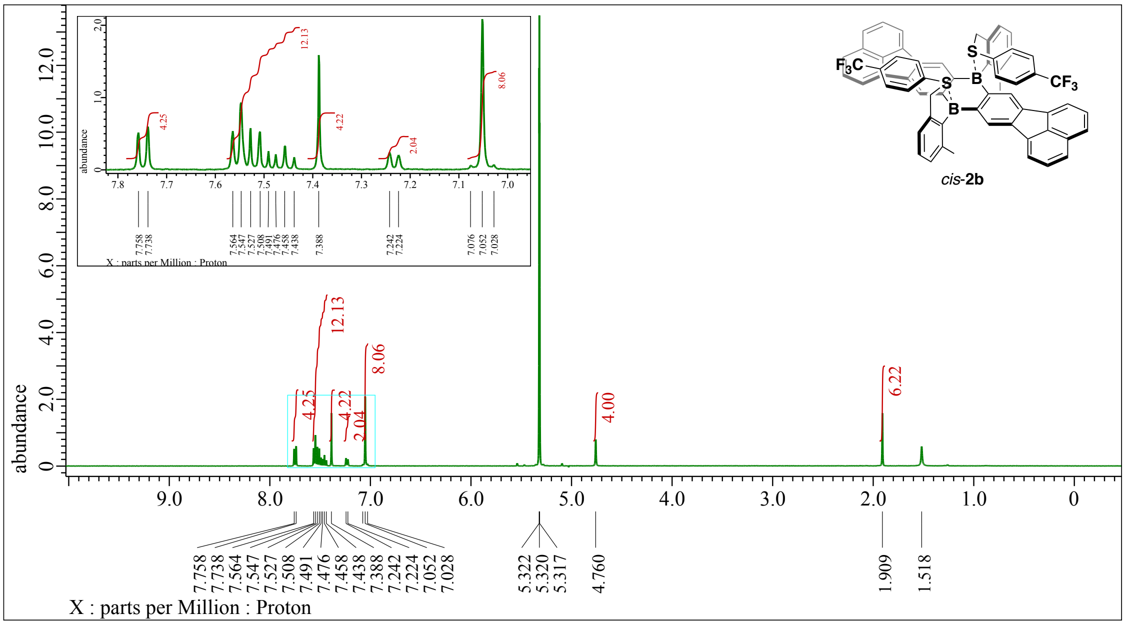

Figure S36. ^{1}H NMR spectrum of *cis*-**2b** (400 MHz, CD_2Cl_2).

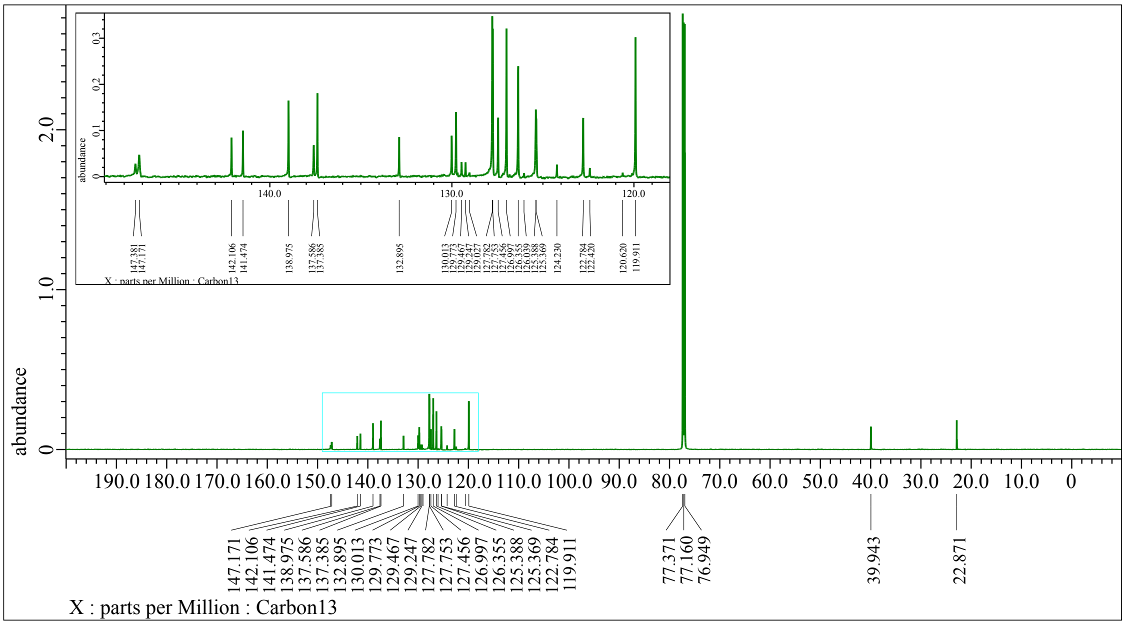

Figure S37. $^{13}C\{^1H\}$ NMR spectrum of *cis*-**2b** (151 MHz, $CDCl_3$).

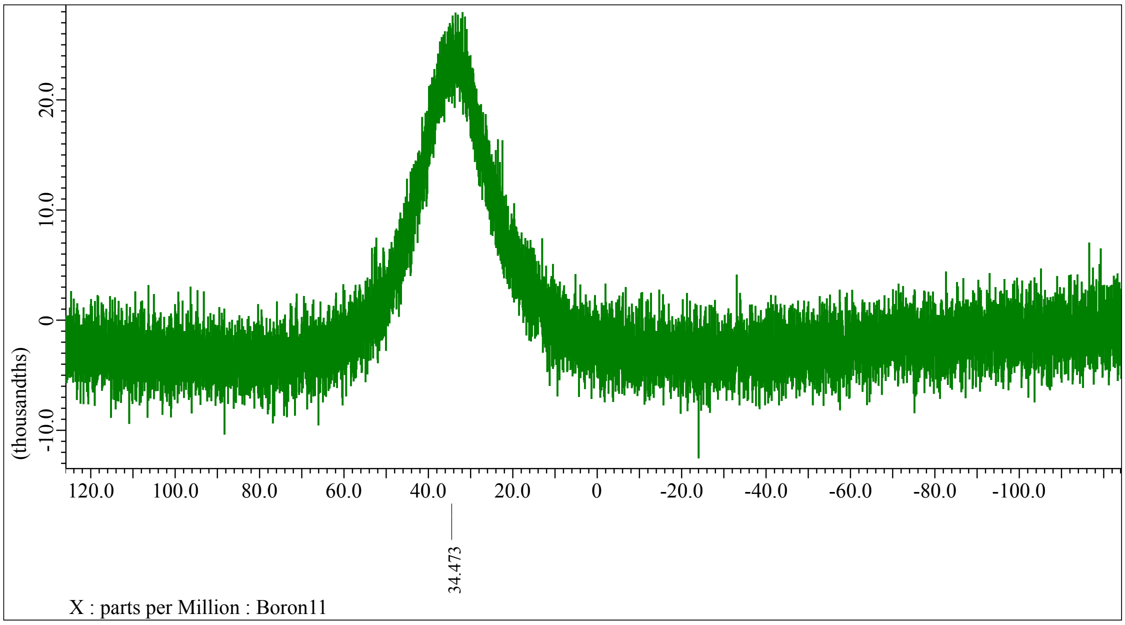

Figure S38. $^{11}B\{^1H\}$ NMR spectrum of *cis*-**2b** (128 MHz, $CDCl_3$).

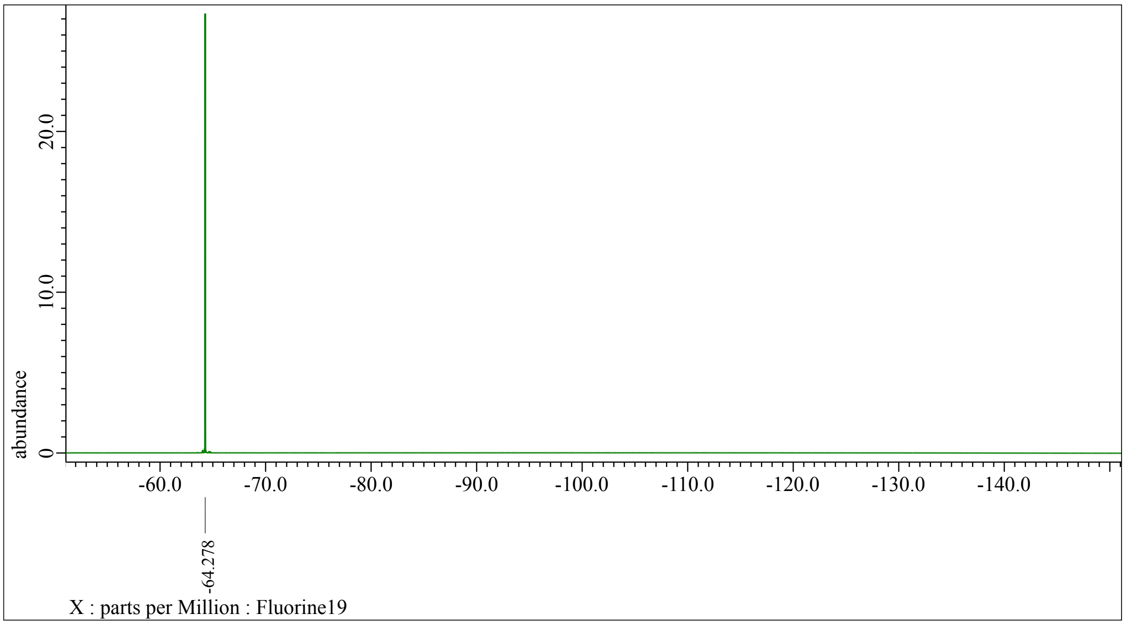

Figure S39. ^{19}F NMR spectrum of *cis*-**2b** (376 MHz, $CDCl_3$).

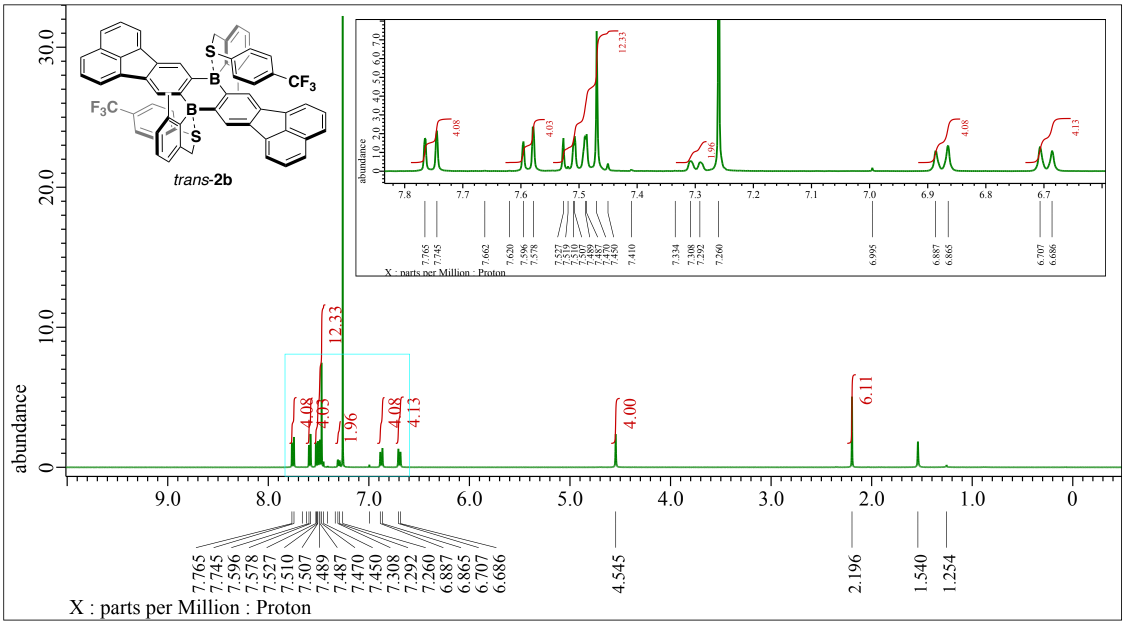

Figure S40. ^{1}H NMR spectrum of *trans*-**2b** (400 MHz, $CDCl_3$).

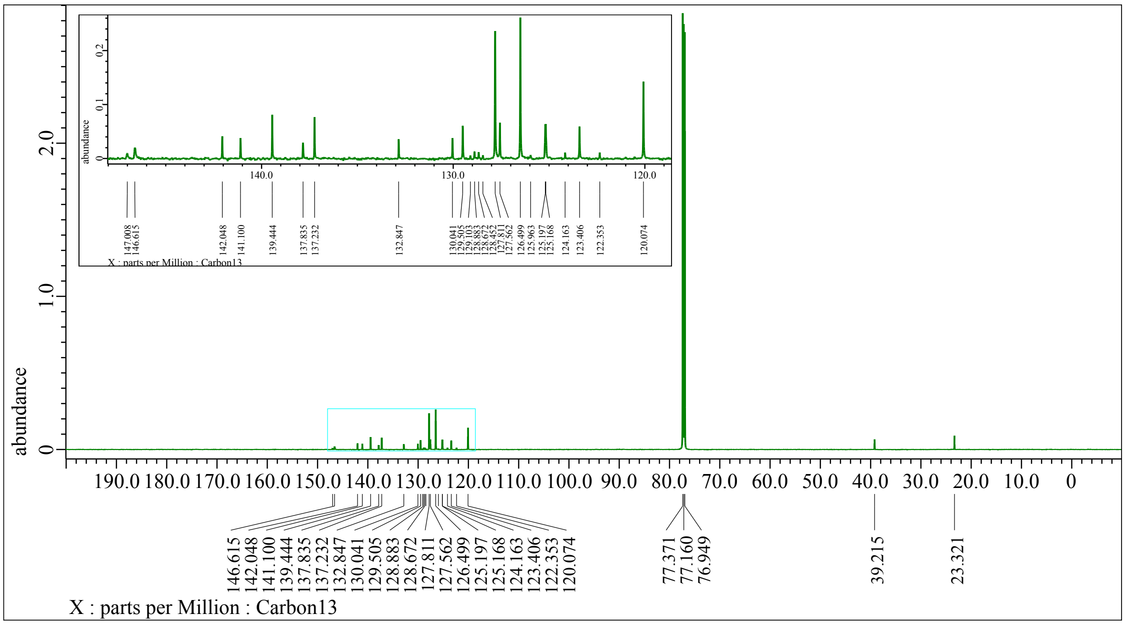

Figure S41. $^{13}C\{^{1}H\}$ NMR spectrum of *trans*-**2b** (151 MHz, $CDCl_3$).

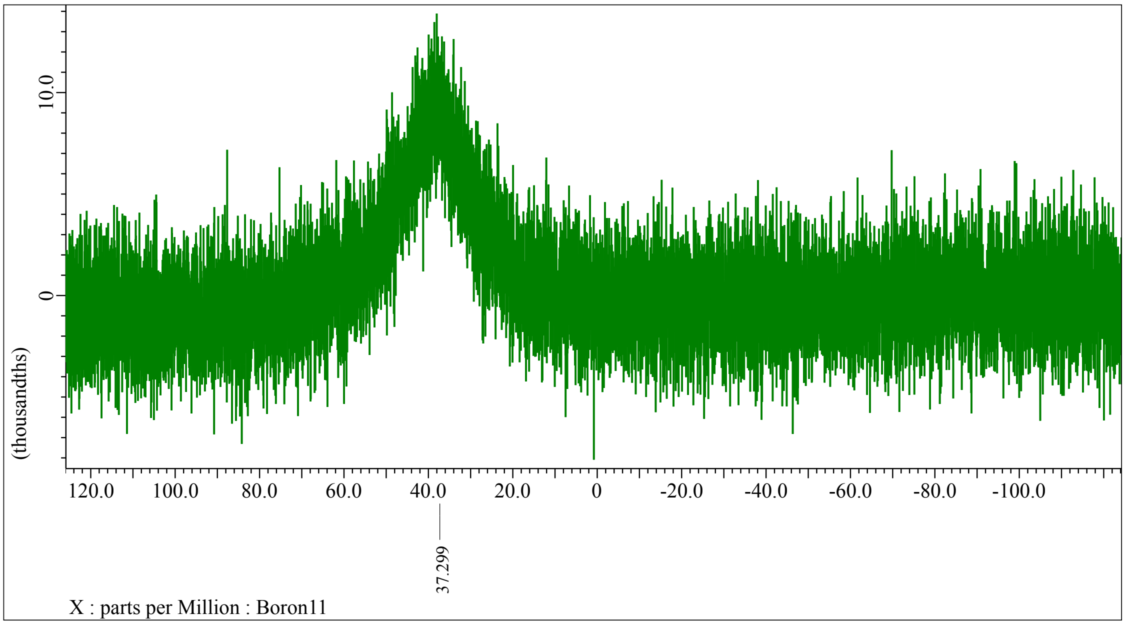

Figure S42. $^{11}B\{^{1}H\}$ NMR spectrum of *trans*-**2b** (128 MHz, $CDCl_3$).

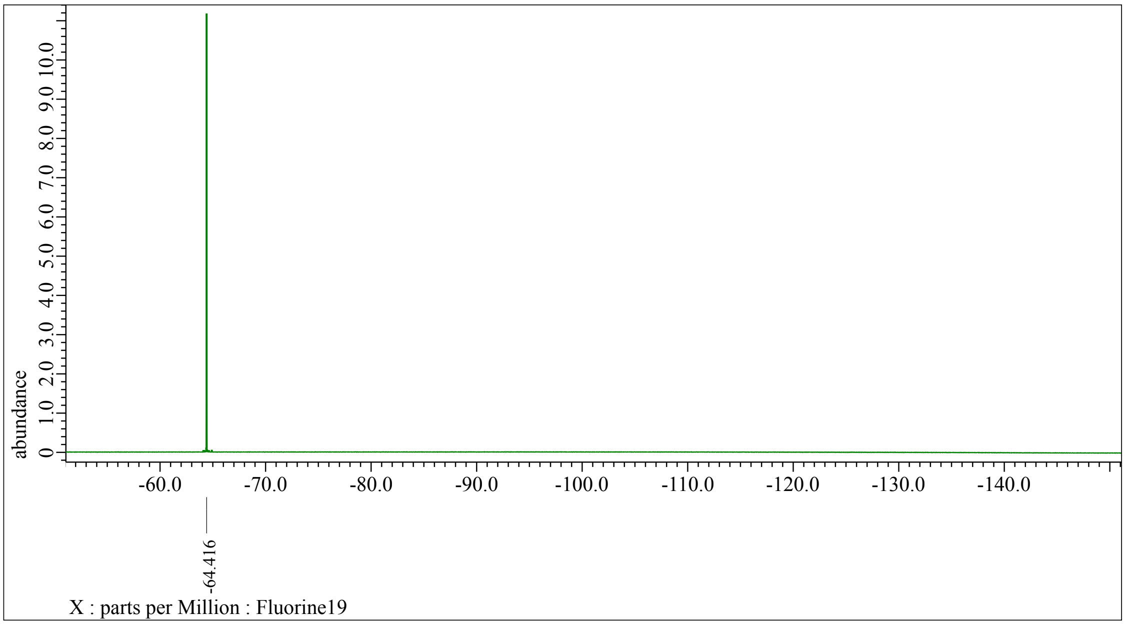

Figure S43. ^{19}F NMR spectrum of *trans*-**2b** (376 MHz, $CDCl_3$).
